# Systematic review of outcomes reported in clinical research on nephronophthisis: how do they align with SONG Kids priorities?

**DOI:** 10.1007/s00467-025-06912-0

**Published:** 2025-08-20

**Authors:** Mareike Dahmer-Heath, Sven Optenhövel, Tanja Hechler, Martin Konrad, Jens König

**Affiliations:** 1 Department of General Pediatrics, University Children’s Hospital Münster, Waldeyerstraße 22, 48149 Münster, Germany; 2 Department of Clinical Psychology and Psychotherapy for Children and Adolescents, University of Münster, Münster, Germany

**Keywords:** Rare disease, Patient-reported outcome measures. Nephronophthisis, Chronic kidney disease, Pediatric kidney disease, SONG Kids

## Abstract

**Background:**

Nephronophthisis (NPH) is a rare hereditary cystic kidney disease, characterized by a highly variable clinical and genetic presentation, accounting for up to 10% of kidney failure in children. Despite advances in understanding its molecular basis and phenotypic spectrum, no causative therapies exist, and clinical trials remain absent. To support future treatment development, patient-reported outcome measures (PROMs) tailored to NPH should be defined to prioritize outcomes meaningful to patients and families.

**Objectives:**

This study aimed to analyze the use of clinical data, surrogate parameters, and patient-reported outcomes in NPH research to date, with a focus on the Standardized Outcomes in Nephrology (SONG) project outcomes validated for children with chronic kidney disease (SONG Kids).

**Data sources:**

A systematic search of the MEDLINE database was conducted for NPH studies.

**Study eligibility criteria:**

Studies published after 1988, written in English, reporting at least one human clinical outcome, with a sample size of *n* ≥ 4, and using original data were considered eligible.

**Results:**

A total of 1066 records were retrieved through the search, of which 821 full-text reports were assessed for eligibility. Of these, 90 studies met eligibility criteria and were included in the review. While 100% of the studies reported clinical outcomes and 85% included surrogate parameter, only 41% examined patient-reported outcomes. Overlap between the SONG Kids outcome set and the outcomes identified in this study was moderate. Only 20 studies reported more than one SONG core outcome, while 24% and 66% of studies reported at least one middle tier or outer tier outcome, respectively. None of these studies used instruments validated for NPH.

**Limitations:**

The majority of studies focused primarily on molecular and genetic aspects, with clinical outcomes addressed only as a secondary consideration. The review incorporated only one prospective study, while the remaining studies were retrospective in nature. Differentiation between outcomes reported by children and those reported by parents was not possible in the included studies; this important distinction must be taken into account in the development of future PROMs for NPH.

**Conclusions and implications of key findings:**

Studies in NPH addressed both clinical outcomes and surrogate parameters, but there is a notable absence of measures related to life participation and patient-reported outcomes. Disease group-specific instruments fall short in adequately reflecting the symptoms of individual diseases, emphasizing the necessity for the development of disease-specific PROMs for NPH.

Open Science Framework (OSF) registration: https://doi.org/10.17605/OSF.IO/658BR

**Graphical abstract:**

**Figure:**
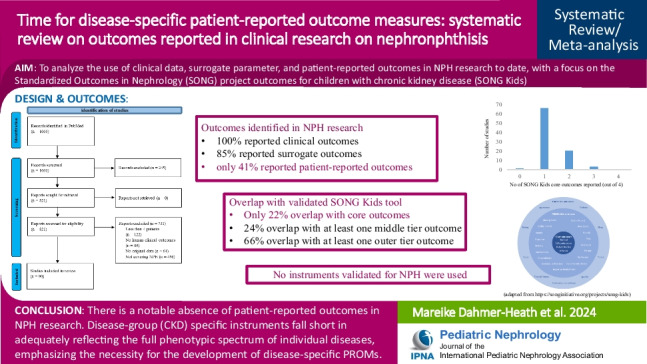
A higher resolution version of the Graphical abstract is available as Supplementary information

**Supplementary Information:**

The online version contains supplementary material available at 10.1007/s00467-025-06912-0.

## Introduction

### Nephronophthisis

Nephronophthisis (NPH) encompasses a clinically and genetically heterogeneous group of hereditary cystic kidney disorders representing the most frequent monogenetic cause of kidney failure in children and young adults [1]. The hallmarks of NPH are a tubular urine concentrating deficiency presenting as polyuria/polydipsia and an undetected slowly deteriorating decline of kidney function. Depending on the age of onset of kidney failure, three clinical entities are distinguished: juvenile, infantile, and adolescent NPH. The classic juvenile NPH is by far the most common form and leads to kidney failure at a median age of 13.5 years [2]. The rarer adolescent form has a slower progression and results in kidney failure around the age of 19. Patients with infantile NPH require kidney replacement therapy before the age of four, typically around the 8th month of life [3]. Disease-causing variants in more than 20 genes have been identified. A homozygous deletion of the *NPHP1* gene on chromosome 2q12-q13 is the most common genetic cause of NPH, accounting for 20–60% of all genetically identified patients [4]. Genetic variants of all other NPHP genes account for 1% or less each [5, 6]. Depending on the underlying molecular variant, NPH can manifest as an isolated kidney disease or in combination with alterations in other organ systems as part of syndromic complex disorders. Extrarenal manifestations mainly affect the eyes, liver, central nervous system (CNS), and skeletal system. Less frequently, heart defects or laterality defects are observed [7]. Syndromic disorders that are characterized by the kidney phenotype of NPH include Senior–Løken syndrome, Joubert syndrome, Meckel–Gruber syndrome, and the group of short-rib polydactyly syndromes [5]. Clinical management and personalized counseling are major challenges associated with NPH due to the wide phenotypic variability, overlapping clinical features, and the consequential unpredictability of the individual disease progression. The prognostic uncertainty puts a huge psychological burden on pediatric patients and their families facing a potentially life-threatening disease of unclear onset and extent severely affecting their quality of life.

### Therapeutic targets in rare disease

NPH is classified as a rare disease with incidences ranging from 1 per 50,000 to 1 per 1,000,000, depending on ethnicity and the underlying disease-causing variant, making systematic research extremely difficult [8–10]. Clinical and molecular research in the field of NPH has been dynamic in recent years, leading to enhancement of the genetic landscape, definition of genotype–phenotype correlations, expansion of the phenotypic spectrum, and identification of a first disease-specific biomarker [2, 11–16]. However, no clinical trials have been conducted with causative therapies still missing. To remedy this situation, the TheRaCil (therapeutics for renal ciliopathies, theracil.eu) interdisciplinary network has been initiated with the goal to create safe and efficient treatment approaches for pediatric patients with renal ciliopathies including NPH. Prominent European ciliopathy databases are being combined to grant access to an extensive pool of clinical, biochemical, and genetic data sourced from a diverse global population of patients with renal ciliopathy [17–20]. One of the work packages of the TheRaCil project is focusing on the identification, definition, and prioritization of patient-reported outcome measures (PROMs) for future clinical trials.

### Patient-reported outcome measures (PROMs)

It is a requirement to establish outcome measures, including the definition of PROMs, in order to define therapeutic targets in NPH [21]. Outcomes that reflect authentic benefits from a patient perception are of pivotal importance as rare disease therapies should be established to benefit patients and not just treat their disease [22]. The purpose of PROMs is to put patients, their families, and caregivers in the center of decisions concerning the most valuable measures in health assessment rather than leaving valuations exclusively to physicians. It is a necessity to include patient opinions in benefit-risk assessments of novel drugs to comply with regulatory authorities, secure interest from drug development companies, and please the rare disease research communities.

### Standardized Outcomes in Nephrology (SONG) Kids project

**Fig. 1:**
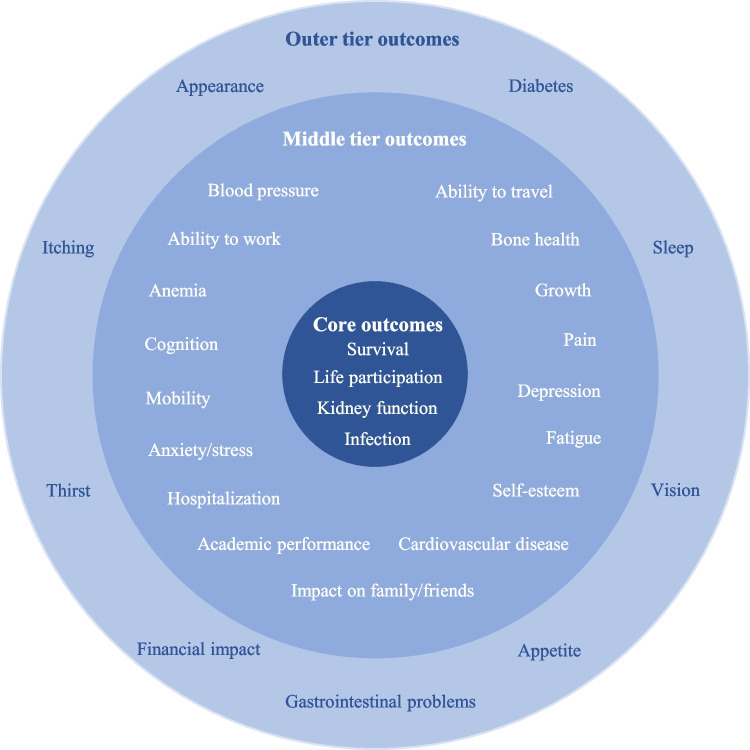
SONG Kids core, middle, and outer tier outcomes (adapted from https://songinitiative.org/projects/song-kids/)

By focusing on outcomes that are meaningful and relevant to patients, their families, and healthcare providers, the SONG initiative aims to enhance the quality and relevance of research findings, ultimately supporting informed treatment decisions [23]. The SONG Kids initiative defined a core set of outcomes and outcome measures applicable across the full spectrum of kidney disease in children for clinical trials and research [24]. These outcomes were developed based on the shared priorities of patients, caregivers, clinicians, researchers, policymakers, and other stakeholders [25–29]. The outcome domains were categorized into three ranks: core outcomes (defined as “*critically* important to *all* stakeholder groups”), middle tier outcomes (defined as “*critically* important to *some* stakeholder groups”), and outer tier outcomes (defined as “*important* to some or all stakeholder groups”) (Fig. 1). Following the SONG Kids approach, TheRaCil aims to establish PROMs in the setting of NPH.

As the first step toward establishing a standardized set of PROMs in NPH, this systematic review aimed to examine the range and heterogeneity of clinical outcomes, surrogate parameters, and patient-centered outcomes reported in existing NPH research, with special consideration given to the SONG Kids outcome set for children with chronic kidney disease as a potential reference framework.

## Methods

In order to guarantee quality and transparency of the process, the methodology was based on the Preferred Reporting Items for Systematic Reviews and Meta-Analyses (PRISMA) statement for reporting systematic reviews (Online Resource 1) [30, 31].

### Electronic search strategy/selection process

The PubMed database was systematically searched by one reviewer (MD-H) using the following search string: (nephronophthisis OR NPH OR NPHP) AND (child OR young adult OR adolescent OR pediatric). The last search update was done on 30.10.2024 (SO). Search terms were defined after reviewing titles, abstracts, and keywords of relevant studies for commonly used terms in this line of research. A pilot search was conducted to ascertain whether the aforementioned publications were identified by the developed search strategy. Abstracts were screened by MD-H, and full texts of reports deemed eligible were assessed and checked against predefined eligibility criteria. A random selection of abstracts excluded by MD-H was double-checked by SO in order to reduce the risks of missing relevant records. Reports considered eligible by MD-H after full text assessment were reviewed by SO. In the event of disagreement, consensus was reached through discussion between three researchers (MD-H, SO, JK). Remaining reports were included in the analysis. References were managed using EndNote version 20, Build 14,675.

### Eligibility criteria

The electronic search initially included all studies, but those published before 1988 were excluded. This cutoff was selected to align with the period leading up to the molecular mapping of the first NPH-related gene to chromosome 2q12-q13, which occurred 5 years later [32]. Publications in languages other than English were excluded. Studies with sample sizes fewer than four participants were also excluded to eliminate simple case reports. Only studies meeting all of the following critera were considered eligible for inclusion: (1) inclusion of patients with a confirmed diagnosis of NPH; (2) reporting of at least one clinical outcome; and (3) presentation of original data.

### Data extraction and statistical analysis

Data were extracted by two researchers (MD-H, SO) using a pre-developed data extraction form including the following items:

- Author, publication year, country, study type
- Sample size, age, gender, medical conditions
- Outcomes

Following data extraction, reported outcomes of all studies were reviewed by the research team. In instances where studies used varying terms for the same outcome, harmonization was achieved by selecting the term that was utilized with greatest frequency. Subsequently, outcomes were grouped based on the affected organ/organ system and SONG Kids outcome categories [24]. Descriptive statistics and graphs were generated using Microsoft Excel 2016 (Microsoft Corporation, Redmond, Washington, USA). Statistical analyses were conducted using IBM SPSS Statistics version 29. Data were controlled for normal distribution using the Shapiro–Wilk and Kolmogorov–Smirnov tests with Lilliefors correction. As both Shapiro–Wilk and Kolmogorov–Smirnov tests indicated that the dataset was not normally distributed (Online Resource 2), the Kruskal–Wallis test was used to test for significant differences between publications with good, moderate, and poor quality ratings regarding the number of reported SONG outcomes per category and reporting of clinical outcomes, surrogate parameters, and patient-reported outcomes. When significant differences were identified, post hoc pairwise comparisons were conducted using Dunn’s test with Bonferroni correction to adjust for multiple comparisons.

### Quality assessment

The quality of non-intervention studies was evaluated using the Critical Appraisal Checklist [33]; quality of intervention studies was assessed using the Risk of Bias 2 tool (for randomized trails) or ROBINS-I tool (for non-randomized trails) [34, 35] (Table 1). Quality of case reports encompassing more than four patients was assessed using the Joanna Briggs Institute (JBI) Critical Appraisal Checklist for Case Reports [36]. For evaluating retrospective genetic studies, a modified version of the CASP Checklist for cohort studies [33], or, in instances where the reporting style was conceptualized akin to case studies, the JBI Critical Appraisal Checklist for Case Reports [36] was used. The following items were removed from the CASP Checklist for cohort studies due to lack of fit: Item 3: “Was the exposure accurately measured to minimize bias?” (no exposure in the studies); Item 6a: “Was the follow up of subjects complete enough?”; and Item 6b: “Was the follow up of subjects long enough?” (due to the retrospective nature of the studies, no follow-ups were possible).

**Table 1:** Summary of quality assessment tools

Study quality assessment tool	Target study designs	Short description
Critical Appraisal Checklist for Cohort Studies	Cohort studies	Evaluates study quality based on 12 questions, including a.o. cohort recruitment, outcome measurement, results reporting implications
Risk of Bias 2 (RoB 2)	Randomized clinical trials	Evaluation of risk of bias on five domains: the randomization process, deviations from intended interventions, missing data, outcome measurement, and reporting of results
Risk of Bias in Non-randomized Studies of Interventions (ROBINS-I)	Non-randomized intervention studies	Assesses risk of bias on seven domains: bias due to confounding, participant selection, classification of interventions, deviation from intended interventions, missing data, outcome measurement, and selection of reported results
JBI Critical Appraisal Checklist for Case Reports	Case reports	Evaluates study quality based on eight questions mainly focused on transparency and comprehensiveness of reporting

## Results

### Electronic search results

**Fig. 2:**
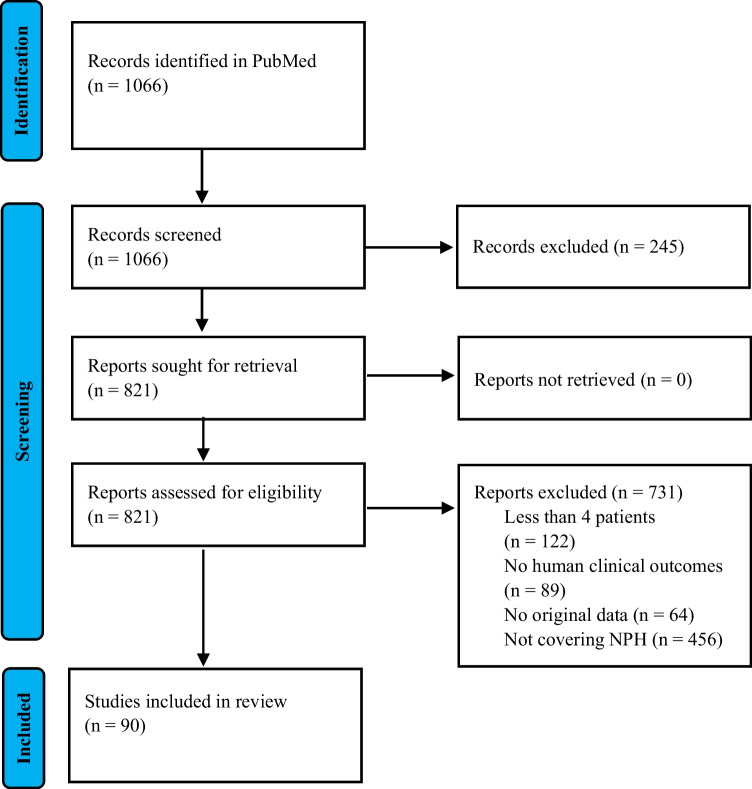
Selection of eligible papers (NPH, nephronophthisis)

The initial electronic search of the PubMed database was conducted in January 2023, yielding 1023 records. A final search in October 2024 identified an additional 43 records, resulting in a total of 1066 records (Fig. 2). After removing 245 records during title and abstract screening, 821 reports were assessed for eligibility at the full-text level. Of these, 731 were excluded for not meeting the inclusion criteria (Online Resource 3), leaving 90 studies for detailed analysis (Online Resource 4).

Most studies (92%) were retrospective and involved genetic or linkage analyses; only one study was prospective (Online Resource 5) [37]. None was randomized controlled trials or clinical trials. Sample sizes varied widely (4 to 5606; median 40), with NPH patient counts ranging from 1 to 447 (median 16). In 14% of studies, sample size was unclear. Only 33% reported age (range: 1 month to 73 years; median: 4.6–42.5; means: 4.8–39), and 44% reported sex, with a slight male predominance (sex ratio 1.2).

### Quality of included studies

The vast majority of included studies received good quality ratings (52/90, 58%), while 28 (31%) and 10 (11%) obtained moderate and poor quality ratings, respectively (Online Resource 6). Moderate and poor quality ratings resulted mainly from missing information regarding demographic and clinical characteristics of participants, participant recruitment, or the extensive use of statistical tests without providing sufficient evidence that the necessary assumptions of these tests were met.

Results of the Kruskal–Wallis test yielded no statistically significant differences between the three quality rating categories with respect to the number of reported SONG core (*H*(2) = 1.018; *p* = 0.601), middle tier (*H*(2) = 4.384; *p* = 0.112), and outer tier outcomes (*H*(2) = 1.009 *p* = 0.604). Similar results were obtained for clinical (*H*(2) = 0.000; *p* = 1.000) and patient-reported outcomes (*H*(2) = 1.465; *p* = 0.481). The only significant difference was observed in the reporting of surrogate parameters across study quality groups (*H*(2) = 6.197; *p* = 0.045). Post hoc pairwise comparison using Dunn’s test with Bonferroni correction revealed that studies with good quality ratings were significantly more likely to report surrogate parameters compared to those rated as poor (*Z* = − 2.489, *p* = 0.013, adjusted *p* = 0.038).

### Outcomes by domain/organ system

All included studies reported at least one clinical outcome, whereas the vast majority of studies (77/90, 85%) reported one or more surrogate parameters (Online Resource 7). Patient-reported outcome measures were only reported by 37 studies (41%). While kidney and ophthalmological outcomes were covered by most studies (97% and 62%), outcomes from cardiac (28/90, 31%), liver (41/90, 46%), mental development/neurological disease (52/90, 58%), skeletal (35/90, 39%), and respiratory domains (20/90, 22%) were reported less frequently. Outcomes from the social domain were only reported by one study (1%) (Table 2).

**Table 2:**
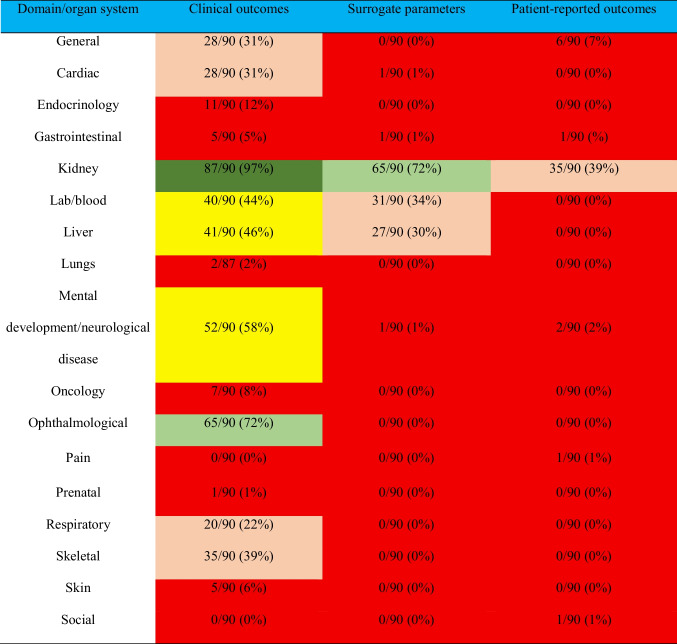
Number of studies reporting at least one outcome in different domains of clinical outcomes, surrogate parameters, and patient reported outcomes. Color code: 80–100%, dark green; 60–79%, light green; 40–59%, yellow; 20–39%, rosé; 0–19%: red

### SONG Kids outcomes

**Fig. 3:**
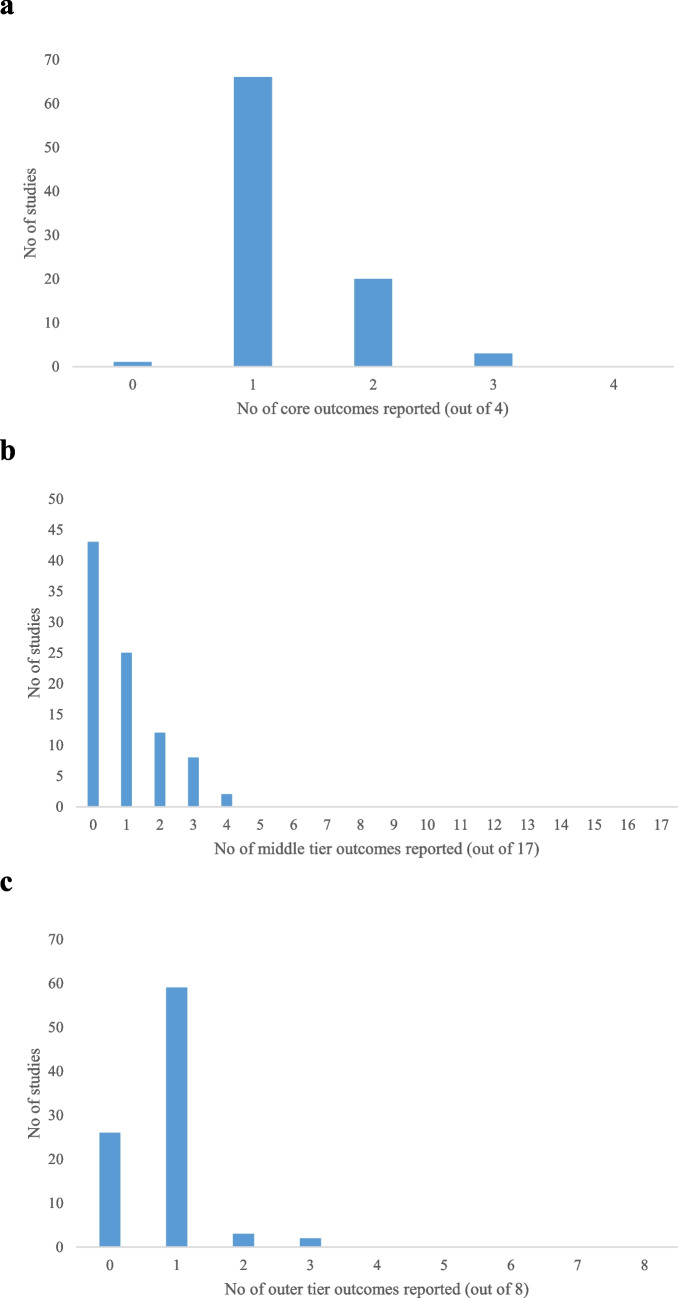
Number of SONG Kids **a** core, **b** middle tier, and **c** outer tier outcomes covered in 90 clinical studies on NPH

The majority of included studies (73%) reported only one of the four SONG Kids core outcomes, with very few addressing multiple core domains (Fig. 3). Kidney function was the most frequently reported core outcome (89/90, 99%), followed by survival (22/90, 24%). Infections and life participation were only addressed in three and one studies, respectively. A substantial number of studies (48%) did not report any of the SONG Kids middle tier outcomes, and while 24% reported at least two, none addressed more than four. The three most frequently reported middle tier outcomes were blood pressure (27/90, 30%), anemia (23/90, 26%), and growth (21/90, 23%). The majority of the middle tier outcomes, including ability to travel, academic performance anxiety, cardiovascular disease, or hospitalization, were not reported by any of the included studies. Among the patient-reported middle tier outcomes, fatigue (4/90, 4%) and pain (1/90, 1%) were the only ones to be documented.

Regarding the SONG Kids outer tier outcomes, the majority of studies (66%) reported only one, while 29% reported none at all, and only a small fraction addressed two or more. Vision was the most frequently reported outer tier outcome (63/90, 70%), followed by diabetes (3/90, 3%), itching, and gastrointestinal problems (both 2/87, 2%). Other outer tier outcomes were not reported.

### Patient-reported outcomes

Thirty-seven studies (41%) included in the review reported any patient-reported outcomes, with the majority of these focusing on polyuria/polydipsia (35/90, 39%). Other outcomes reported included enuresis (12/90, 13%), fatigue (4/90, 4%), nocturia (3/90, 3%), and pruritus (2/90, 2%).

Only two studies reported systematic collection of patient-reported outcomes. Stokman et al. employed patient interviews in conjunction with a review of medical records to procure data regarding symptoms [38], while Mehrkash et al. were the sole authors reporting the use of a validated questionnaire [39], namely the 4-Item Itch Questionnaire (4IIQ) [40]. However, the 4IIQ was not developed for patients with chronic kidney disease but was validated in a patient cohort with psoriasis and atopic dermatitis. In conclusion, none of the included studies used a disease-specific, validated instrument for the assessment of patient-reported outcomes.

## Discussion

Clinical and surrogate parameters often fail to reflect treatment benefits that matter most to those living with the disease [41]. Patients are experts of their own condition, and incorporating their feedback is essential to improve the quality of healthcare. Routine use of PROMs in clinical practice increases patients’ satisfaction with care, symptom management, quality of life, and survival rates [42–44]. Both the FDA and EMA have published strategy papers to accelerate the use of PROMs in drug development, and the FDA has developed guidelines to lead researchers through the process of PROM establishment [45–49]. While this has resulted in a significant increase in the development of PROMs overall, it has taken a little longer for this approach to gain traction in the field of rare diseases [21, 50, 51]. The focus of NPH research still lies on understanding the underlying molecular background and complex processes of the disease. The standard of care for NPH is dialysis and organ transplantation, with clinical management being limited to treatment of symptoms. Therapeutic targets and disease-specific drugs are still lacking, explaining the non-existence of prospective trials [38, 52].

The underrepresentation of PROMs in the studies included in this review is not unique to NPH but reflects a common challenge in rare disease research [53, 54]. A growing number of initiatives are working to close this gap and the European Rare Disease Research Coordination and Support Action (ERICA) PROM Repository now lists disease-specific PROMs for 128 rare diseases (count 14^th^ February 2025) [55]. Given that this represents only 1.8% of the approximately 7000 known rare diseases, there is still a significant amount of work ahead for the rare disease research community. The lack of a validated, disease-specific PROM for NPH is likely due to several NPH-specific challenges that make research in general, and PROM development in particular, significantly more difficult. NPH is an ultra-rare disease. The network for early onset cystic kidney disease (NEOCYST) registry, one of the world’s largest registries for renal ciliopathies, includes only 250 patients with NPH [13]. The genetic spectrum of NPH continues to expand, with disease-causing variants identified in more than 20 genes so far [12]. The number of affected individuals per genotype is small with a wide phenotypic variability. Another challenge associated with NPH is its tendency to cause a gradual decline in kidney function, which can often go unnoticed until kidney failure develops. The diagnosis of NPH is further complicated by the lack of distinctive symptoms and non-specific sonographic findings [56, 57], making it likely that many cases—especially among adults—remain undiagnosed due to the infrequent use of genetic testing in this population [58]. Over the past decade, clinical registry studies have played a crucial role in the clinical characterization of NPH, particularly by expanding registry data to include the systematic collection of outcomes that matter to patients. This approach has highlighted previously overlooked issues, such as concentration deficits, sleep disturbances, and enuresis and daytime incontinence. These challenges contribute to a significant psychosocial burden on both patients and their families.

While there is currently no disease-specific PROM for NPH, several validated PROMs are available for patients with chronic kidney diseases [59–61]. The SONG initiative has developed numerous outcome sets and PROMs for a range of kidney diseases [25, 26, 29, 62, 63], including the SONG Kids outcome set, which is specifically designed for children and adolescents with chronic kidney disease [24, 28]. However, our analysis revealed only a moderate overlap between the SONG Kids outcome set and the outcomes reported in the studies included in this review. This could be attributed to the fact that the symptom spectrum of NPH extends far beyond chronic kidney disease. Depending on the underlying genetic cause, patients with NPH may also exhibit reduced vision, night blindness, restricted visual fields [14], as well as liver-associated symptoms such as hepatopathy and portal hypertension [10, 16]. Additionally, neurological symptoms, therapy-resistant anemia, growth retardation, and failure to thrive are frequently observed in these patients [10, 64]. Because the SONG Kids outcome set was developed for broader pediatric CKD populations, it may miss or underrepresent clinically significant manifestations that are central to the lived experience of children with NPH. Disease-group-specific PROMs often lack the responsiveness and compatibility needed to fully capture the unique symptoms and challenges associated with a rare disease [22, 51]. In contrast, disease-specific PROMs are more likely to reflect outcomes that matter most to patients and their families, and they offer greater sensitivity to changes in health status [50, 51].

In the context of orphan drug approval, PROMs can provide critical evidence of patient benefit, especially when traditional clinical endpoints are limited or not feasible. The FDA provides a pathway for PROM qualification through its Clinical Outcome Assessment Qualification Program (https://www.fda.gov/drugs/drug-development-tool-ddt-qualification-programs/clinical-outcome-assessment-coa-qualification-program), enabling researchers to access funding opportunities that support patient-focused drug development (https://www.fda.gov/drugs/development-approval-process-drugs/cder-pilot-grant-program-standard-core-clinical-outcome-assessments-coas-and-their-related-endpoints). This program not only offers scientific guidance but also facilitates regulatory acceptance of PROMs, thereby enhancing their credibility and utility in clinical trials. The regulatory openness presents a valuable opportunity for the NPH research community to develop and validate disease-specific PROMs, which could enhance the likelihood of regulatory recognition and accelerate approval pathways for targeted therapies. These considerations highlight a significant unmet need for the development of a disease-specific set of PROMs for NPH that reflects the multisystem involvement and rare nature of this condition, which would play a crucial role in ensuring that patients receive the best possible medical care.

Within the efforts for developing PROMs in the TheRaCil network, patients and families will be actively engaged in the development of outcome assessment tools through structured focus groups and interviews to ensure the measures reflect real-world experiences and priorities. Patient advocacy groups will play a key role in reaching and recruiting a diverse group of participants, facilitating meaningful involvement across different disease presentations and age groups. These groups will also support the dissemination and promotion of the finalized tools, helping to raise awareness and encourage their adoption in both research and clinical practice. This can strengthen the overall quality and patient-centeredness of research in this rare disease area.

## Conclusion

In conclusion, although NPH is a rare disease with numerous outcomes that are clearly challenging for patients, there is a need for further efforts to capture the perspectives of patients and their families. It is essential to develop systematic disease-specific tools that effectively incorporate patient-centered outcomes into future studies.

## Supplementary Information

Below is the link to the electronic supplementary material.

Graphical abstract (PPTX 217 KB)

(PDF 2.49 MB)
