# Supplementary material for: Systematic review of outcomes reported in clinical research on nephronophthisis: how do they align with SONG Kids priorities?
Source: Pediatr Nephrol. 2025 Aug 20;41(2):339–51. doi: 10.1007/s00467-025-06912-0 (PMC12727818; doi:10.1007/s00467-025-06912-0)
Supplement: Supplementary file 1 — Graphical abstract (PPTX 217 KB) [file 467_2025_6912_MOESM1_ESM.pptx]

## Slide 1
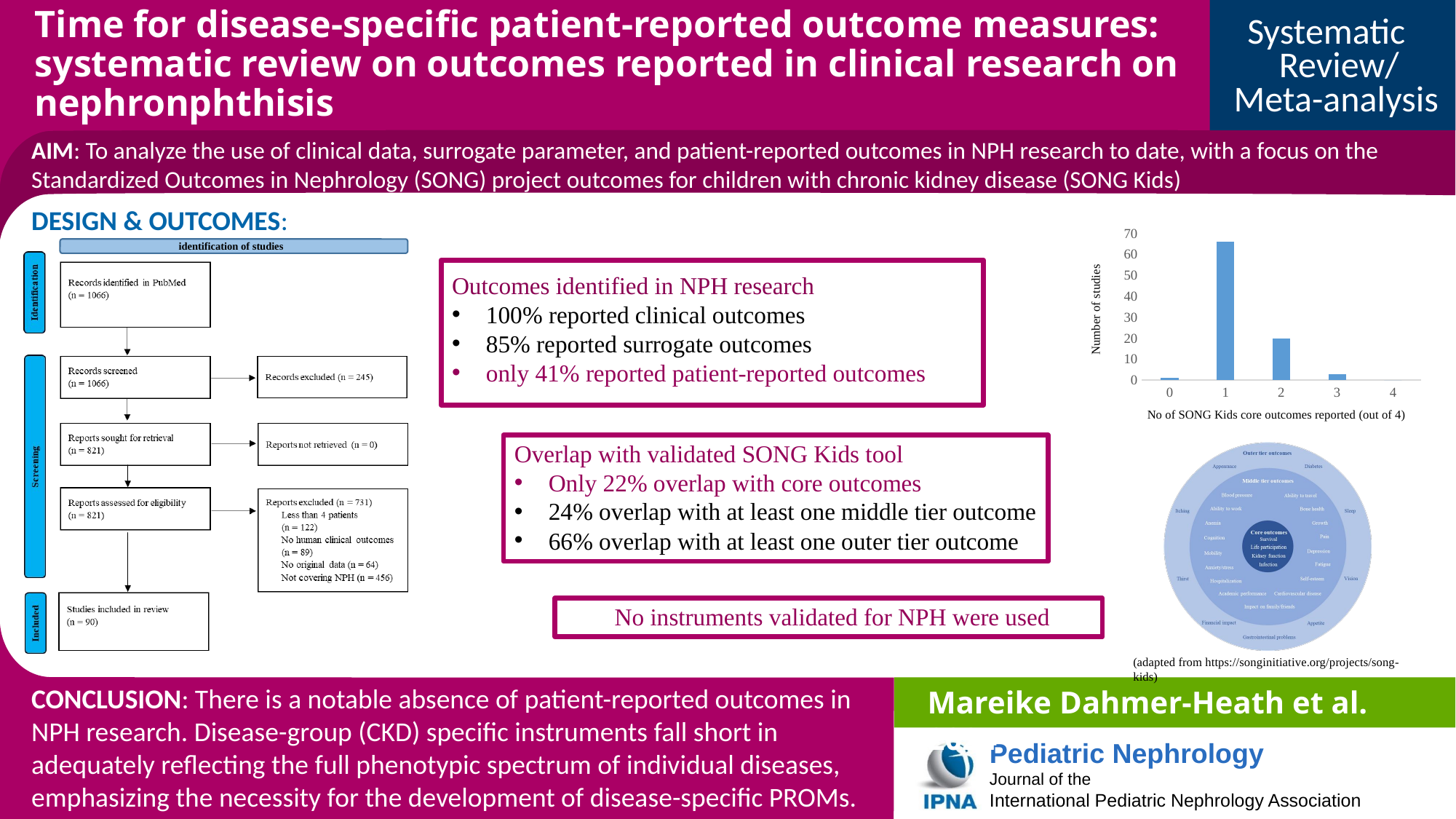

Time for disease-specific patient-reported outcome measures: systematic review on outcomes reported in clinical research on nephronphthisis
AIM: To analyze the use of clinical data, surrogate parameter, and patient-reported outcomes in NPH research to date, with a focus on the Standardized Outcomes in Nephrology (SONG) project outcomes for children with chronic kidney disease (SONG Kids)
DESIGN & OUTCOMES:
### Chart
| Category | |
|---|---|
| 0 | 1.0 |
| 1 | 66.0 |
| 2 | 20.0 |
| 3 | 3.0 |
| 4 | 0.0 |identification of studies
Outcomes identified in NPH research
100% reported clinical outcomes
85% reported surrogate outcomes
only 41% reported patient-reported outcomes
Number of studies
No of SONG Kids core outcomes reported (out of 4)
Overlap with validated SONG Kids tool
Only 22% overlap with core outcomes
24% overlap with at least one middle tier outcome
66% overlap with at least one outer tier outcome
No instruments validated for NPH were used
(adapted from https://songinitiative.org/projects/song-kids)
CONCLUSION: There is a notable absence of patient-reported outcomes in NPH research. Disease-group (CKD) specific instruments fall short in adequately reflecting the full phenotypic spectrum of individual diseases, emphasizing the necessity for the development of disease-specific PROMs.
Mareike Dahmer-Heath et al. 2024
