# Supplementary material for: Systematic review of outcomes reported in clinical research on nephronophthisis: how do they align with SONG Kids priorities?
Source: Pediatr Nephrol. 2025 Aug 20;41(2):339–51. doi: 10.1007/s00467-025-06912-0 (PMC12727818; doi:10.1007/s00467-025-06912-0)
Supplement: Supplementary file 2 — (PDF 2.49 MB) [file 467_2025_6912_MOESM2_ESM.pdf]

Online Resources

Time for disease-specific patient-reported outcome measures: Systematic review on outcomes reported in clinical research on nephronophthisis.
Pediatric Nephrology

Mareike Dahmer-Heath (MD-H)[1*], Sven Optenhövel (SO)[1,2*], Tanja Hechler (TH)[2], Martin Konrad (MK)[1], Jens König (JK)[1] *: authors contributed equally
1 Department of General Pediatrics, University Children's Hospital Münster, Münster, Germany
2 Department of Clinical Psychology and Psychotherapy for Children and Adolescents, University of Münster, Münster, Germany
Correspondence: Jens Christian König, Department of General Pediatrics, Universitätsklinikum Münster, Waldeyerstraße 22, 48149 Münster, Germany. E-mail: jens.koenig@ukmuenster.de

Online Resource 1: PRISMA Checklist

Section and Topic	Item #	Checklist item	Location where item is reported
**TITLE**			
Title	1	Identify the report as a systematic review.	1
**ABSTRACT**			
Abstract	2	See the PRISMA 2020 for Abstracts checklist.	
**INTRODUCTION**			
Rationale	3	Describe the rationale for the review in the context of existing knowledge.	
Objectives	4	Provide an explicit statement of the objective(s) or question(s) the review addresses.	
**METHODS**			
Eligibility criteria	5	Specify the inclusion and exclusion criteria for the review and how studies were grouped for the syntheses.	4
Information sources	6	Specify all databases, registers, websites, organisations, reference lists and other sources searched or consulted to identify studies. Specify the date when each source was last searched or consulted.	3
Search strategy	7	Present the full search strategies for all databases, registers and websites, including any filters and limits used.	3-4
Selection process	8	Specify the methods used to decide whether a study met the inclusion criteria of the review, including how many reviewers screened each record and each report retrieved, whether they worked independently, and if applicable, details of automation tools used in the process.	4
Data collection process	9	Specify the methods used to collect data from reports, including how many reviewers collected data from each report, whether they worked independently, any processes for obtaining or confirming data from study investigators, and if applicable, details of automation tools used in the process.	4

Section and Topic	Item #	Checklist item	Location where item is reported
Data items	10a	List and define all outcomes for which data were sought. Specify whether all results that were compatible with each outcome domain in each study were sought (e.g. for all measures, time points, analyses), and if not, the methods used to decide which results to collect.	n.a.
	10b	List and define all other variables for which data were sought (e.g. participant and intervention characteristics, funding sources). Describe any assumptions made about any missing or unclear information.	4
Study risk of bias assessment	11	Specify the methods used to assess risk of bias in the included studies, including details of the tool(s) used, how many reviewers assessed each study and whether they worked independently, and if applicable, details of automation tools used in the process.	5
Effect measures	12	Specify for each outcome the effect measure(s) (e.g. risk ratio, mean difference) used in the synthesis or presentation of results.	n.a.
Synthesis methods	13a	Describe the processes used to decide which studies were eligible for each synthesis (e.g. tabulating the study intervention characteristics and comparing against the planned groups for each synthesis (item #5)).	n.a.
	13b	Describe any methods required to prepare the data for presentation or synthesis, such as handling of missing summary statistics, or data conversions.	n.a.
	13c	Describe any methods used to tabulate or visually display results of individual studies and syntheses.	4-5
	13d	Describe any methods used to synthesize results and provide a rationale for the choice(s). If meta-analysis was performed, describe the model(s), method(s) to identify the presence and extent of statistical heterogeneity, and software package(s) used.	5
	13e	Describe any methods used to explore possible causes of heterogeneity among study results (e.g. subgroup analysis, meta-regression).	n.a.
	13f	Describe any sensitivity analyses conducted to assess robustness of the synthesized results.	n.a.
Reporting bias assessment	14	Describe any methods used to assess risk of bias due to missing results in a synthesis (arising from reporting biases).	n.a.
Certainty assessment	15	Describe any methods used to assess certainty (or confidence) in the body of evidence for an outcome.	5
**RESULTS**			
Study selection	16a	Describe the results of the search and selection process, from the number of records identified in the search to the number of studies included in the review, ideally using a flow diagram.	5-6
	16b	Cite studies that might appear to meet the inclusion criteria, but which were excluded, and explain why they were excluded.	
Study characteristics	17	Cite each included study and present its characteristics.	20-22
Risk of bias in studies	18	Present assessments of risk of bias for each included study.	23
Results of	19	For all outcomes, present, for each study: (a) summary statistics for each group (where appropriate) and (b) an effect estimate and its precision	n.a.

Section and Topic	Item #	Checklist item	Location where item is reported
individual studies		(e.g. confidence/credible interval), ideally using structured tables or plots.	
Results of syntheses	20a	For each synthesis, briefly summarise the characteristics and risk of bias among contributing studies.	
	20b	Present results of all statistical syntheses conducted. If meta-analysis was done, present for each the summary estimate and its precision (e.g. confidence/credible interval) and measures of statistical heterogeneity. If comparing groups, describe the direction of the effect.	
	20c	Present results of all investigations of possible causes of heterogeneity among study results.	n.a.
	20d	Present results of all sensitivity analyses conducted to assess the robustness of the synthesized results.	n.a.
Reporting biases	21	Present assessments of risk of bias due to missing results (arising from reporting biases) for each synthesis assessed.	n.a.
Certainty of evidence	22	Present assessments of certainty (or confidence) in the body of evidence for each outcome assessed.	
**DISCUSSION**			
Discussion	23a	Provide a general interpretation of the results in the context of other evidence.	
	23b	Discuss any limitations of the evidence included in the review.	
	23c	Discuss any limitations of the review processes used.	
	23d	Discuss implications of the results for practice, policy, and future research.	
**OTHER INFORMATION**			
Registration and protocol	24a	Provide registration information for the review, including register name and registration number, or state that the review was not registered.	
	24b	Indicate where the review protocol can be accessed, or state that a protocol was not prepared.	
	24c	Describe and explain any amendments to information provided at registration or in the protocol.	
Support	25	Describe sources of financial or non-financial support for the review, and the role of the funders or sponsors in the review.	
Competing interests	26	Declare any competing interests of review authors.	
Availability of data, code and other materials	27	Report which of the following are publicly available and where they can be found: template data collection forms; data extracted from included studies; data used for all analyses; analytic code; any other materials used in the review.	

From: Page MJ, McKenzie JE, Bossuyt PM, Boutron I, Hoffmann TC, Mulrow CD, et al. The PRISMA 2020 statement: an updated guideline for reporting systematic reviews. BMJ 2021;372:n71. doi: 10.1136/bmj.n71

Online Resource 2: Normality testing

		Kolmogorov-Smirnov[a]			Shapiro-Wilk		
	Study quality	Statistic	df	Significance	Statistic	df	Significance
SONG Core outome	good	,455	52	<,001	,574	52	<,001
	moderate	,436	28	<,001	,612	28	<,001
	poor	,370	10	<,001	,752	10	,004
SONG Middle tier outcomes	good	,231	52	<,001	,835	50	<,001
	moderate	,331	28	<,001	,717	27	<,001
	poor	,360	10	<,001	,731	10	,002
SONG Outer tier outcomes	good	,332	52	<,001	,739	52	<,001
	moderate	,365	28	<,001	,637	28	<,001
	poor	,381	10	<,001	,640	10	<,001
Clinical outcomes	good	.	50	.	.	50	.
	moderate	.	27	.	.	27	.
	poor	.	10	.	.	10	.
Surrogate outcomes	good	,531	52	<,001	,336	52	<,001
	moderate	,513	28	<,001	,419	28	<,001
	poor	,381	10	<,001	,640	10	<,001
Patient reported outcomes	good	,359	52	<,001	,635	52	<,001
	moderate	,429	28	<,001	,591	28	<,001
	poor	,381	10	<,001	,640	10	<,001

a. Lilliefors' significance correction

Online Resource 3: List of excluded studies

<u>*Less than 4 patients*</u>

Abdelwahed M, Maaloul I, Benoit V, Hilbert P, Hachicha M, Kamoun H, et al. Copy-number variation of the NPHP1 gene in patients with juvenile Nephronophthisis. Acta clinica Belgica. 2021;76(1):16-24. https://doi.org/10.1080/17843286.2019.1655231.

Abo El Fotoh WMM, Al-Fiky AF. A Compound Heterozygous Mutation in the Ciliary Gene TTC21B Causes Nephronophthisis Type 12. Journal of pediatric genetics. 2020;9(3):198-202. https://doi.org/10.1055/s-0039-1700804.

Ahmed J, Ali US. Joubert syndrome with nephronophthisis in neurofibromatosis type 1. Saudi J Kidney Dis Transpl. 2011;22(4):788-91. https://journals.lww.com/sjkd/fulltext/2011/22040/joubert_syndrome_with_nephronophthisis_in.26.aspx.

Ajiboye O, Vengoechea JE, Gupta R, Lomashvili K. Autosomal Recessive Adolescent Syndromic Nephronophthisis Caused by a Novel Compound Heterozygous Pathogenic Variant. Am J Case Rep. 2023;24:e941413. https://doi.org/10.12659/ajcr.941413.

Akira M, Suzuki H, Ikeda A, Iwasaki M, Honda D, Takahara H, et al. Atypical histological abnormalities in an adult patient with nephronophthisis harboring NPHP1 deletion: a case report. BMC Nephrol. 2021;22(1):261. https://doi.org/10.1186/s12882-021-02466-z.

Al Alawi I, Powell L, Rice SJ, Al Riyami MS, Al-Riyami M, Al Salmi I, et al. Case Report: A Novel In-Frame Deletion of GLIS2 Leading to Nephronophthisis and Early Onset Kidney Failure. Frontiers in genetics. 2021;12:791495. https://doi.org/10.3389/fgene.2021.791495.

Al-Eisa AA, Samhan M, Naseef M. End-stage renal disease in Kuwaiti children: an 8-year experience. Transplant Proc. 2004;36(6):1788-91. https://doi.org/10.1016/j.transproceed.2004.07.024.

Alazami AM, Alshammari MJ, Baig M, Salih MA, Hassan HH, Alkuraya FS. NPHP4 mutation is linked to cerebello-oculo-renal syndrome and male infertility. Clinical genetics. 2014;85(4):371-5. https://doi.org/10.1111/cge.12160.

Albaramki J, Akl K, Hamed R, Wahbeh A. A family with five siblings affected with nephronophthisis. Saudi J Kidney Dis Transpl. 2014;25(3):630-3. https://doi.org/10.4103/1319-2442.132218.

AlFadhel M, AlAmir A. Senior-Loken syndrome in a Saudi child. Saudi J Kidney Dis Transpl. 2008;19(3):443-5. https://journals.lww.com/sjkd/Fulltext/2008/19030/Senior_Loken_Syndrome_in_a_Saudi_Child.18.aspx

Alizadeh R, Jamshidi S, Keramatipour M, Moeinian P, Hosseini R, Otukesh H, et al. Whole Exome Sequencing Reveals a XPNPEP3 Novel Mutation Causing Nephronophthisis in a Pediatric Patient. Iranian biomedical journal. 2020;24(6):405-8. https://doi.org/10.29252/ibj.24.6.400.

Alsing A, Christensen C. Atypical macular coloboma (dysplasia) associated with familial juvenile nephronophthisis and skeletal abnormality. Ophthalmic paediatrics and genetics. 1988;9(3):149-55. https://doi.org/10.3109/13816818809031491.

AlZabali SM, AlAnazi A, Rahim KA, Faqeehi HY. Clinical improvement of encapsulating peritoneal sclerosis after challenging course and 6 months of total parenteral nutrition in child with nephronophthisis: a case report. Journal of medical case reports. 2021;15(1):366. https://doi.org/10.1186/s13256-021-02905-3.

Amano K, Toyoda H, Nishikawa K, Murata T, Hirayama M. Case Report: Effects of Secondary Hyperparathyroidism Treatment on Improvement of Juvenile Nephronophthisis-Induced Pancytopenia and Myelofibrosis. Front Pediatr. 2021;9:550158. https://doi.org/10.3389/fped.2021.550158.

Assadi F. Lack of NPHP2 mutations in a newborn infant with Joubert syndrome-related disorder presenting as end-stage renal disease. Pediatr Nephrol. 2007;22(5):750-2. https://doi.org/10.1007/s00467-006-0412-z.

Awazu M, Yamada M, Asada N, Hashiguchi A, Kosaki K, Matsumura K. A girl with a mutation of the ciliary gene CC2D2A presenting with FSGS and nephronophthisis. CEN case reports. 2022;11(1):116-9. https://doi.org/10.1007/s13730-021-00640-8.

Azabdaftari A, Sczakiel HL, Danyel M, Kohlmaier B, Mache CJ, Stalke A, et al. Biallelic known and novel DCDC2 variants in cholestatic liver disease: Phenotype-genotype observations in four children. Liver Int. 2023;43(5):1089-95. https://doi.org/10.1111/liv.15563.

Bagga A, Vasudev A, Kabra SK, Mukhopadhyay S, Bhuyan UN, Srivastava R. Nephronophthisis with bronchiectasis. Child nephrology and urology. 1990;10(4):211-3. https://doi.org/10.1097/MOP.0000000000000194.

Balfe JW. Transplantation of a 14-year-old girl with nephronophthisis. Pediatr Nephrol. 1997;11(1):132.

Balgradean M, Cinteza E, Ferechide D. Post renal transplant type 2 diabetes mellitus in a case of familial juvenile nephrophthisis. Maedica. 2013;8(1):26-9. https://pmc.ncbi.nlm.nih.gov/articles/PMC3749756/.

Balkaran B, Ramcharan J, Rao AV, Ramanjaneyulu M, Roberts LA. Two Afro-Trinidadian siblings with end-stage renal disease. Annals of tropical paediatrics. 1998;18(3):249-52. https://doi.org/10.1080/02724936.1998.11747955.

Ben-Yosef T, Asia Batsir N, Ali Nasser T, Ehrenberg M. Retinal dystrophy as part of TTC21B-associated ciliopathy. Ophthalmic genetics. 2021;42(3):329-33. https://doi.org/10.1080/13816810.2021.1888131.

Betz R, Rensing C, Otto E, Mincheva A, Zehnder D, Lichter P, et al. Children with ocular motor apraxia type Cogan carry deletions in the gene (NPHP1) for juvenile nephronophthisis. J Pediatr. 2000;136(6):828-31. https://doi.org/10.1016/S0022-3476(00)01001-5.

Bhimma R, Jembere E, Hariparshad S. Case report of a child with nephronophthisis from South Africa. BMC pediatrics. 2024;24(1):431. https://doi.org/10.1186/s12887-024-04872-2.

Bianchi C, Barera G, Picciotti M, Barbiano di Belgioioso G, Bellini F. Juvenile nephronophthisis associated with new skeletal abnormalities, tapetoretinal degeneration and liver fibrosis. Helvetica paediatrica acta. 1989;43(5-6):449-55. https://doi.org/10.1136/adc.51.10.799.

Blowey DL, Alon U, Hellerstein S, Warady BA, Wood BP. Radiological cases of the month. Juvenile nephronophthisis. American journal of diseases of children (1960). 1993;147(10):1117-8. https://doi.org/10.1001/archpedi.1993.02160340103023.

Brancati F, Camerota L, Colao E, Vega-Warner V, Zhao X, Zhang R, et al. Biallelic variants in the ciliary gene TMEM67 cause RHYNS syndrome. European journal of human genetics : EJHG. 2018;26(9):1266-71. https://doi.org/10.1038/s41431-018-0183-6.

Brndiarova M, Antonyova M, Dedinska I, Havlicekova Z, Jesenak M. Nephronophthisis type I, left ventricular non-compaction cardiomyopathy and reduced cilia motility-atypical manifestations of one disease. Journal of nephrology. 2020;33(1):183-6. https://doi.org/10.1007/s40620-019-00651-w.

Brndiarova M, Mraz M, Kolkova Z, Cisarik F, Banovcin P. Sensenbrenner Syndrome Presenting with Severe Anorexia, Failure to Thrive, Chronic Kidney Disease and Angel-Shaped Middle Phalanges in Two Siblings. Molecular syndromology. 2021;12(4):263-7. https://doi.org/10.1159/000515645.

Buke B, Canverenler E, İpek G, Canverenler S, Akkaya H. Diagnosis of Joubert syndrome via ultrasonography. Journal of medical ultrasonics (2001). 2017;44(2):197-202. https://doi.org/10.1007/s10396-016-0751-8.

Büscher R, Büscher AK, Cetiner M, Treckmann JW, Paul A, Vester U, et al. Combined liver and kidney transplantation and kidney after liver transplantation in children: Indication, postoperative outcome, and long-term results. Pediatr Transplant. 2015;19(8):858-65. https://doi.org/10.1111/petr.12595.

Chen H, Lin H, Yue Z, Wang H, Yang J, Sun L. Two Chinese nephronophthisis pedigrees harbored a compound heterozygous deletion with a point mutation in NPHP1. International journal of molecular epidemiology and genetics. 2019;10(4):53-8. https://pmc.ncbi.nlm.nih.gov/articles/PMC6737400/.

Chen L, Uchida H, Komine R, Kodama T, Nakao T, Okada N, et al. The role of liver transplantation in COACH syndrome (Joubert syndrome with congenital hepatic fibrosis): A review of the literature. Pediatr Transplant. 2024;28(1):e14640. https://doi.org/10.1111/petr.14640.

Chen WLJ, Fung KFK, Chan EY. Nephronophthisis-associated ciliopathy with brachydactyly, medullary cysts, and chronic kidney disease. Kidney Int. 2024;106(4):759. https://doi.org/10.1016/j.kint.2024.04.022.

Deacon BS, Lowery RS, Phillips PH, Schaefer GB. Congenital ocular motor apraxia, the NPHP1 gene, and surveillance for nephronophthisis. Journal of AAPOS : the official publication of the American Association for Pediatric Ophthalmology and Strabismus. 2013;17(3):332-3. https://doi.org/10.1016/j.jaapos.2013.02.003.

Derakhshan N, Derakhshan D, Torabinejad S, Derakhshan A. Nephritic-nephrotic syndrome as a presentation of BK virus infection. Saudi J Kidney Dis Transpl. 2011;22(1):123-5. https://www.researchgate.net/publication/49719659_Nephritic-nephrotic_syndrome_as_a_presentation_of_BK_virus_infection.

Eisenstein B, Davidovitz M, Garty BZ, Shmueli D, Ussim A, Stark H. Severe tubular resistance to aldosterone in a child with familial juvenile nephronophthisis. Pediatr Nephrol. 1992;6(1):57-9. https://doi.org/10.1007/bf00856835.

Elzouki A, Mirza K. Clinical quiz. Familial juvenile nephronophthisis. Pediatr Nephrol. 1994;8(4):525-6. https://doi.org/10.1007/bf00856557.

Elzouki AY, al-Suhaibani H, Mirza K, al-Sowailem AM. Thin-section computed tomography scans detect medullary cysts in patients believed to have juvenile nephronophthisis. Am J Kidney Dis. 1996;27(2):216-9. https://doi.org/10.1016/s0272-6386(96)90543-0.

Fang B, Guo J, Hao C, Guo R, Qian S, Li W, et al. Whole-exome sequencing identifies a novel compound heterozygous mutation of ANKS6 gene in a Chinese nephronophthisis patient. Clinica chimica acta; international journal of clinical chemistry. 2020;501:131-5. https://doi.org/10.1016/j.cca.2019.10.030.

Fehrenbach H, Decker C, Eisenberger T, Frank V, Hampel T, Walden U, et al. Mutations in WDR19 encoding the intraflagellar transport component IFT144 cause a broad spectrum of ciliopathies. Pediatr Nephrol. 2014;29(8):1451-6. https://doi.org/10.1007/s00467-014-2762-2.

Forbes TA, Howden SE, Lawlor K, Phipson B, Maksimovic J, Hale L, et al. Patient-iPSC-Derived Kidney Organoids Show Functional Validation of a Ciliopathic Renal Phenotype and Reveal Underlying

Pathogenetic Mechanisms. Am J Hum Genet. 2018;102(5):816-31. https://doi.org/10.1016/j.ajhg.2018.03.014.

Gagnadoux MF, Bacri JL, Broyer M, Habib R. Infantile chronic tubulo-interstitial nephritis with cortical microcysts: variant of nephronophthisis or new disease entity? Pediatr Nephrol. 1989;3(1):50-5. https://doi.org/10.1007/bf00859626.

Gillesse E, Wade A, Parboosingh JS, Au PYB, Bernier FP, Lamont RE, et al. Genome sequencing identifies biallelic variants in SCLT1 in a patient with syndromic nephronophthisis: Reflections on the SCLT1-related ciliopathy spectrum. American journal of medical genetics Part A. 2024;194(11):e63789. https://doi.org/10.1002/ajmg.a.63789.

Giridhar S, Padmaraj R, Senguttuvan P. Twins with senior-Loken syndrome. Indian journal of pediatrics. 2006;73(11):1041-3. https://doi.org/10.1007/bf02758316.

Goswami M, Rajwar AS, Verma M. Orocraniofacial findings of a Pediatric Patient with Joubert Syndrome. International journal of clinical pediatric dentistry. 2016;9(4):379-83. https://doi.org/10.5005/jp-journals-10005-1394.

Gunay-Aygun M, Parisi MA, Doherty D, Tuchman M, Tsilou E, Kleiner DE, et al. MKS3-related ciliopathy with features of autosomal recessive polycystic kidney disease, nephronophthisis, and Joubert Syndrome. J Pediatr. 2009;155(3):386-92.e1. https://doi.org/10.1016/j.jpeds.2009.03.045.

Hafeez F, Rasool F, Ahmad TM. Nephronophthisis: a variant. Journal of the College of Physicians and Surgeons--Pakistan : JCPSP. 2005;15(6):368-70. https://www.researchgate.net/publication/7816846_Nephronophthisis_A_variant.

Haghighi A, Savaj S, Haghighi-Kakhki H, Benoit V, Grisart B, Dahan K. Identification of an NPHP1 deletion causing adult form of nephronophthisis. Irish journal of medical science. 2016;185(3):589-95. https://doi.org/10.1007/s11845-015-1312-7.

Hibino S, Morisada N, Takeda A, Tanaka K, Nozu K, Yamakawa S, et al. Medullary Cystic Kidney Disease and Focal Segmental Glomerulosclerosis Caused by a Compound Heterozygous Mutation in TTC21B. Internal medicine (Tokyo, Japan). 2020;59(14):1735-8. https://doi.org/10.2169/internalmedicine.4266-19.

Hirai Y, Mizumoto A, Mitsumoto K, Uzu T. Senior-Løken syndrome misdiagnosed as nephrosclerosis related to hypertensive disorders of pregnancy. BMJ case reports. 2020;13(10). https://doi.org/10.1136/bcr-2020-236137.

Hirano D, Fujinaga S, Ohtomo Y, Nishizaki N, Hara S, Murakami H, et al. Nephronophthisis cannot be detected by urinary screening program. Clinical pediatrics. 2013;52(8):759-61. https://doi.org/10.1177/0009922812441390.

Hoefele J, Otto E, Felten H, Kühn K, Bley TA, Zäuner I, et al. Clinical and histological presentation of 3 siblings with mutations in the NPHP4 gene. Am J Kidney Dis. 2004;43(2):358-64. https://doi.org/10.1053/j.ajkd.2003.10.023.

Huppke P, Wegener E, Böhrer-Rabel H, Bolz HJ, Zoll B, Gärtner J, et al. Tectonic gene mutations in patients with Joubert syndrome. European journal of human genetics : EJHG. 2015;23(5):616-20. https://doi.org/10.1038/ejhg.2014.160.

Hurd TW, Otto EA, Mishima E, Gee HY, Inoue H, Inazu M, et al. Mutation of the Mg2+ transporter SLC41A1 results in a nephronophthisis-like phenotype. Journal of the American Society of Nephrology : JASN. 2013;24(6):967-77. https://doi.org/10.1681/asn.2012101034.

Ijaz A, Alfadhli F, Alharbi A, Khan YN, Alhawas YK, Hashmi JA, et al. NPHP3 splice acceptor site variant is associated with infantile nephronophthisis and asphyxiating thoracic dystrophy; A rare combination. European journal of medical genetics. 2022;65(10):104578. https://doi.org/10.1016/j.ejmg.2022.104578.

Irie R, Nakazawa A, Sakamoto S, Takeda M, Yanagi Y, Shimizu S, et al. Living donor liver transplantation for congenital hepatic fibrosis in children. Pathology international. 2020;70(6):348-54. https://doi.org/10.1111/pin.12917.

Isac R, Costa R, Lăzureanu DC, Olariu CI, Muntean AM, Aldea CO, et al. Lymphoproliferative disorder in a twin female teenager post kidney transplantation. Romanian journal of morphology and embryology = Revue roumaine de morphologie et embryologie. 2017;58(3):1041-5. https://www.rjme.ro/RJME/resources/files/58031710411045.pdf.

Kanazawa H, Fukuda A, Sato M, Ishimori S, Sasaki K, Uchida H, et al. Successful resumption of peritoneal dialysis following living donor liver transplantation in children with end-stage renal disease. Pediatr Transplant. 2017;21(3). https://doi.org/10.1111/petr.12897.

Kaplan BS, Milner LS, Jequier S, Kaplan P, de Chadarevian JP. Autosomal dominant inheritance of small kidneys. American journal of medical genetics. 1989;32(1):120-6. https://doi.org/10.1002/ajmg.1320320126.

Kaur A, Dhir SK, Goyal G, Mittal N, Goyal RK. Senior Loken Syndrome. Journal of clinical and diagnostic research : JCDR. 2016;10(11):Sd03-sd4. https://doi.org/10.7860/jcdr/2016/21832.8816.

Kaynar K, Kayıpmaz S, Çebi AH, Hüseynova Ş. Having Multiple Renal Cysts in a Young Adult is not Always a Sign of Polycystic Kidney Disease. Balkan journal of medical genetics : BJMG. 2021;24(2):83-7. https://doi.org/10.2478/bjmg-2021-0016.

Keenswijk W, Walle JV. A 4-year-old boy presenting with persistent urinary incontinence: Questions. Pediatr Nephrol. 2017;32(5):767-8. https://doi.org/10.1007/s00467-016-3441-2.

Keyser MN, Huang M, Newton K, Benador N, Beauchamp-Walters J, Bird LM. A unique pancreatic phenotype in a child with a WDR19-related ciliopathy: A case report and literature review of pancreatic involvement in ciliopathies. American journal of medical genetics Part A. 2022;188(7):2242-5. https://doi.org/10.1002/ajmg.a.62746.

Kinoshita H, Fujimoto S, Yokota N, Ochiai H, Hisanaga S, Hara S, et al. An isolated case of nephronophthisis: medullary cystic disease without typical onset. Internal medicine (Tokyo, Japan). 1998;37(1):83-5. https://doi.org/10.2169/internalmedicine.37.83.

Komatsuda A, Masai R, Wakui H, Iwamoto K, Aiba N, Ohtani H, et al. Analysis of the NPHP genes in two Japanese patients with suspected sporadic juvenile or adolescent nephronophthisis. Clinical nephrology. 2006;65(5):364-9. https://doi.org/10.5414/cnp65364.

Kravarusic D, Sigalet DL, Hamiwka LA, Midgley JP, Wade AW, Grisaru S. Persistent post-transplant polyuria managed by bilateral native-kidney laparoscopic nephrectomy. Pediatr Nephrol. 2006;21(6):880-2. https://doi.org/10.1007/s00467-006-0085-7.

Kulkarni S, Abro B, Duque Lasio ML, Stoll J, Grange DK, He M. Clinical and Pathological Features of a Newborn With Compound Heterozygous ANKS6 Variants. Pediatric and developmental pathology : the official journal of the Society for Pediatric Pathology and the Paediatric Pathology Society. 2020;23(3):235-9. https://doi.org/10.1177/1093526619881541.

Lacquaniti A, Chirico V, Donato V, Briuglia S, Cernaro V, Gallizzi R, et al. NGAL as an early biomarker of kidney disease in Joubert syndrome: three brothers compared. Ren Fail. 2012;34(4):495-8. https://doi.org/10.3109/0886022x.2011.649677.

Larrue R, Chamley P, Bardyn T, Lionet A, Gnemmi V, Cauffiez C, et al. Diagnostic utility of whole-genome sequencing for nephronophthisis. NPJ genomic medicine. 2020;5:38. https://doi.org/10.1038/s41525-020-00147-8.

Li L, Liu C, Tian M, Li G, Li J. Novel compound heterozygous WDR35 variants in a Chinese patient associated with cranioectodermal dysplasia and ectopic testis: a case report and review of the literature. BMC pediatrics. 2023;23(1):407. https://doi.org/10.1186/s12887-023-04110-1.

Li Y, Dai L, Xu H, Huang J, Zhang J, Mei Z, et al. Clinical report and genetic analysis of rare premature infant nephronophthisis caused by biallelic TTC21B variants. Molecular genetics & genomic medicine. 2024;12(3):e2399. https://doi.org/10.1002/mgg3.2399.

Liu CH, Li LJ, Tian M, Cao GH, Zhang SF, Li JT. Two rare copy number variants involving loss of NPHP1, MALL, and MTLN genes contribute to nephronophthisis-induced nephropathy progression in a family: A case report. Nigerian journal of clinical practice. 2023;26(4):524-7. https://doi.org/10.4103/njcp.njcp_775_22.

Liu K, Chen R, Wang X, Gong Y, Shi J, Gu B, et al. Biallelic ANKS6 null variants cause notable extrarenal phenotypes in a nephronophthisis patient and lead to hepatobiliary abnormalities by YAP1 deficiency. Clinical genetics. 2023;104(6):625-36. https://doi.org/10.1111/cge.14412.

Liu Y, Qiu T, Chen Z, Ma X, Wang T, Zhang Y, et al. A case report of two Chinese monozygotic twins with NPHP1 gene-associated nephronophthisis undergoing kidney transplantation from a related living-donor. Transpl Immunol. 2023;78:101828. https://doi.org/10.1016/j.trim.2023.101828.

Matsubara K, Suzuki K, Lin YW, Yamamoto T, Ohta S. Familial juvenile nephronophthisis in two siblings--histological findings at an early stage. Acta paediatrica Japonica : Overseas edition. 1991;33(4):482-7. https://doi.org/10.1111/j.1442-200x.1991.tb02575.x.

Matsushita HB, Hiraide T, Hayakawa K, Okano S, Nakashima M, Saitsu H, et al. Compound heterozygous ADAMTS9 variants in Joubert syndrome-related disorders without renal manifestation. Brain & development. 2022;44(2):161-5. https://doi.org/10.1016/j.braindev.2021.10.004.

Mehawej C, Chouery E, Ghabril R, Tokajian S, Megarbane A. NEK8-Associated Nephropathies: Do Autosomal Dominant Forms Exist? Nephron. 2022:1-5. https://doi.org/10.1159/000526841.

Miri Karam Z, Gohari AK, Khabaz MJR, Yari A, Meybodi SME, Attari R, et al. Identification of a Novel Deletion Variant (c.2999_3005delTGTGTGT/p.Asn1000SerfsTer4) in NPHP4 Associated With Nephronophthisis-4. J Clin Lab Anal. 2024;38(11-12):e25077. https://doi.org/10.1002/jcla.25077.

Moalem S, Keating S, Shannon P, Thompson M, Millar K, Nykamp K, et al. Broadening the ciliopathy spectrum: motile cilia dyskinesia, and nephronophthisis associated with a previously unreported homozygous mutation in the INVS/NPHP2 gene. American journal of medical genetics Part A. 2013;161a(7):1792-6. https://doi.org/10.1002/ajmg.a.36036.

Mortellaro C, Bello L, Pucci A, Lucchina AG, Migliario M. Saldino-Mainzer syndrome: nephronophthisis, retinitis pigmentosa, and cone-shaped epiphyses. The Journal of craniofacial surgery. 2010;21(5):1554-6. https://doi.org/10.1097/SCS.0b013e3181ec69bb.

Moudgil A, Bagga A, Kamil ES, Rimoin DL, Lachman RS, Cohen AH, et al. Nephronophthisis associated with Ellis-van Creveld syndrome. Pediatr Nephrol. 1998;12(1):20-2. https://doi.org/10.1007/s004670050395.

Ning K, Song E, Sendayen BE, Prosseda PP, Chang KC, Ghaffarieh A, et al. Defective INPP5E distribution in NPHP1-related Senior-Loken syndrome. Molecular genetics & genomic medicine. 2021;9(1):e1566. https://doi.org/10.1002/mgg3.1566.

O'Toole JF, Otto EA, Frishberg Y, Hildebrandt F. Retinitis pigmentosa and renal failure in a patient with mutations in INVS. Nephrol Dial Transplant. 2006;21(7):1989-91. https://doi.org/10.1093/ndt/gfl088.

Okada M, Sugimoto K, Shimada Y, Fujita S, Yanagida H, Yagi K, et al. Association of INVS (NPHP2) mutation in an adolescent exhibiting nephronophthisis (NPH) and complete situs inversus. Clinical nephrology. 2008;69(2):135-41. https://doi.org/10.5414/cnp69135.

Oki Y, Katsuma A, Okabe M, Watanabe M, Sagasaki M, Takahashi D, et al. Different Clinical Courses of Nephronophthisis in Dizygotic Twins. Internal medicine (Tokyo, Japan). 2023;62(1):87-90. https://doi.org/10.2169/internalmedicine.8707-21.

Olinger E, Phakdeekitcharoen P, Caliskan Y, Orr S, Mabillard H, Pickles C, et al. Biallelic variants in TTC21B as a rare cause of early-onset arterial hypertension and tubuloglomerular kidney disease. American journal of medical genetics Part C, Seminars in medical genetics. 2022;190(1):109-20. https://doi.org/10.1002/ajmg.c.31964.

Omran H, Häffner K, Burth S, Fernandez C, Fargier B, Villaquiran A, et al. Human adolescent nephronophthisis: gene locus synteny with polycystic kidney disease in pcy mice. Journal of the American Society of Nephrology : JASN. 2001;12(1):107-13. https://doi.org/10.1681/asn.V121107.

Otto E, Betz R, Rensing C, Schätzle S, Kuntzen T, Vetsi T, et al. A deletion distinct from the classical homologous recombination of juvenile nephronophthisis type 1 (NPH1) allows exact molecular definition of deletion breakpoints. Human mutation. 2000;16(3):211-23. https://doi.org/10.1002/1098-1004(200009).

Ozçay F, Derbent M, Demirhan B, Tokel K, Saatçi U. A family with Jeune syndrome. Pediatr Nephrol. 2001;16(8):623-6. https://doi.org/10.1007/s004670100627.

Patel R, Pillutla K, Thoreson L. Case 3: Fatigue, Weight Loss, Pallor, and Polydipsia in 12-year-old Girl. Pediatrics in review. 2015;36(11):508-10. https://doi.org/10.1542/pir.36-11-508.

Penchev V, Boueva A, Kamenarova K, Roussinov D, Tzveova R, Ivanova M, et al. A familial case of severe infantile nephronophthisis explained by oligogenic inheritance. European journal of medical genetics. 2017;60(6):321-5. https://doi.org/10.1016/j.ejmg.2017.04.002.

Qiu L, Zhou J. Simultaneous mutations of LAMB2 and NPHP1genes in a Chinese girl with isolated congenital nephrotic syndrome: a case report. BMC pediatrics. 2016;16:44. https://doi.org/10.1186/s12887-016-0583-0.

Raafat F, Morita M, Lau M, Taylor CM, White RH. Juvenile nephronophthisis with calcification of basal ganglia and pancreatic insufficiency. Archives of pathology & laboratory medicine. 1988;112(6):630-3.

Rajagopalan R, Grochowski CM, Gilbert MA, Falsey AM, Coleman K, Romero R, et al. Compound heterozygous mutations in NEK8 in siblings with end-stage renal disease with hepatic and cardiac anomalies. American journal of medical genetics Part A. 2016;170(3):750-3. https://doi.org/10.1002/ajmg.a.37512.

Ring E, Zobel G, Ratschek M, Trop M, Wendler H. Retrospective diagnosis of Jeune's syndrome in two patients with chronic renal failure. Child nephrology and urology. 1990;10(2):88-91. https://typeset.io/papers/retrospective-diagnosis-of-jeune-s-syndrome-in-two-patients-49csjldwe3.

Saito H, Takahashi Y, Takahashi S. Measurement of blood pressure to detect elusive kidney disease. Pediatrics international : official journal of the Japan Pediatric Society. 2017;59(5):638-9. https://doi.org/10.1111/ped.13263.

Schwarz H, Popp B, Airik R, Torabi N, Knaup KX, Stoeckert J, et al. Biallelic ANKS6 mutations cause late-onset ciliopathy with chronic kidney disease through YAP dysregulation. Human molecular genetics. 2022;31(9):1357-69. https://doi.org/10.1093/hmg/ddab322.

Sekiya K, Nakazawa M, Tanaka H. A Japanese child with Senior-Loken syndrome. Japanese journal of ophthalmology. 2001;45(6):636-9. https://doi.org/10.1016/s0021-5155(01)00424-5.

Sentell ZT, Nurcombe ZW, Mougharbel L, Anastasio N, Rivière JB, Babayeva S, et al. Expanding the phenotypic spectrum of CC2D2A-related ciliopathies: a rare homozygous nonsense variant in a patient with suspected nephronophthisis. European journal of human genetics : EJHG. 2024;32(9):1184-9. https://doi.org/10.1038/s41431-024-01668-x.

Shaik L, Ravalani A, Nelekar S, Gorijala VK, Shah K. Joubert Syndrome: A Molar Tooth Sign in Disguise. Cureus. 2020;12(8):e9718. https://doi.org/10.7759/cureus.9718.

Singh V, Bhattacharjee S, Singh K, Narula MK. Leber's amaurosis with nephronophthisis and congenital hepatic fibrosis. Indian pediatrics. 2004;41(10):1053-6. https://www.researchgate.net/publication/8197738_Leber's_amaurosis_with_nephronophthisis_and_congenital_hepatic_fibrosis.

Slater B, Bekheirnia N, Angelo J, Bi W, Braun MC, Bekheirnia MR. Nephronophthisis due to a novel DCDC2 variant in a patient from African-Caribbean descent: A case report. American journal of medical genetics Part A. 2020;182(3):527-31. https://doi.org/10.1002/ajmg.a.61440.

Somashekar PH, Upadhyai P, Shukla A, Girisha KM. Novel splice site and nonsense variants in INVS cause infantile nephronophthisis. Gene. 2020;729:144229. https://doi.org/10.1016/j.gene.2019.144229.

Sönmez F, Güzünler-Şen M, Yılmaz D, Cömertpay G, Heise M, Çırak S, et al. Development of end-stage renal disease at a young age in two cases with Joubert syndrome. The Turkish journal of pediatrics. 2014;56(4):458-61. https://turkjpediatr.org/article/view/1401/1394.

Stafrace S, Khan J. End-stage juvenile nephronophthisis on MRI. Pediatric radiology. 2010;40 Suppl 1:S12. https://doi.org/10.1007/s00247-009-1535-8.

Steinman B, Del Rio M, Zolotnitskaya A, Hayde N. A 5-year-old girl with kidney impairment and severe anemia: Answers. Pediatr Nephrol. 2023;38(2):393-6. https://doi.org/10.1007/s00467-022-05608-z.

Stone EM, Cideciyan AV, Aleman TS, Scheetz TE, Sumaroka A, Ehlinger MA, et al. Variations in NPHP5 in patients with nonsyndromic leber congenital amaurosis and Senior-Loken syndrome. Archives of ophthalmology (Chicago, Ill : 1960). 2011;129(1):81-7. https://doi.org/10.1001/archophthalmol.2010.330.

Strong A, Muneeruddin S, Parrish R, Lui D, Conley SB. Isosorbide dinitrate in nephronophthisis treatment. American journal of medical genetics Part A. 2018;176(4):1023-6. https://doi.org/10.1002/ajmg.a.38650.

Sun S, Chen L, Li N, Wang X. Homozygosity for a novel missense variant of RPGRIP1L causing Joubert syndrome with renal defects in a family of Chinese descent. Clinical nephrology. 2021;96(4):243-50. https://doi.org/10.5414/cn110539.

Takahashi T, Sato Y, Yamazaki T, Hayashi A, Okamoto T. Nephronophthisis with brown tumor: Old and new problems. Pediatrics international : official journal of the Japan Pediatric Society. 2017;59(9):1024-5. https://doi.org/10.1111/ped.13341.

Takano K, Nakamoto T, Okajima M, Sudo A, Uetake K, Saitoh S. Cerebellar and brainstem involvement in familial juvenile nephronophthisis type I. Pediatric neurology. 2003;28(2):142-4. https://doi.org/10.1016/s0887-8994(02)00619-7.

Tarrass F, Benjelloun M, Hachim K, Benghanem MG, Ramdani B, Zaid D, et al. Ehlers-Danlos syndrome coexisting with juvenile nephronophtisis. Nephrology (Carlton). 2006;11(2):117-9. https://doi.org/10.1111/j.1440-1797.2006.00498.x.

Ticho B, Sieving PA. Leber's congenital amaurosis with marbelized fundus and juvenile nephronophthisis. American journal of ophthalmology. 1989;107(4):426-8. https://doi.org/10.1016/0002-9394(89)90670-3.

Tkemaladze T, Melikishvili G, Kherkheulidze V, Melikishvili A, Davitaia T. EXPANDED PHENOTYPE OF TMEM67 GENE MUTATION (CASE REPORT). Georgian medical news. 2017(267):100-3. https://www.geomednews.com/s/480918712df344a4a77508d4cd7815ab/files/uploaded/V267_N6_June_2017.pdf?Expires=1651816093&Signature=EGwVSIVYFuAHbD8pGqISDstEbsMNV00t8SP3EvQIutceAGJYSwCg4kLWcdsUHOM-T4XtXH0qB218OzW0aTvdUU1yGkXitfV6~iGLT43nGGlzU3p3zcWxfAuVeiI5RUG4GgjHfzae9zY1I-d0LgwhE3fAd8KBTSxANL8fa87lUTLlqWpgIq9oQPaEh1CJ6uEohcy0BFcZNypDm-owzC8QVhPTPikwUgb3uT~G1pLjOU1PfQLF3OUKCoprR9U0MpRERUboYX4RxlOMTKzWXeLC6og5FQskiYCZz3l7AELH5JylcUl4bFJku2BORZxvhaV0Ei3cIKJoxZjib9DYWTVD1Q__&Key-Pair-Id=K2NXBXLF010TJW.

Tong H, Yue Z, Sun L, Chen H, Wang W, Wang H. Clinical features and mutation of NPHP5 in two Chinese siblings with Senior-Løken syndrome. Nephrology (Carlton). 2013;18(12):838-42. https://doi.org/10.1111/nep.12156.

Tsukahara H, Kikuchi K, Mikawa H, Fujisawa S, Yoshimoto M, Sudo M. Juvenile nephronophthisis diagnosed from glucosuria detected by urine screening at school. Acta paediatrica Japonica : Overseas edition. 1990;32(5):548-51. https://doi.org/10.1111/j.1442-200x.1990.tb00878.x.

Tsukamoto T, Tanaka M, Komiya T, Ueda S, Takasu K, Takahara S, et al. Nephronophthisis complicated with hepatic fibrosis: an autopsy case with rupture of the splenic artery after renal transplantation. Clinical and experimental nephrology. 2008;12(1):82-8. https://doi.org/10.1007/s10157-007-0004-7.

Udagawa T, Kamei K, Ogura M, Tsutsumi A, Noda S, Kasahara M, et al. Sequential liver-kidney transplantation in a boy with congenital hepatic fibrosis and nephronophthisis from a living donor. Pediatr Transplant. 2012;16(7):E275-80. https://doi.org/10.1111/j.1399-3046.2011.01611.x.

Veldman BCF, Kuper WFE, Lilien M, Schuurs-Hoeijmakers JHM, Marcelis C, Phan M, et al. Beyond nephronophthisis: Retinal dystrophy in the absence of kidney dysfunction in childhood expands the clinical spectrum of CEP83 deficiency. American journal of medical genetics Part A. 2021;185(7):2204-10. https://doi.org/10.1002/ajmg.a.62225.

Vnučák M, Graňák K, Skálová P, Laca Ľ, Mokáň M, Dedinská I. Living-Related Kidney Transplantation in a Patient with Juvenile Nephronophthisis. Nephron. 2020;144(11):583-8. https://doi.org/10.1159/000508501.

Volz A, Melkaoui R, Hildebrandt F, Omran H. Candidate gene analysis of KIAA0678 encoding a DnaJ-like protein for adolescent nephronophthisis and Senior-Løken syndrome type 3. Cytogenetic and genome research. 2002;97(3-4):163-6. https://doi.org/10.1159/000066617.

Walczak-Sztulpa J, Wawrocka A, Sobierajewicz A, Kuszel L, Zawadzki J, Grenda R, et al. Intrafamilial phenotypic variability in a Polish family with Sensenbrenner syndrome and biallelic WDR35 mutations. American journal of medical genetics Part A. 2017;173(5):1364-8. https://doi.org/10.1002/ajmg.a.38163.

Walczak-Sztulpa J, Wawrocka A, Swiader-Lesniak A, Socha M, Jamsheer A, Drozdz D, et al. Clinical and molecular genetic characterization of a male patient with Sensenbrenner syndrome (cranioectodermal dysplasia) and biallelic WDR35 mutations. Birth defects research. 2018;110(4):376-81. https://doi.org/10.1002/bdr2.1151.

Wang D, Chen X, Wen Q, Li Z, Chen W, Chen W, et al. A single heterozygous nonsense mutation in the TTC21B gene causes adult-onset nephronophthisis 12: A case report and review of literature. Molecular genetics & genomic medicine. 2022;10(12):e2076. https://doi.org/10.1002/mgg3.2076.

Wang Y, Chen F, Wang J, Zhao Y, Liu F. Two novel homozygous mutations in NPHP1 lead to late onset end-stage renal disease: a case report of an adult nephronophthisis in a Chinese intermarriage family. BMC Nephrol. 2019;20(1):173. https://doi.org/10.1186/s12882-019-1372-4.

Watanabe Y, Fujinaga S, Sakuraya K, Morisada N, Nozu K, Iijima K. Rapidly Progressive Nephronophthisis in a 2-Year-Old Boy with a Homozygous SDCCAG8 Mutation. The Tohoku journal of experimental medicine. 2019;249(1):29-32. https://doi.org/10.1620/tjem.249.29.

Yamamura T, Morisada N, Nozu K, Minamikawa S, Ishimori S, Toyoshima D, et al. Rare renal ciliopathies in non-consanguineous families that were identified by targeted resequencing. Clinical and experimental nephrology. 2017;21(1):136-42. https://doi.org/10.1007/s10157-016-1256-x.

Yasuda Y, Hashimoto R, Fukai R, Okamoto N, Hiraki Y, Yamamori H, et al. Duplication of the NPHP1 gene in patients with autism spectrum disorder and normal intellectual ability: a case series. Annals of general psychiatry. 2014;13:22. https://doi.org/10.1186/s12991-014-0022-2.

Yoshida A, Morozumi K, Koyama K, Takeda A, Uchida K, Tominaga Y, et al. Familial juvenile nephronophthisis and renal transplantation in two siblings. Nihon Jinzo Gakkai shi. 1992;34(9):1035-9. https://www.jstage.jst.go.jp/article/jpnjnephrol1959/34/9/34_9_1035/_pdf.

Yoshikawa T, Kamei K, Nagata H, Saida K, Sato M, Ogura M, et al. Diversity of renal phenotypes in patients with WDR19 mutations: Two case reports. Nephrology (Carlton). 2017;22(7):566-71. https://doi.org/10.1111/nep.12996.

Zhang H, Luo J, Liu L, Li J, Fu Q, Chen W, et al. Transplantation for infantile nephronophthisis with loss-of-function mutation in NPHP3: Lesson from a case. Pediatr Transplant. 2018:e13233. https://doi.org/10.1111/petr.13233.

Zhang H, Su B, Liu X, Xiao H, Ding J, Yao Y. Mutations in TTC21B cause different phenotypes in two childhood cases in China. Nephrology (Carlton). 2018;23(4):371-6. https://doi.org/10.1111/nep.13008.

Zhang H, Wang F, Xiao H, Yao Y. The ratio of urinary α1-microglobulin to microalbumin can be used as a diagnostic criterion for tubuloproteinuria. Intractable & rare diseases research. 2018;7(1):46-50. https://doi.org/10.5582/irdr.2017.01079.

Zhang X, Zhi X, Wang X, Dong Y, Shu J, Wang W, et al. Identification of a Splicing Variant c.3813-3A>G in NPHP3 by Reanalysis of Whole Exome Sequencing in a Chinese Boy with Nephronophthisis. Nephron. 2023;147(9):572-82. https://doi.org/10.1159/000529472.

Zhen Z, Dong Z, Gao L, Wang Q, Chen X, Na J, et al. Novel mutation in XPNPEP3 in a patient with heart failure without nephronophthisis-like nephropathy (NPHPL1): case report and literature review. BMC pediatrics. 2024;24(1):632. https://doi.org/10.1186/s12887-024-05124-z.

Zhong Z, Yan X, Fang Z, Dong Y, Tan J, Xie J, et al. Case Report: Adolescent-Onset Isolated Nephronophthisis Caused by a Novel Homozygous Inversin Mutation. Frontiers in genetics. 2022;13:847397. https://doi.org/10.3389/fgene.2022.847397.

No human clinical outcomes

Airik M, Schüler M, McCourt B, Weiss AC, Herdman N, Lüdtke TH, et al. Loss of Anks6 leads to YAP deficiency and liver abnormalities. Human molecular genetics. 2020;29(18):3064-80. https://doi.org/10.1093/hmg/ddaa197.

Airik R, Airik M, Schueler M, Bates CM, Hildebrandt F. Roscovitine blocks collecting duct cyst growth in Cep164-deficient kidneys. Kidney Int. 2019;96(2):320-6. https://doi.org/10.1016/j.kint.2019.04.014.

Airik R, Schueler M, Airik M, Cho J, Ulanowicz KA, Porath JD, et al. SDCCAG8 Interacts with RAB Effector Proteins RABEP2 and ERC1 and Is Required for Hedgehog Signaling. PLoS One. 2016;11(5):e0156081. https://doi.org/10.1371/journal.pone.0156081.

Airik R, Slaats GG, Guo Z, Weiss AC, Khan N, Ghosh A, et al. Renal-retinal ciliopathy gene Sdccag8 regulates DNA damage response signaling. Journal of the American Society of Nephrology : JASN. 2014;25(11):2573-83. https://doi.org/10.1681/asn.2013050565.

Ala-Mello S, Sankila EM, Koskimies O, de la Chapelle A, Kääriäinen H. Molecular studies in Finnish patients with familial juvenile nephronophthisis exclude a founder effect and support a common mutation causing mechanism. J Med Genet. 1998;35(4):279-83. https://doi.org/10.1136/jmg.35.4.279.

Arai Y, Takami M, An Y, Matsuo-Takasaki M, Hemmi Y, Wakabayashi T, et al. Generation of two human induced pluripotent stem cell lines derived from two juvenile nephronophthisis patients with NPHP1 deletion. Stem cell research. 2020;45:101815. https://doi.org/10.1016/j.scr.2020.101815.

Benzing T, Gerke P, Höpker K, Hildebrandt F, Kim E, Walz G. Nephrocystin interacts with Pyk2, p130(Cas), and tensin and triggers phosphorylation of Pyk2. Proceedings of the National Academy of Sciences of the United States of America. 2001;98(17):9784-9. https://doi.org/10.1073/pnas.171269898.

Bertelli R, Ginevri F, Candiano G, Ciardi MR, Tarelli LT, Meroni M, et al. Tubular epithelium culture from nephronophthisis-affected kidneys: a new approach to molecular disorders of tubular cells. American journal of nephrology. 1990;10(6):463-9. https://doi.org/10.1159/000168170.

Bielas SL, Silhavy JL, Brancati F, Kisseleva MV, Al-Gazali L, Sztriha L, et al. Mutations in INPP5E, encoding inositol polyphosphate-5-phosphatase E, link phosphatidyl inositol signaling to the ciliopathies. Nature genetics. 2009;41(9):1032-6. https://doi.org/10.1038/ng.423.

Blackburn ATM, Miller RK. Modeling congenital kidney diseases in Xenopus laevis. Disease models & mechanisms. 2019;12(4). https://doi.org/10.1242/dmm.038604.

Borgal L, Habbig S, Hatzold J, Liebau MC, Dafinger C, Sacarea I, et al. The ciliary protein nephrocystin-4 translocates the canonical Wnt regulator Jade-1 to the nucleus to negatively regulate β-catenin signaling. The Journal of biological chemistry. 2012;287(30):25370-80. https://doi.org/10.1074/jbc.M112.385658.

Cevik S, Sanders AA, Van Wijk E, Boldt K, Clarke L, van Reeuwijk J, et al. Active transport and diffusion barriers restrict Joubert Syndrome-associated ARL13B/ARL-13 to an Inv-like ciliary membrane subdomain. PLoS genetics. 2013;9(12):e1003977. https://doi.org/10.1371/journal.pgen.1003977.

Donaldson JC, Dise RS, Ritchie MD, Hanks SK. Nephrocystin-conserved domains involved in targeting to epithelial cell-cell junctions, interaction with filamins, and establishing cell polarity. The Journal of biological chemistry. 2002;277(32):29028-35. https://doi.org/10.1074/jbc.M111697200.

Fliegauf M, Fröhlich C, Horvath J, Olbrich H, Hildebrandt F, Omran H. Identification of the human CYS1 gene and candidate gene analysis in Boichis disease. Pediatr Nephrol. 2003;18(6):498-505. https://doi.org/10.1007/s00467-003-1141-1.

Fliegauf M, Horvath J, von Schnakenburg C, Olbrich H, Müller D, Thumfart J, et al. Nephrocystin specifically localizes to the transition zone of renal and respiratory cilia and photoreceptor connecting cilia. Journal of the American Society of Nephrology : JASN. 2006;17(9):2424-33. https://doi.org/10.1681/asn.2005121351.

Garcia H, Serafin AS, Silbermann F, Porée E, Viau A, Mahaut C, et al. Agonists of prostaglandin E(2) receptors as potential first in class treatment for nephronophthisis and related ciliopathies. Proceedings of the National Academy of Sciences of the United States of America. 2022;119(18):e2115960119. https://doi.org/10.1073/pnas.2115960119.

Gattone VH, 2nd, Wang X, Harris PC, Torres VE. Inhibition of renal cystic disease development and progression by a vasopressin V2 receptor antagonist. Nature medicine. 2003;9(10):1323-6. https://doi.org/10.1038/nm935.

Ghosh AK, Hurd T, Hildebrandt F. 3D spheroid defects in NPHP knockdown cells are rescued by the somatostatin receptor agonist octreotide. American journal of physiology Renal physiology. 2012;303(8):F1225-9. https://doi.org/10.1152/ajprenal.00135.2012.

Habbig S, Bartram MP, Sägmüller JG, Griessmann A, Franke M, Müller RU, et al. The ciliopathy disease protein NPHP9 promotes nuclear delivery and activation of the oncogenic transcriptional regulator TAZ. Human molecular genetics. 2012;21(26):5528-38. https://doi.org/10.1093/hmg/dds408.

Heninger E, Otto E, Imm A, Caridi G, Hildebrandt F. Improved strategy for molecular genetic diagnostics in juvenile nephronophthisis. Am J Kidney Dis. 2001;37(6):1131-9. https://doi.org/10.1053/ajkd.2001.24514.

Hildebrandt F, Cybulla M, Strahm B, Nothwang HG, Singh-Sawhney I, Berz K, et al. Physical mapping of the gene for juvenile nephronophthisis (NPH1) by construction of a complete YAC contig of 7 Mb on chromosome 2q13. Cytogenetics and cell genetics. 1996;73(3):235-9. https://doi.org/10.1159/000134346.

Hildebrandt F, Otto E, Rensing C, Nothwang HG, Vollmer M, Adolphs J, et al. A novel gene encoding an SH3 domain protein is mutated in nephronophthisis type 1. Nature genetics. 1997;17(2):149-53. https://doi.org/10.1038/ng1097-149.

Hildebrandt F, Rensing C, Betz R, Sommer U, Birnbaum S, Imm A, et al. Establishing an algorithm for molecular genetic diagnostics in 127 families with juvenile nephronophthisis. Kidney Int. 2001;59(2):434-45. https://doi.org/10.1046/j.1523-1755.2001.059002434.x.

Hildebrandt F, Singh-Sawhney I, Schnieders B, Centofante L, Omran H, Pohlmann A, et al. Mapping of a gene for familial juvenile nephronophthisis: refining the map and defining flanking markers on chromosome 2. APN Study Group. Am J Hum Genet. 1993;53(6):1256-61. https://doi.org/10.1016/0888-7543(95)80034-J.

Hildebrandt F, Singh-Sawhney I, Schnieders B, Papenfuss T, Brandis M. Refined genetic mapping of a gene for familial juvenile nephronophthisis (NPH1) and physical mapping of linked markers. APN Study Group. Genomics. 1995;25(2):360-4. https://doi.org/10.1016/0888-7543(95)80034-j.

Hoff S, Epting D, Falk N, Schroda S, Braun DA, Halbritter J, et al. The nucleoside-diphosphate kinase NME3 associates with nephronophthisis proteins and is required for ciliary function during renal development. The Journal of biological chemistry. 2018;293(39):15243-55. https://doi.org/10.1074/jbc.RA117.000847.

Hu Q, Lai J, Chen H, Cai Y, Yue Z, Lin H, et al. Reducing GEF-H1 Expression Inhibits Renal Cyst Formation, Inflammation, and Fibrosis via RhoA Signaling in Nephronophthisis. International journal of molecular sciences. 2023;24(4). https://doi.org/10.3390/ijms24043504.

Huang L, Szymanska K, Jensen VL, Janecke AR, Innes AM, Davis EE, et al. TMEM237 is mutated in individuals with a Joubert syndrome related disorder and expands the role of the TMEM family at the ciliary transition zone. Am J Hum Genet. 2011;89(6):713-30. https://doi.org/10.1016/j.ajhg.2011.11.005.

Husson H, Moreno S, Smith LA, Smith MM, Russo RJ, Pitstick R, et al. Reduction of ciliary length through pharmacologic or genetic inhibition of CDK5 attenuates polycystic kidney disease in a model of nephronophthisis. Human molecular genetics. 2016;25(11):2245-55. https://doi.org/10.1093/hmg/ddw093.

Hynes AM, Giles RH, Srivastava S, Eley L, Whitehead J, Danilenko M, et al. Murine Joubert syndrome reveals Hedgehog signaling defects as a potential therapeutic target for nephronophthisis. Proceedings of the National Academy of Sciences of the United States of America. 2014;111(27):9893-8. https://doi.org/10.1073/pnas.1322373111.

Ikeda M, Takemura T, Hino S, Yoshioka K. Molecular cloning, expression, and chromosomal localization of a human tubulointerstitial nephritis antigen. Biochemical and biophysical research communications. 2000;268(1):225-30. https://doi.org/10.1006/bbrc.2000.2103.

Jain M, Kaiser RWJ, Bohl K, Hoehne M, Göbel H, Bartram MP, et al. Inactivation of Apoptosis Antagonizing Transcription Factor in tubular epithelial cells induces accumulation of DNA damage and nephronophthisis. Kidney Int. 2019;95(4):846-58. https://doi.org/10.1016/j.kint.2018.10.034.

Jauregui AR, Nguyen KC, Hall DH, Barr MM. The Caenorhabditis elegans nephrocystins act as global modifiers of cilium structure. The Journal of cell biology. 2008;180(5):973-88. https://doi.org/10.1083/jcb.200707090.

Jávorszky E, Morinière V, Kerti A, Balogh E, Pikó H, Saunier S, et al. QMPSF is sensitive and specific in the detection of NPHP1 heterozygous deletions. Clinical chemistry and laboratory medicine. 2017;55(6):809-16. https://doi.org/10.1515/cclm-2016-0819.

Jiang ST, Chiou YY, Wang E, Chien YL, Ho HH, Tsai FJ, et al. Essential role of nephrocystin in photoreceptor intraflagellar transport in mouse. Human molecular genetics. 2009;18(9):1566-77. https://doi.org/10.1093/hmg/ddp068.

Jiang ST, Chiou YY, Wang E, Lin HK, Lee SP, Lu HY, et al. Targeted disruption of Nphp1 causes male infertility due to defects in the later steps of sperm morphogenesis in mice. Human molecular genetics. 2008;17(21):3368-79. https://doi.org/10.1093/hmg/ddn231.

Jin H, Zhang Y, Liu D, Wang SS, Ding Q, Rastogi P, et al. Innate Immune Signaling Contributes to Tubular Cell Senescence in the Glis2 Knockout Mouse Model of Nephronophthisis. The American journal of pathology. 2020;190(1):176-89. https://doi.org/10.1016/j.ajpath.2019.09.013.

Kallakuri S, Yu JA, Li J, Li Y, Weinstein BM, Nicoli S, et al. Endothelial cilia are essential for developmental vascular integrity in zebrafish. Journal of the American Society of Nephrology : JASN. 2015;26(4):864-75. https://doi.org/10.1681/asn.2013121314.

Kishimoto K, Nomura J, Ellegood J, Fukumoto K, Lerch JP, Moreno-De-Luca D, et al. Behavioral and neuroanatomical analyses in a genetic mouse model of 2q13 duplication. Genes to cells : devoted to molecular & cellular mechanisms. 2017;22(5):436-51. https://doi.org/10.1111/gtc.12487.

Konrad M, Saunier S, Heidet L, Silbermann F, Benessy F, Calado J, et al. Large homozygous deletions of the 2q13 region are a major cause of juvenile nephronophthisis. Human molecular genetics. 1996;5(3):367-71. https://doi.org/10.1093/hmg/5.3.367.

Konrad M, Saunier S, Silbermann F, Benessy F, Le Paslier D, Weissenbach J, et al. A 11 Mb YAC-based contig spanning the familial juvenile nephronophthisis region (NPH1) located on chromosome 2q. Genomics. 1995;30(3):514-20. https://doi.org/10.1006/geno.1995.1272.

Lancaster MA, Louie CM, Silhavy JL, Sintasath L, Decambre M, Nigam SK, et al. Impaired Wnt-beta-catenin signaling disrupts adult renal homeostasis and leads to cystic kidney ciliopathy. Nature medicine. 2009;15(9):1046-54. https://doi.org/10.1038/nm.2010.

Larsen CP, Bonsib SM, Beggs ML, Wilson JD. Fluorescence in situ hybridization for the diagnosis of NPHP1 deletion-related nephronophthisis on renal biopsy. Human pathology. 2018;81:71-7. https://doi.org/10.1016/j.humpath.2018.06.021.

le Maire A, Weber T, Saunier S, Broutin I, Antignac C, Ducruix A, et al. Solution NMR structure of the SH3 domain of human nephrocystin and analysis of a mutation-causing juvenile nephronophthisis. Proteins. 2005;59(2):347-55. https://doi.org/10.1002/prot.20344.

Lee JW, Cho JY, Thuy PX, Moon EY. HeLa Cervical Cancer Cells Are Maintained by Nephronophthisis 3-Associated Primary Cilium Formation via ROS-Induced ERK and HIF-1α Activation under Serum-Deprived Normoxic Condition. International journal of molecular sciences. 2022;23(23). https://doi.org/10.3390/ijms232314500.

Lee JW, Kim HS, Moon EY. Thymosin β-4 is a novel regulator for primary cilium formation by nephronophthisis 3 in HeLa human cervical cancer cells. Sci Rep. 2019;9(1):6849. https://doi.org/10.1038/s41598-019-43235-1.

Li D, Hu M, Chen H, Wu X, Wei X, Lin H, et al. An Nphp1 knockout mouse model targeting exon 2-20 demonstrates characteristic phenotypes of human nephronophthisis. Human molecular genetics. 2021;31(2):232-43. https://doi.org/10.1093/hmg/ddab239.

Liu L, Zhang M, Xia Z, Xu P, Chen L, Xu T. Caenorhabditis elegans ciliary protein NPHP-8, the homologue of human RPGRIP1L, is required for ciliogenesis and chemosensation. Biochemical and biophysical research communications. 2011;410(3):626-31. https://doi.org/10.1016/j.bbrc.2011.06.041.

Louie CM, Caridi G, Lopes VS, Brancati F, Kispert A, Lancaster MA, et al. AHI1 is required for photoreceptor outer segment development and is a modifier for retinal degeneration in nephronophthisis. Nature genetics. 2010;42(2):175-80. https://doi.org/10.1038/ng.519.

Lu D, Rauhauser A, Li B, Ren C, McEnery K, Zhu J, et al. Loss of Glis2/NPHP7 causes kidney epithelial cell senescence and suppresses cyst growth in the Kif3a mouse model of cystic kidney disease. Kidney Int. 2016;89(6):1307-23. https://doi.org/10.1016/j.kint.2016.03.006.

Mannella V, Quilici G, Nigro EA, Lampis M, Minici C, Degano M, et al. The N-Terminal Domain of NPHP1 Folds into a Monomeric Left-Handed Antiparallel Three-Stranded Coiled Coil with Anti-

apoptotic Function. ACS chemical biology. 2019;14(8):1845-54. https://doi.org/10.1021/acschembio.9b00582.

McCooke JK, Appels R, Barrero RA, Ding A, Ozimek-Kulik JE, Bellgard MI, et al. A novel mutation causing nephronophthisis in the Lewis polycystic kidney rat localises to a conserved RCC1 domain in Nek8. BMC genomics. 2012;13:393. https://doi.org/10.1186/1471-2164-13-393.

Mollet G, Salomon R, Gribouval O, Silbermann F, Bacq D, Landthaler G, et al. The gene mutated in juvenile nephronophthisis type 4 encodes a novel protein that interacts with nephrocystin. Nature genetics. 2002;32(2):300-5. https://doi.org/10.1038/ng996.

Monirujjaman M, Devassy JG, Yamaguchi T, Sidhu N, Kugita M, Gabbs M, et al. Distinct oxylipin alterations in diverse models of cystic kidney diseases. Biochimica et biophysica acta Molecular and cell biology of lipids. 2017;1862(12):1562-74. https://doi.org/10.1016/j.bbalip.2017.08.005.

Nothwang HG, Stubanus M, Adolphs J, Hanusch H, Vossmerbäumer U, Denich D, et al. Construction of a gene map of the nephronophthisis type 1 (NPHP1) region on human chromosome 2q12-q13. Genomics. 1998;47(2):276-85. https://doi.org/10.1006/geno.1997.5102.

Okumura Y, Sugiyama N, Tanimura S, Nishida M, Hamaoka K, Kohno M, et al. ERK regulates renal cell proliferation and renal cyst expansion in inv mutant mice. Acta histochemica et cytochemica. 2009;42(2):39-45. https://doi.org/10.1267/ahc.08040.

Otto E, Hoefele J, Ruf R, Mueller AM, Hiller KS, Wolf MT, et al. A gene mutated in nephronophthisis and retinitis pigmentosa encodes a novel protein, nephroretinin, conserved in evolution. Am J Hum Genet. 2002;71(5):1161-7. https://doi.org/10.1086/344395.

Otto E, Kispert A, Schätzle S, Lescher B, Rensing C, Hildebrandt F. Nephrocystin: gene expression and sequence conservation between human, mouse, and Caenorhabditis elegans. Journal of the American Society of Nephrology : JASN. 2000;11(2):270-82. https://doi.org/10.1681/asn.V112270.

Oud MM, van Bon BW, Bongers EM, Hoischen A, Marcelis CL, de Leeuw N, et al. Early presentation of cystic kidneys in a family with a homozygous INVS mutation. American journal of medical genetics Part A. 2014;164a(7):1627-34. https://doi.org/10.1002/ajmg.a.36501.

Quatredeniers M, Bienaimé F, Ferri G, Isnard P, Porée E, Billot K, et al. The renal inflammatory network of nephronophthisis. Human molecular genetics. 2022;31(13):2121-36. https://doi.org/10.1093/hmg/ddac014.

Ramachandran H, Engel C, Müller B, Dengjel J, Walz G, Yakulov TA. Anks3 alters the sub-cellular localization of the Nek7 kinase. Biochemical and biophysical research communications. 2015;464(3):901-7. https://doi.org/10.1016/j.bbrc.2015.07.063.

Ramachandran H, Yakulov TA, Engel C, Müller B, Walz G. The C175R mutation alters nuclear localization and transcriptional activity of the nephronophthisis NPHP7 gene product. European journal of human genetics : EJHG. 2016;24(5):774-8. https://doi.org/10.1038/ejhg.2015.199.

Rao J, Liu X, Mao J, Tang X, Shen Q, Li G, et al. Genetic spectrum of renal disease for 1001 Chinese children based on a multicenter registration system. Clinical genetics. 2019;96(5):402-10. https://doi.org/10.1111/cge.13606.

Rasmussen M, Sunde L, Nielsen ML, Ramsing M, Petersen A, Hjortshøj TD, et al. Targeted gene sequencing and whole-exome sequencing in autopsied fetuses with prenatally diagnosed kidney anomalies. Clinical genetics. 2018;93(4):860-9. https://doi.org/10.1111/cge.13185.

Saunier S, Calado J, Benessy F, Silbermann F, Heilig R, Weissenbach J, et al. Characterization of the NPHP1 locus: mutational mechanism involved in deletions in familial juvenile nephronophthisis. Am J Hum Genet. 2000;66(3):778-89. https://doi.org/10.1086/302819.

Saunier S, Calado J, Heilig R, Silbermann F, Benessy F, Morin G, et al. A novel gene that encodes a protein with a putative src homology 3 domain is a candidate gene for familial juvenile nephronophthisis. Human molecular genetics. 1997;6(13):2317-23. https://doi.org/10.1093/hmg/6.13.2317.

Schlimpert M, Lagies S, Budnyk V, Müller B, Walz G, Kammerer B. Metabolic Phenotyping of Anks3 Depletion in mIMCD-3 cells - a Putative Nephronophthisis Candidate. Sci Rep. 2018;8(1):9022. https://doi.org/10.1038/s41598-018-27389-y.

Schönauer R, Jin W, Ertel A, Nemitz-Kliemchen M, Panitz N, Hantmann E, et al. Novel nephronophthisis-associated variants reveal functional importance of MAPKBP1 dimerization for centriolar recruitment. Kidney Int. 2020;98(4):958-69. https://doi.org/10.1016/j.kint.2020.05.027.

Schueler M, Halbritter J, Phelps IG, Braun DA, Otto EA, Porath JD, et al. Large-scale targeted sequencing comparison highlights extreme genetic heterogeneity in nephronophthisis-related ciliopathies. J Med Genet. 2016;53(3):208-14. https://doi.org/10.1136/jmedgenet-2015-103304.

Shi X, Garcia G, 3rd, Van De Weghe JC, McGorty R, Pazour GJ, Doherty D, et al. Super-resolution microscopy reveals that disruption of ciliary transition-zone architecture causes Joubert syndrome. Nature cell biology. 2017;19(10):1178-88. https://doi.org/10.1038/ncb3599.

Slaats GG, Ghosh AK, Falke LL, Le Corre S, Shaltiel IA, van de Hoek G, et al. Nephronophthisis-associated CEP164 regulates cell cycle progression, apoptosis and epithelial-to-mesenchymal transition. PLoS genetics. 2014;10(10):e1004594. https://doi.org/10.1371/journal.pgen.1004594.

Srivastava S, Ramsbottom SA, Molinari E, Alkanderi S, Filby A, White K, et al. A human patient-derived cellular model of Joubert syndrome reveals ciliary defects which can be rescued with targeted therapies. Human molecular genetics. 2017;26(23):4657-67. https://doi.org/10.1093/hmg/ddx347.

Sugimoto K, Takemura Y, Yanagida H, Fujita S, Miyazawa T, Sakata N, et al. Renal tubular dysgenesis and tubulointerstitial nephritis antigen in juvenile nephronophthisis. Nephrology (Carlton). 2011;16(5):495-501. https://doi.org/10.1111/j.1440-1797.2011.01442.x.

Sugiyama N, Kohno M, Yokoyama T. Inhibition of the p38 MAPK pathway ameliorates renal fibrosis in an NPHP2 mouse model. Nephrol Dial Transplant. 2012;27(4):1351-8. https://doi.org/10.1093/ndt/gfr550.

Thomas CC, Jana M, Sinha A, Bagga A, Ramachandran A, Sudhakaran D, et al. Ultrasound Imaging of Renal Cysts in Children. Journal of ultrasound in medicine : official journal of the American Institute of Ultrasound in Medicine. 2021;40(3):621-35. https://doi.org/10.1002/jum.15435.

Tobin JL, Beales PL. Restoration of renal function in zebrafish models of ciliopathies. Pediatr Nephrol. 2008;23(11):2095-9. https://doi.org/10.1007/s00467-008-0898-7.

Vasanth S, ZeRuth G, Kang HS, Jetten AM. Identification of nuclear localization, DNA binding, and transactivating mechanisms of Kruppel-like zinc finger protein Gli-similar 2 (Glis2). The Journal of biological chemistry. 2011;286(6):4749-59. https://doi.org/10.1074/jbc.M110.165951.

Wallace DP, White C, Savinkova L, Nivens E, Reif GA, Pinto CS, et al. Periostin promotes renal cyst growth and interstitial fibrosis in polycystic kidney disease. Kidney Int. 2014;85(4):845-54. https://doi.org/10.1038/ki.2013.488.

Weihbrecht K, Goar WA, Carter CS, Sheffield VC, Seo S. Genotypic and phenotypic characterization of the Sdccag8Tn(sb-Tyr)2161B.CA1C2Ove mouse model. PLoS One. 2018;13(2):e0192755. https://doi.org/10.1371/journal.pone.0192755.

Winkelbauer ME, Schafer JC, Haycraft CJ, Swoboda P, Yoder BK. The C. elegans homologs of nephrocystin-1 and nephrocystin-4 are cilia transition zone proteins involved in chemosensory perception. Journal of cell science. 2005;118(Pt 23):5575-87. https://doi.org/10.1242/jcs.02665.

Wolf MT, Lee J, Panther F, Otto EA, Guan KL, Hildebrandt F. Expression and phenotype analysis of the nephrocystin-1 and nephrocystin-4 homologs in Caenorhabditis elegans. Journal of the American Society of Nephrology : JASN. 2005;16(3):676-87. https://doi.org/10.1681/asn.2003121025.

Wu X, Wang H, Chen H, Lin H, Li M, Yue Z, et al. Overexpression of smad7 inhibits the TGF-β/Smad signaling pathway and EMT in NPHP1-defective MDCK cells. Biochemical and biophysical research communications. 2021;582:57-63. https://doi.org/10.1016/j.bbrc.2021.10.037.

Yamaguchi T, Devassy JG, Gabbs M, Ravandi A, Nagao S, Aukema HM. Dietary flax oil rich in α-linolenic acid reduces renal disease and oxylipin abnormalities, including formation of docosahexaenoic acid derived oxylipins in the CD1-pcy/pcy mouse model of nephronophthisis. Prostaglandins, leukotrienes, and essential fatty acids. 2015;94:83-9. https://doi.org/10.1016/j.plefa.2014.11.009.

Yamaguchi T, Lysecki C, Reid A, Nagao S, Aukema HM. Renal cyclooxygenase products are higher and lipoxygenase products are lower in early disease in the pcy mouse model of adolescent nephronophthisis. Lipids. 2014;49(1):39-47. https://doi.org/10.1007/s11745-013-3859-2.

Yuan B, Liu P, Gupta A, Beck CR, Tejomurtula A, Campbell IM, et al. Comparative Genomic Analyses of the Human NPHP1 Locus Reveal Complex Genomic Architecture and Its Regional Evolution in Primates. PLoS genetics. 2015;11(12):e1005686. https://doi.org/10.1371/journal.pgen.1005686.

Zhang Y, Seo S, Bhattarai S, Bugge K, Searby CC, Zhang Q, et al. BBS mutations modify phenotypic expression of CEP290-related ciliopathies. Human molecular genetics. 2014;23(1):40-51. https://doi.org/10.1093/hmg/ddt394.

Zhou W, Dai J, Attanasio M, Hildebrandt F. Nephrocystin-3 is required for ciliary function in zebrafish embryos. American journal of physiology Renal physiology. 2010;299(1):F55-62. https://doi.org/10.1152/ajprenal.00043.2010.

Ziegler WH, Lüdiger S, Hassan F, Georgiadis ME, Swolana K, Khera A, et al. Primary URECs: a source to better understand the pathology of renal tubular epithelia in pediatric hereditary cystic kidney diseases. Orphanet J Rare Dis. 2022;17(1):122. https://doi.org/10.1186/s13023-022-02265-1.

No original data

Arora V, Anand K, Chander Verma I. Genetic Testing in Pediatric Kidney Disease. Indian journal of pediatrics. 2020;87(9):706-15. https://doi.org/10.1007/s12098-020-03198-y.

Benzing T, Schermer B. Clinical spectrum and pathogenesis of nephronophthisis. Current opinion in nephrology and hypertension. 2012;21(3):272-8. https://doi.org/10.1097/MNH.0b013e3283520f17.

Bergmann C. Early and Severe Polycystic Kidney Disease and Related Ciliopathies: An Emerging Field of Interest. Nephron. 2019;141(1):50-60. https://doi.org/10.1159/000493532.

Braun DA, Hildebrandt F. Ciliopathies. Cold Spring Harb Perspect Biol. 2017;9(3). https://doi.org/10.1101/cshperspect.a028191.

Caridi G, Dagnino M, Miglietti N, Carrea A, Perfumo F, Gusmano R, et al. Juvenile nephronophthisis and related variants: clinical features and molecular approach. Contributions to nephrology. 2001(136):57-67. https://doi.org/10.1159/000060179.

Chance PF, Cavalier L, Satran D, Pellegrino JE, Koenig M, Dobyns WB. Clinical nosologic and genetic aspects of Joubert and related syndromes. Journal of child neurology. 1999;14(10):660-6; discussion 9-72. https://doi.org/10.1177/088307389901401007.

Chen X, Shi Z, Yang F, Zhou T, Xie S. Deciphering cilia and ciliopathies using proteomic approaches. The FEBS journal. 2022;290(10):2590-603. https://doi.org/10.1111/febs.16538.

Chung EM, Conran RM, Schroeder JW, Rohena-Quinquilla IR, Rooks VJ. From the radiologic pathology archives: pediatric polycystic kidney disease and other ciliopathies: radiologic-pathologic correlation. Radiographics : a review publication of the Radiological Society of North America, Inc. 2014;34(1):155-78. https://doi.org/10.1148/rg.341135179.

Cramer MT, Guay-Woodford LM. Cystic kidney disease: a primer. Advances in chronic kidney disease. 2015;22(4):297-305. https://doi.org/10.1053/j.ackd.2015.04.001.

Finer G, Shalev H, Landau D. Genetic kidney diseases in the pediatric population of southern Israel. Pediatr Nephrol. 2006;21(7):910-6. https://doi.org/10.1007/s00467-006-0142-2.

Getwan M, Hoppmann A, Schlosser P, Grand K, Song W, Diehl R, et al. Ttc30a affects tubulin modifications in a model for ciliary chondrodysplasia with polycystic kidney disease. Proceedings of the National Academy of Sciences of the United States of America. 2021;118(39). https://doi.org/10.1073/pnas.2106770118.

Grochowsky A, Gunay-Aygun M. Clinical characteristics of individual organ system disease in non-motile ciliopathies. Translational science of rare diseases. 2019;4(1-2):1-23. https://doi.org/10.3233/trd-190033.

Gupta S, Ozimek-Kulik JE, Phillips JK. Nephronophthisis-Pathobiology and Molecular Pathogenesis of a Rare Kidney Genetic Disease. Genes. 2021;12(11). https://doi.org/10.3390/genes12111762.

Gusmano R, Ghiggeri GM, Caridi G. Nephronophthisis-medullary cystic disease: clinical and genetic aspects. Journal of nephrology. 1998;11(5):224-8.

Habbig S, Liebau MC. Ciliopathies - from rare inherited cystic kidney diseases to basic cellular function. Molecular and cellular pediatrics. 2015;2(1):8. https://doi.org/10.1186/s40348-015-0019-1.

Hildebrandt F. Identification of a gene for nephronophthisis. Nephrol Dial Transplant. 1998;13(6):1334-6. https://doi.org/10.1093/ndt/13.6.1334.

Hildebrandt F, Attanasio M, Otto E. Nephronophthisis: disease mechanisms of a ciliopathy. Journal of the American Society of Nephrology : JASN. 2009;20(1):23-35. https://doi.org/10.1681/asn.2008050456.

Hildebrandt F, Benzing T, Katsanis N. Ciliopathies. N Engl J Med. 2011;364(16):1533-43. https://doi.org/10.1056/NEJMra1010172.

Hildebrandt F, Omram H. New insights: nephronophthisis-medullary cystic kidney disease. Pediatr Nephrol. 2001;16(2):168-76. https://doi.org/10.1007/s004670000518.

Hildebrandt F, Otto E. Molecular genetics of nephronophthisis and medullary cystic kidney disease. Journal of the American Society of Nephrology : JASN. 2000;11(9):1753-61. https://doi.org/10.1681/asn.V1191753.

Hildebrandt F, Waldherr R, Kutt R, Brandis M. The nephronophthisis complex: clinical and genetic aspects. The Clinical investigator. 1992;70(9):802-8. https://doi.org/10.1007/bf00180751.

Hildebrandt F, Zhou W. Nephronophthisis-associated ciliopathies. Journal of the American Society of Nephrology : JASN. 2007;18(6):1855-71. https://doi.org/10.1681/asn.2006121344.

Hurd TW, Hildebrandt F. Mechanisms of nephronophthisis and related ciliopathies. Nephron Experimental nephrology. 2011;118(1):e9-14. https://doi.org/10.1159/000320888.

Johnson CA, Gissen P, Sergi C. Molecular pathology and genetics of congenital hepatorenal fibrocystic syndromes. J Med Genet. 2003;40(5):311-9. https://doi.org/10.1136/jmg.40.5.311.

Knotek M, Novak R, Jaklin-Kekez A, Mrzljak A. Combined liver-kidney transplantation for rare diseases. World journal of hepatology. 2020;12(10):722-37. https://doi.org/10.4254/wjh.v12.i10.722.

König JC, Titieni A, Konrad M. Network for Early Onset Cystic Kidney Diseases-A Comprehensive Multidisciplinary Approach to Hereditary Cystic Kidney Diseases in Childhood. Front Pediatr. 2018;6:24. https://doi.org/10.3389/fped.2018.00024.

Krishnan R, Eley L, Sayer JA. Urinary concentration defects and mechanisms underlying nephronophthisis. Kidney & blood pressure research. 2008;31(3):152-62. https://doi.org/10.1159/000129648.

Lina F, Satlinb LM. Polycystic kidney disease: the cilium as a common pathway in cystogenesis. Current opinion in pediatrics. 2004;16(2):171-6. https://doi.org/10.1097/00008480-200404000-00010.

Luo F, Tao YH. Nephronophthisis: A review of genotype-phenotype correlation. Nephrology (Carlton). 2018;23(10):904-11. https://doi.org/10.1111/nep.13393.

Mattoo TK. Genetically transmitted renal diseases in children: a saudi perspective. Saudi J Kidney Dis Transpl. 1998;9(2):105-9. https://journals.lww.com/sjkd/fulltext/1998/09020/genetically_transmitted_renal_diseases_in.1.aspx.

Mochizuki T, Makabe S, Aoyama Y, Kataoka H, Nitta K. New Insights into Cystic Kidney Diseases. Contributions to nephrology. 2018;195:31-41. https://doi.org/10.1159/000486932.

Müller RU, Benzing T. Cystic Kidney Diseases From the Adult Nephrologist's Point of View. Front Pediatr. 2018;6:65. https://doi.org/10.3389/fped.2018.00065.

Müller RU, Schermer B. Hippo signaling-a central player in cystic kidney disease? Pediatr Nephrol. 2020;35(7):1143-52. https://doi.org/10.1007/s00467-019-04299-3.

Omran H, Häffner K, Burth S, Ala-Mello S, Antignac C, Hildebrandt F. Evidence for further genetic heterogeneity in nephronophthisis. Nephrol Dial Transplant. 2001;16(4):755-8. https://doi.org/10.1093/ndt/16.4.755.

Parisi MA. Clinical and molecular features of Joubert syndrome and related disorders. American journal of medical genetics Part C, Seminars in medical genetics. 2009;151c(4):326-40. https://doi.org/10.1002/ajmg.c.30229.

Parisi MA. The molecular genetics of Joubert syndrome and related ciliopathies: The challenges of genetic and phenotypic heterogeneity. Translational science of rare diseases. 2019;4(1-2):25-49. https://doi.org/10.3233/trd-190041.

Raina R, Chakraborty R, Sethi SK, Kumar D, Gibson K, Bergmann C. Diagnosis and Management of Renal Cystic Disease of the Newborn: Core Curriculum 2021. Am J Kidney Dis. 2021;78(1):125-41. https://doi.org/10.1053/j.ajkd.2020.10.021.

Renkema KY, Giles RH, Lilien MR, Beales PL, Roepman R, Oud MM, et al. The KOUNCIL Consortium: From Genetic Defects to Therapeutic Development for Nephronophthisis. Front Pediatr. 2018;6:131. https://doi.org/10.3389/fped.2018.00131.

Saborio P, Scheinman J. Genetic renal disease. Current opinion in pediatrics. 1998;10(2):174-83. https://doi.org/10.1097/00008480-199804000-00011.

Salomon R, Saunier S, Niaudet P. Nephronophthisis. Pediatr Nephrol. 2009;24(12):2333-44. https://doi.org/10.1007/s00467-008-0840-z.

Satran D, Pierpont ME, Dobyns WB. Cerebello-oculo-renal syndromes including Arima, Senior-Löken and COACH syndromes: more than just variants of Joubert syndrome. American journal of medical genetics. 1999;86(5):459-69. https://doi.org/10.1002/(sici)1096-8628(19991029)86:5<459::aid-ajmg12>3.0.co;2-c.

Sattar S, Gleeson JG. The ciliopathies in neuronal development: a clinical approach to investigation of Joubert syndrome and Joubert syndrome-related disorders. Dev Med Child Neurol. 2011;53(9):793-8. https://doi.org/10.1111/j.1469-8749.2011.04021.x.

Scolari F, Ghiggeri GM. Nephronophthisis-medullary cystic kidney disease: from bedside to bench and back again. Saudi J Kidney Dis Transpl. 2003;14(3):316-27. https://journals.lww.com/sjkd/fulltext/2003/14030/nephronophthisis_medullary_cystic_kidney_disease_.6.aspx.

Scolari F, Viola BF, Ghiggeri GM, Caridi G, Amoroso A, Rampoldi L, et al. Towards the identification of (a) gene(s) for autosomal dominant medullary cystic kidney disease. Journal of nephrology. 2003;16(3):321-8. https://www.researchgate.net/publication/10685079_Towards_the_identification_of_a_genes_for_autosomal_dominant_medullary_cystic_kidney_disease.

Seeman T, Seemanová E, Nuernberg G, Nuernberg P, Janssen S, Otto EA. Polycystic kidney and hepatic disease with mental retardation is nephronophthisis 11 caused by MKS3/TMEM67 mutations. Pediatr Nephrol. 2010;25(11):2375-6. https://doi.org/10.1007/s00467-010-1591-1.

Sekine A, Hidaka S, Moriyama T, Shikida Y, Shimazu K, Ishikawa E, et al. Cystic Kidney Diseases That Require a Differential Diagnosis from Autosomal Dominant Polycystic Kidney Disease (ADPKD). J Clin Med. 2022;11(21). https://doi.org/10.3390/jcm11216528.

Simms RJ, Hynes AM, Eley L, Sayer JA. Nephronophthisis: a genetically diverse ciliopathy. International journal of nephrology. 2011;2011:527137. https://doi.org/10.4061/2011/527137.

Simms RJ, Sayer JA. Juvenile nephronophthisis on MRI--a potential case of Joubert syndrome? Pediatric radiology. 2010;40(9):1581; author reply 2. https://doi.org/10.1007/s00247-010-1707-6.

Slaats GG, Lilien MR, Giles RH. Nephronophthisis: should we target cysts or fibrosis? Pediatr Nephrol. 2016;31(4):545-54. https://doi.org/10.1007/s00467-015-3162-y.

Srivastava S, Molinari E, Raman S, Sayer JA. Many Genes-One Disease? Genetics of Nephronophthisis (NPHP) and NPHP-Associated Disorders. Front Pediatr. 2017;5:287. https://doi.org/10.3389/fped.2017.00287.

Stokman MF, Saunier S, Benmerah A. Renal Ciliopathies: Sorting Out Therapeutic Approaches for Nephronophthisis. Front Cell Dev Biol. 2021;9:653138. https://doi.org/10.3389/fcell.2021.653138.

Tsang SH, Aycinena ARP, Sharma T. Ciliopathy: Senior-Løken Syndrome. Advances in experimental medicine and biology. 2018;1085:175-8. https://doi.org/10.1007/978-3-319-95046-4_34.

Valente EM, Dallapiccola B, Bertini E. Joubert syndrome and related disorders. Handbook of clinical neurology. 2013;113:1879-88. https://doi.org/10.1016/b978-0-444-59565-2.00058-7.

Van De Weghe JC, Gomez A, Doherty D. The Joubert-Meckel-Nephronophthisis Spectrum of Ciliopathies. Annual review of genomics and human genetics. 2022;23:301-29. https://doi.org/10.1146/annurev-genom-121321-093528.

Viršilas E, Čerkauskienė R, Masalskienė J, Rudaitis Š, Dobilienė D, Jankauskienė A. Renal Replacement Therapy in Children in Lithuania: Challenges, Trends, and Outcomes. Medicina (Kaunas, Lithuania). 2018;54(5). https://doi.org/10.3390/medicina54050078.

Wilson PD. Polycystic kidney disease: new understanding in the pathogenesis. The international journal of biochemistry & cell biology. 2004;36(10):1868-73. https://doi.org/10.1016/j.biocel.2004.03.012.

Wolf MT. Nephronophthisis and related syndromes. Current opinion in pediatrics. 2015;27(2):201-11. https://doi.org/10.1097/mop.0000000000000194.

Wolf MT, Hildebrandt F. Nephronophthisis. Pediatr Nephrol. 2011;26(2):181-94. https://doi.org/10.1007/s00467-010-1585-z.

Yuan S, Sun Z. Expanding horizons: ciliary proteins reach beyond cilia. Annual review of genetics. 2013;47:353-76. https://doi.org/10.1146/annurev-genet-111212-133243.

Not covering NPH

Mixtard 30 HM Penfill insulin treatment in selected young insulin-dependent diabetes mellitus patients. Romanian Young Diabetics Study Team (RYDST). Romanian journal of internal medicine = Revue roumaine de medecine interne. 1997;35(1-4):99-113.

Abacı A, Razi CH, Ozdemir O, Hızlı S, Kıslal F, Argas PI, et al. Neonatal diabetes mellitus accompanied by diabetic ketoacidosis and mimicking neonatal sepsis: a case report. Journal of clinical research in pediatric endocrinology. 2010;2(3):131-3. https://doi.org/10.4274/jcrpe.v2i3.131

Abdelgadir E, Al Sahlawi M, Al Turki L, Khamees K, Ahmed W. Identification of a new homozygous CEP290 gene mutation in a Saudi Family causing joubert syndrome using next-generation sequencing. Saudi J Kidney Dis Transpl. 2019;30(4):964-8. https://doi.org/10.4103/1319-2442.265475.

Abeywickrama CS, Baumann HJ, Bertman KA, Corbin B, Pang Y. The Unexpected Selectivity Switching from Mitochondria to Lysosome in a D-π-A Cyanine Dye. Biosensors. 2022;12(7). https://doi.org/10.3390/bios12070504.

Abraham D, Karuvattil R, Fitzpatrick M. Stridor in an 11-year-old child. BMJ case reports. 2013;2013. https://doi.org/10.1136/bcr-2013-201025.

Agarwal N, Kashkoush A, McDowell MM, Lariviere WR, Ismail N, Friedlander RM. Comparative durability and costs analysis of ventricular shunts. Journal of neurosurgery. 2018:1-8. https://doi.org/10.3171/2017.11.Jns172212.

Agrawal A, Bhattacharya A, Kamble N, Yadav R, Pal PK. Effect of Lumbar Drainage on Cortical Excitability in Normal Pressure Hydrocephalus. The Canadian journal of neurological sciences Le journal canadien des sciences neurologiques. 2021;48(2):253-8. https://doi.org/10.1017/cjn.2020.169.

Ahern JA, Ramchandani N, Cooper J, Himmel A, Silver D, Tamborlane WV. Using a primary nurse manager to implement DCCT recommendations in a large pediatric program. The Diabetes educator. 2000;26(6):990-4. https://doi.org/10.1177/014572170002600611.

Airik M, McCourt B, Ozturk TT, Huynh AB, Zhang X, Tometich JT, et al. Mitigation of portal fibrosis and cholestatic liver disease in ANKS6-deficient livers by macrophage depletion. FASEB journal : official publication of the Federation of American Societies for Experimental Biology. 2022;36(2):e22157. https://doi.org/10.1096/fj.202101387R.

Akizu N, Silhavy JL, Rosti RO, Scott E, Fenstermaker AG, Schroth J, et al. Mutations in CSPP1 lead to classical Joubert syndrome. Am J Hum Genet. 2014;94(1):80-6. https://doi.org/10.1016/j.ajhg.2013.11.015.

Aksu Uzunhan T, Ertürk B, Aydın K, Ayaz A, Altunoğlu U, Yarar MH, et al. Clinical and genetic spectrum from a prototype of ciliopathy: Joubert syndrome. Clinical neurology and neurosurgery. 2022;224:107560. https://doi.org/10.1016/j.clineuro.2022.107560.

Al-Rubeaan KA, Al-Daghri NM, Alkharfy KM, Al-Attas OS, Hanif FS, Metias NS, et al. Bioequivalence of Jusline following subcutaneous administration in healthy subjects. International journal of clinical pharmacology and therapeutics. 2008;46(7):382-8. https://doi.org/10.5414/cpp46382.

Alemzadeh R, Palma-Sisto P, Parton E, Totka J, Kirby M. Beneficial effects of flexible insulin therapy in children and adolescents with type 1 diabetes mellitus. Acta diabetologica. 2003;40(3):137-42. https://doi.org/10.1007/s00592-003-0102-2.

Alves C, Sherbini O, D'Arco F, Steel D, Kurian MA, Radio FC, et al. Brain Abnormalities in Patients with Germline Variants in H3F3: Novel Imaging Findings and Neurologic Symptoms Beyond Somatic Variants and Brain Tumors. AJNR American journal of neuroradiology. 2022;43(7):1048-53. https://doi.org/10.3174/ajnr.A7555.

Amini FG, Nia AF, Sharbafi MH, Khandari A, Gargari SS. Comparison between the effect of regular human insulin and NPH with novo-rapid and levemir insulin in glycemic control in gestational diabetes. Human antibodies. 2019;27(4):285-9. https://doi.org/10.3233/hab-190385.

Anderson IA, Saukila LF, Robins JMW, Akhunbay-Fudge CY, Goodden JR, Tyagi AK, et al. Factors associated with 30-day ventriculoperitoneal shunt failure in pediatric and adult patients. Journal of neurosurgery. 2018;130(1):145-53. https://doi.org/10.3171/2017.8.Jns17399.

Annuzzi G, Del Prato S, Arcari R, Bellomo Damato A, Benzi L, Bruttomesso D, et al. Preprandial combination of lispro and NPH insulin improves overall blood glucose control in type 1 diabetic patients: a multicenter randomized crossover trial. Nutrition, metabolism, and cardiovascular diseases : NMCD. 2001;11(3):168-75. https://www.researchgate.net/publication/11758907_Preprandial_combination_of_lispro_and_NPH_insulin_improves_overall_blood_glucose_control_in_type_1_diabetic_patients_A_multicenter_randomized_crossover_trial.

Arighi A, Di Cristofori A, Fenoglio C, Borsa S, D'Anca M, Fumagalli GG, et al. Cerebrospinal Fluid Level of Aquaporin4: A New Window on Glymphatic System Involvement in Neurodegenerative Disease? Journal of Alzheimer's disease : JAD. 2019;69(3):663-9. https://doi.org/10.3233/jad-190119.

Arslansoyu Camlar S, Ünlü M, Soylu A, Karaca D, Sarioglu S, Kavukcu S. Contribution of Electron Microscopy to the Clinicopathologic Diagnosis in Childhood Glomerular Renal Diseases. Fetal and pediatric pathology. 2019;38(4):299-306. https://doi.org/10.1080/15513815.2019.1587119.

Arutchelvam V, Heise T, Dellweg S, Elbroend B, Minns I, Home PD. Plasma glucose and hypoglycaemia following exercise in people with Type 1 diabetes: a comparison of three basal insulins. Diabetic medicine : a journal of the British Diabetic Association. 2009;26(10):1027-32. https://doi.org/10.1111/j.1464-5491.2009.02807.x.

Ashraf R, Sostre S. Differing scintigraphic patterns of lumboperitoneal shunt dysfunction in patients with normal pressure hydrocephalus and pseudotumor cerebri. Clinical nuclear medicine. 1995;20(2):140-6. https://doi.org/10.1097/00003072-199502000-00010.

Athanasiadou KI, Paschou SA, Stamatopoulos T, Papakonstantinou E, Haidich AB, Goulis DG. Safety and efficacy of insulin detemir versus NPH in the treatment of diabetes during pregnancy: Systematic review and meta-analysis of randomized controlled trials. Diabetes research and clinical practice. 2022;190:110020. https://doi.org/10.1016/j.diabres.2022.110020.

Baala L, Romano S, Khaddour R, Saunier S, Smith UM, Audollent S, et al. The Meckel-Gruber syndrome gene, MKS3, is mutated in Joubert syndrome. Am J Hum Genet. 2007;80(1):186-94. https://doi.org/10.1086/510499.

Bacchetta J, Chapurlat R, Bouvier R, Antignac C, Dubourg L, Kohler R, et al. Nephronophthisis-like nephritis associated with fibrous dysplasia of bone. Pediatr Nephrol. 2008;23(9):1559-63. https://doi.org/10.1007/s00467-008-0850-x.

Balikova I, Sanak NS, Fanny D, Smits G, Soblet J, de Baere E, et al. Three cases of molecularly confirmed Knobloch syndrome. Ophthalmic genetics. 2020;41(1):83-7. https://doi.org/10.1080/13816810.2020.1737948.

Barola A, Tiwari P, Bhansali A, Grover S, Dayal D. Insulin-Related Lipohypertrophy: Lipogenic Action or Tissue Trauma? Frontiers in endocrinology. 2018;9:638. https://doi.org/10.3389/fendo.2018.00638.

Barrio Castellanos R. Long-acting insulin analogues (insulin glargine or determir) and continuous subcutaneous insulin infusion in the treatment of type 1 diabetes mellitus in the paediatric population. Journal of pediatric endocrinology & metabolism : JPEM. 2005;18 Suppl 1:1173-9. https://doi.org/10.1515/jpem.2005.18.s1.1173.

Bartley PC, Bogoev M, Larsen J, Philotheou A. Long-term efficacy and safety of insulin detemir compared to Neutral Protamine Hagedorn insulin in patients with Type 1 diabetes using a treat-to-target basal-bolus regimen with insulin aspart at meals: a 2-year, randomized, controlled trial. Diabetic medicine : a journal of the British Diabetic Association. 2008;25(4):442-9. https://doi.org/10.1111/j.1464-5491.2007.02407.x.

Bateman GA. The reversibility of reduced cortical vein compliance in normal-pressure hydrocephalus following shunt insertion. Neuroradiology. 2003;45(2):65-70. https://doi.org/10.1007/s00234-002-0901-0.

Battin M, Yong C, Phang M, Daaboul J. Transient neonatal diabetes mellitus and macroglossia. Journal of perinatology : official journal of the California Perinatal Association. 1996;16(4):288-91.

Bdaiwi AS, Niedbalski PJ, Hossain MM, Willmering MM, Walkup LL, Wang H, et al. Improving hyperpolarized (129) Xe ADC mapping in pediatric and adult lungs with uncertainty propagation. NMR in biomedicine. 2022;35(3):e4639. https://doi.org/10.1002/nbm.4639.

Beltrán S, Reisert M, Krafft AJ, Frase S, Mast H, Urbach H, et al. Spinal cord motion and CSF flow in the cervical spine of 70 healthy participants. NMR in biomedicine. 2024;37(7):e5013. https://doi.org/10.1002/nbm.5013.

Berger A, Constantini S, Ram Z, Roth J. Acute subdural hematomas in shunted normal-pressure hydrocephalus patients - Management options and literature review: A case-based series. Surgical neurology international. 2018;9:238. https://doi.org/10.4103/sni.sni_338_18.

Berger M, Rodbard D. Computer simulation of plasma insulin and glucose dynamics after subcutaneous insulin injection. Diabetes care. 1989;12(10):725-36. https://doi.org/10.2337/diacare.12.10.725.

Bergmann C, Fliegauf M, Brüchle NO, Frank V, Olbrich H, Kirschner J, et al. Loss of nephrocystin-3 function can cause embryonic lethality, Meckel-Gruber-like syndrome, situs inversus, and renal-hepatic-pancreatic dysplasia. Am J Hum Genet. 2008;82(4):959-70. https://doi.org/10.1016/j.ajhg.2008.02.017.

Bernstein J. Glomerulocystic kidney disease--nosological considerations. Pediatr Nephrol. 1993;7(4):464-70. https://doi.org/10.1007/bf00857576.

Bir SC, Patra DP, Maiti TK, Sun H, Guthikonda B, Notarianni C, et al. Epidemiology of adult-onset hydrocephalus: institutional experience with 2001 patients. Neurosurgical focus. 2016;41(3):E5. https://doi.org/10.3171/2016.7.Focus16188.

Birkebæk NH, Sørensen JS, Vikre-Jørgensen J, Jensen PK, Pedersen O, Hansen T. A De Novo Whole GCK Gene Deletion Not Detected by Gene Sequencing, in a Boy with Phenotypic GCK Insufficiency. Case reports in genetics. 2011;2011:768610. https://doi.org/10.1155/2011/768610.

Bockenhauer D, van't Hoff W, Dattani M, Lehnhardt A, Subtirelu M, Hildebrandt F, et al. Secondary nephrogenic diabetes insipidus as a complication of inherited renal diseases. Nephron Physiology. 2010;116(4):p23-9. https://doi.org/10.1159/000320117.

Bohn B, Karges B, Vogel C, Otto KP, Marg W, Hofer SE, et al. 20 Years of Pediatric Benchmarking in Germany and Austria: Age-Dependent Analysis of Longitudinal Follow-Up in 63,967 Children and Adolescents with Type 1 Diabetes. PLoS One. 2016;11(8):e0160971. https://doi.org/10.1371/journal.pone.0160971.

Bolli GB. Rationale for using combinations of short-acting insulin analogue and NPH insulin at mealtime in the treatment of type 1 diabetes mellitus. Journal of pediatric endocrinology & metabolism : JPEM. 1999;12 Suppl 3:737-44.

Bolli GB, Songini M, Trovati M, Del Prato S, Ghirlanda G, Cordera R, et al. Lower fasting blood glucose, glucose variability and nocturnal hypoglycaemia with glargine vs NPH basal insulin in subjects with Type 1 diabetes. Nutrition, metabolism, and cardiovascular diseases : NMCD. 2009;19(8):571-9. https://doi.org/10.1016/j.numecd.2008.05.003.

Bollinger ME, Hamilton RG, Wood RA. Protamine allergy as a complication of insulin hypersensitivity: A case report. The Journal of allergy and clinical immunology. 1999;104(2 Pt 1):462-5. https://doi.org/10.1016/s0091-6749(99)70394-5.

Braun D, Konrad D, Lang-Muritano M, Schoenle E. Improved glycemic control and lower frequency of severe hypoglycemia with insulin detemir; long-term experience in 105 children and adolescents with type 1 diabetes. Pediatric diabetes. 2008;9(4 Pt 2):382-7. https://doi.org/10.1111/j.1399-5448.2008.00371.x.

Bredrup C, Saunier S, Oud MM, Fiskerstrand T, Hoischen A, Brackman D, et al. Ciliopathies with skeletal anomalies and renal insufficiency due to mutations in the IFT-A gene WDR19. Am J Hum Genet. 2011;89(5):634-43. https://doi.org/10.1016/j.ajhg.2011.10.001.

Bremberg SG. The Swedish intersectoral national public health policy: effects on child and adolescent health. Eur J Public Health. 2023;33(4):585-90. https://doi.org/10.1093/eurpub/ckad100.

Bret P, Chazal J. Chronic ("normal pressure") hydrocephalus in childhood and adolescence. A review of 16 cases and reappraisal of the syndrome. Child's nervous system : ChNS : official journal of the International Society for Pediatric Neurosurgery. 1995;11(12):687-91. https://doi.org/10.1007/bf00262232.

Bret P, Guyotat J, Chazal J. Is normal pressure hydrocephalus a valid concept in 2002? A reappraisal in five questions and proposal for a new designation of the syndrome as "chronic hydrocephalus". J Neurol Neurosurg Psychiatry. 2002;73(1):9-12. https://doi.org/10.1136/jnnp.73.1.9.

Brock Jacobsen I, Vind BF, Korsholm L, Flyvbjerg A, Frystyk J, Holst JJ, et al. Counter-regulatory hormone responses to spontaneous hypoglycaemia during treatment with insulin Aspart or human soluble insulin: a double-blinded randomized cross-over study. Acta physiologica (Oxford, England). 2011;202(3):337-47. https://doi.org/10.1111/j.1748-1716.2011.02307.x.

Brown A, Steel JM, Duncan C, Duncan A, McBain AM. An assessment of the adequacy of suspension of insulin in pen injectors. Diabetic medicine : a journal of the British Diabetic Association. 2004;21(6):604-8. https://doi.org/10.1111/j.1464-5491.2004.01206.x.

Bruce G, Chaudhury S, Reynolds B. Bilateral primary renal diffuse large B-cell lymphoma: a rare presentation of paediatric renal disease mimicking juvenile nephronophthisis. BMJ case reports. 2020;13(7). https://doi.org/10.1136/bcr-2020-234810.

Brunetti VC, Yu OHY, Platt RW, Filion KB. Initiation of four basal insulins and subsequent treatment modification in people treated for type 2 diabetes in the United Kingdom: Changes over the period 2003-2018. Diabetic medicine : a journal of the British Diabetic Association. 2021;38(8):e14603. https://doi.org/10.1111/dme.14603.

Brunetti VC, Yu OHY, Platt RW, Filion KB. The association of long-acting insulin analogue use versus neutral protamine Hagedorn insulin use and the risk of major adverse cardiovascular events among individuals with type 2 diabetes: A population-based cohort study. Diabetes, obesity & metabolism. 2022;24(11):2169-81. https://doi.org/10.1111/dom.14802.

Bruttomesso D, Costa S, Baritussio A. Continuous subcutaneous insulin infusion (CSII) 30 years later: still the best option for insulin therapy. Diabetes/metabolism research and reviews. 2009;25(2):99-111. https://doi.org/10.1002/dmrr.931.

Bullich G, Vargas I, Trujillano D, Mendizábal S, Piñero-Fernández JA, Fraga G, et al. Contribution of the TTC21B gene to glomerular and cystic kidney diseases. Nephrol Dial Transplant. 2017;32(1):151-6. https://doi.org/10.1093/ndt/gfv453.

Bystricky S, Szu SC. O-acetylation affects the binding properties of the carboxyl groups on the Vi bacterial polysaccharide. Biophysical chemistry. 1994;51(1):1-7. https://doi.org/10.1016/0301-4622(94)00002-6.

Cagan Appak Y, Baran M, Ozturk Hismi B, Ozyilmaz B, Vardi K, Ozer Kaya O, et al. Renal-Hepatic-Pancreatic Dysplasia: An Ultra-Rare Ciliopathy with a Novel NPHP3 Genotype. Journal of pediatric genetics. 2020;9(2):101-3. https://doi.org/10.1055/s-0039-1696974.

Campbell RK, White JR, Levien T, Baker D. Insulin glargine. Clinical therapeutics. 2001;23(12):1938-57; discussion 23. https://doi.org/10.1016/s0149-2918(01)80148-x.

Capel C, Kasprowicz M, Czosnyka M, Baledent O, Smielewski P, Pickard JD, et al. Cerebrovascular time constant in patients suffering from hydrocephalus. Neurological research. 2014;36(3):255-61. https://doi.org/10.1179/1743132813y.0000000282.

Cengiz E, Sherr JL, Erkin-Cakmak A, Weinzimer SA, Burke EN, Sikes KA, et al. A bridge to insulin pump therapy: twice-daily regimen with NPH and detemir insulins during initial treatment of youth with type 1 diabetes mellitus. Endocrine practice : official journal of the American College of Endocrinology and the American Association of Clinical Endocrinologists. 2011;17(6):862-6. https://doi.org/10.4158/ep11031.Or.

Chapman TM, Perry CM. Spotlight on insulin detemir in type 1 and 2 diabetes mellitus. BioDrugs : clinical immunotherapeutics, biopharmaceuticals and gene therapy. 2005;19(1):67-9. https://doi.org/10.2165/00063030-200519010-00008.

Chari A, Dasgupta D, Smedley A, Craven C, Dyson E, Matloob S, et al. Intraparenchymal intracranial pressure monitoring for hydrocephalus and cerebrospinal fluid disorders. Acta neurochirurgica. 2017;159(10):1967-78. https://doi.org/10.1007/s00701-017-3281-2.

Chase HP, Arslanian S, White NH, Tamborlane WV. Insulin glargine versus intermediate-acting insulin as the basal component of multiple daily injection regimens for adolescents with type 1 diabetes mellitus. J Pediatr. 2008;153(4):547-53. https://doi.org/10.1016/j.jpeds.2008.04.063.

Chen IY, Whitney-Miller CL, Liao X. Congenital hepatic fibrosis and its mimics: a clinicopathologic study of 19 cases at a single institution. Diagnostic pathology. 2021;16(1):81. https://doi.org/10.1186/s13000-021-01142-y.

Cheng J, Su W, Wang Y, Zhan Y, Wang Y, Yan S, et al. Magnetic resonance imaging based on radiomics for differentiating T1-category nasopharyngeal carcinoma from nasopharyngeal lymphoid hyperplasia: a multicenter study. Jpn J Radiol. 2024;42(7):709-19. https://doi.org/10.1007/s11604-024-01544-0.

Cherubini V, Pintaudi B, Rossi MC, Lucisano G, Pellegrini F, Chiumello G, et al. Severe hypoglycemia and ketoacidosis over one year in Italian pediatric population with type 1 diabetes mellitus: a multicenter retrospective observational study. Nutrition, metabolism, and cardiovascular diseases : NMCD. 2014;24(5):538-46. https://doi.org/10.1016/j.numecd.2013.11.004.

Choi YJ, Halbritter J, Braun DA, Schueler M, Schapiro D, Rim JH, et al. Mutations of ADAMTS9 Cause Nephronophthisis-Related Ciliopathy. Am J Hum Genet. 2019;104(1):45-54. https://doi.org/10.1016/j.ajhg.2018.11.003.

Chuang YF, Tsai TC. Sonographic findings in familial juvenile nephronophthisis-medullary cystic disease complex. Journal of clinical ultrasound : JCU. 1998;26(4):203-6. https://doi.org/10.1002/(sici)1097-0096(199805)26:4<203::aid-jcu5>3.0.co;2-h.

Clissold R, Clissold S. Insulin glargine in the management of diabetes mellitus: an evidence-based assessment of its clinical efficacy and economic value. Core evidence. 2007;2(2):89-110. https://pmc.ncbi.nlm.nih.gov/articles/PMC3012430/.

Cohen N, Minshall ME, Sharon-Nash L, Zakrzewska K, Valentine WJ, Palmer AJ. Continuous subcutaneous insulin infusion versus multiple daily injections of insulin: economic comparison in adult and adolescent type 1 diabetes mellitus in Australia. PharmacoEconomics. 2007;25(10):881-97. https://doi.org/10.2165/00019053-200725100-00006.

Colenda CC, Hamer RM. First admission young adult patients to a state hospital: relative risk for rapid readmission. The Psychiatric quarterly. 1989;60(3):227-36. https://doi.org/10.1007/bf01064798.

Copelovitch L, O'Brien MM, Guttenberg M, Otto EA, Kaplan BS. Renal-hepatic-pancreatic dysplasia: a sibship with skeletal and central nervous system anomalies and NPHP3 mutation. American journal of medical genetics Part A. 2013;161a(7):1743-9. https://doi.org/10.1002/ajmg.a.35958.

Crinó A, Schiaffini R, Ciampalini P, Suraci MC, Manfrini S, Visalli N, et al. A two year observational study of nicotinamide and intensive insulin therapy in patients with recent onset type 1 diabetes mellitus. Journal of pediatric endocrinology & metabolism : JPEM. 2005;18(8):749-54. https://doi.org/10.1515/jpem.2005.18.8.749.

Cummins E, Royle P, Snaith A, Greene A, Robertson L, McIntyre L, et al. Clinical effectiveness and cost-effectiveness of continuous subcutaneous insulin infusion for diabetes: systematic review and

economic evaluation. Health Technol Assess. 2010;14(11):iii-iv, xi-xvi, 1-181. https://doi.org/10.3310/hta14110.

Curran T, Lang AE. Parkinsonian syndromes associated with hydrocephalus: case reports, a review of the literature, and pathophysiological hypotheses. Movement disorders : official journal of the Movement Disorder Society. 1994;9(5):508-20. https://doi.org/10.1002/mds.870090503.

Czepko R, Cieslicki K. Repeated assessment of suspected normal pressure hydrocephalus in non-shunted cases. A prospective study based on the constant rate lumbar infusion test. Acta neurochirurgica. 2016;158(5):855-63; discussion 63. https://doi.org/10.1007/s00701-016-2732-5.

Czosnyka Z, Keong N, Kim DJ, Radolovich D, Smielewski P, Lavinio A, et al. Pulse amplitude of intracranial pressure waveform in hydrocephalus. Acta neurochirurgica Supplement. 2008;102:137-40. https://doi.org/10.1007/978-3-211-85578-2_28.

Danne T, Lüpke K, Walte K, Von Schuetz W, Gall MA. Insulin detemir is characterized by a consistent pharmacokinetic profile across age-groups in children, adolescents, and adults with type 1 diabetes. Diabetes care. 2003;26(11):3087-92. https://doi.org/10.2337/diacare.26.11.3087.

Danne T, Philotheou A, Goldman D, Guo X, Ping L, Cali A, et al. A randomized trial comparing the rate of hypoglycemia--assessed using continuous glucose monitoring--in 125 preschool children with type 1 diabetes treated with insulin glargine or NPH insulin (the PRESCHOOL study). Pediatric diabetes. 2013;14(8):593-601. https://doi.org/10.1111/pedi.12051.

Danne T, Philotheou A, Goldman D, Guo X, Ping L, Cali A, et al. Corrigendum to: A randomized trial comparing the rate of hypoglycemia—assessed using continuous glucose monitoring—in 125 preschool children with type 1 diabetes treated with insulin glargine or NPH insulin (the PRESCHOOL study). Pediatric diabetes. 2015;16(6):462. https://doi.org/10.1111/pedi.12207.

Danne T, Råstam J, Odendahl R, Näke A, Schimmel U, Szczepanski R, et al. Parental preference of prandial insulin aspart compared with preprandial human insulin in a basal-bolus scheme with NPH insulin in a 12-wk crossover study of preschool children with type 1 diabetes. Pediatric diabetes. 2007;8(5):278-85. https://doi.org/10.1111/j.1399-5448.2007.00261.x.

Davis A, Gulyani S, Manthripragada L, Luciano M, Moghekar A, Yasar S. Evaluation of the effect comorbid Parkinson syndrome on normal pressure hydrocephalus assessment. Clinical neurology and neurosurgery. 2021;207:106810. https://doi.org/10.1016/j.clineuro.2021.106810.

De Coninck C, Frid A, Gaspar R, Hicks D, Hirsch L, Kreugel G, et al. Results and analysis of the 2008-2009 Insulin Injection Technique Questionnaire survey. Journal of diabetes. 2010;2(3):168-79. https://doi.org/10.1111/j.1753-0407.2010.00077.x.

Dei Cas M, Paroni R, Saccardo A, Casagni E, Arnoldi S, Gambaro V, et al. A straightforward LC-MS/MS analysis to study serum profile of short and medium chain fatty acids. Journal of chromatography B, Analytical technologies in the biomedical and life sciences. 2020;1154:121982. https://doi.org/10.1016/j.jchromb.2020.121982.

Deiss D, Kordonouri O, Hartmann R, Hopfenmüller W, Lüpke K, Danne T. Treatment with insulin glargine reduces asymptomatic hypoglycemia detected by continuous subcutaneous glucose monitoring in children and adolescents with type 1 diabetes. Pediatric diabetes. 2007;8(3):157-62. https://doi.org/10.1111/j.1399-5448.2007.00252.x.

Deja G, Jarosz-Chobot P, Polanska J. The rate of improvement in metabolic control in children with diabetes mellitus type 1 on insulin glargine depends on age. Experimental and clinical endocrinology &

diabetes : official journal, German Society of Endocrinology [and] German Diabetes Association. 2007;115(10):662-8. https://doi.org/10.1055/s-2007-984444.

Della Manna T, Battistim C, Radonsky V, Savoldelli RD, Damiani D, Kok F, et al. Glibenclamide unresponsiveness in a Brazilian child with permanent neonatal diabetes mellitus and DEND syndrome due to a C166Y mutation in KCNJ11 (Kir6.2) gene. Arquivos brasileiros de endocrinologia e metabologia. 2008;52(8):1350-5. https://doi.org/10.1590/s0004-27302008000800024.

Delous M, Baala L, Salomon R, Laclef C, Vierkotten J, Tory K, et al. The ciliary gene RPGRIP1L is mutated in cerebello-oculo-renal syndrome (Joubert syndrome type B) and Meckel syndrome. Nature genetics. 2007;39(7):875-81. https://doi.org/10.1038/ng2039.

Dempsey JC, Phelps IG, Bachmann-Gagescu R, Glass IA, Tully HM, Doherty D. Mortality in Joubert syndrome. American journal of medical genetics Part A. 2017;173(5):1237-42. https://doi.org/10.1002/ajmg.a.38158.

Deren KE, Forsyth J, Abdullah O, Hsu EW, Klinge PM, Silverberg GD, et al. Low levels of amyloid-beta and its transporters in neonatal rats with and without hydrocephalus. Cerebrospinal fluid research. 2009;6:4. https://doi.org/10.1186/1743-8454-6-4.

Devi AN, Anil Kumar TR, Pillai SM, Jayakrishnan K, Kumar PG. Expression profiles of NPHP1 in the germ cells in the semen of men with male factor infertility. Andrology. 2015;3(4):685-93. https://doi.org/10.1111/andr.12062.

Dewan MC, Rattani A, Mekary R, Glancz LJ, Yunusa I, Baticulon RE, et al. Global hydrocephalus epidemiology and incidence: systematic review and meta-analysis. Journal of neurosurgery. 2018:1-15. https://doi.org/10.3171/2017.10.Jns17439.

Di Rocco M, Picco P, Arslanian A, Restagno G, Perfumo F, Buoncompagni A, et al. Retinitis pigmentosa, hypopituitarism, nephronophthisis, and mild skeletal dysplasia (RHYNS): a new syndrome? American journal of medical genetics. 1997;73(1):1-4. https://doi.org/10.1002/(sici)1096-8628(19971128)73:1<1::aid-ajmg1>3.0.co;2-y.

Dixon B, Peter Chase H, Burdick J, Fiallo-Scharer R, Walravens P, Klingensmith G, et al. Use of insulin glargine in children under age 6 with type 1 diabetes. Pediatric diabetes. 2005;6(3):150-4. https://doi.org/10.1111/j.1399-543X.2005.00115.x.

Dongping X, Rengui G, Yangming H, Zan H, Hua X. Neighborhood effects on the health of elderly persons: evidence from China. BMC Geriatr. 2023;23(1):879. https://doi.org/10.1186/s12877-023-04609-3.

Donn SM, Fanaroff JM. Medico-legal implications of hypothermic neuroprotection in the newborn. Journal of neonatal-perinatal medicine. 2018;11(2):109-14. https://doi.org/10.3233/npm-181792.

Dora JM, Scheffel RS. Theoretical pharmacokinetic advantages and methodological flaws: glargine is not superior to NPH insulin in children with type 1 diabetes mellitus. Arquivos brasileiros de endocrinologia e metabologia. 2010;54(1):81-3. https://doi.org/10.1590/s0004-27302010000100014.

Droste DW, Krauss JK, Berger W, Schuler E, Brown MM. Rhythmic oscillations with a wavelength of 0.5-2 min in transcranial Doppler recordings. Acta neurologica Scandinavica. 1994;90(2):99-104. https://doi.org/10.1111/j.1600-0404.1994.tb02687.x.

Duchateau J, Schreyen H, Dorchy H. Intermediate and long-acting insulin preparations without protamine sulphate are complement activators in vitro. Diabete & metabolisme. 1992;18(4):272-6. https://www.researchgate.net/publication/21697033_Intermediate_and_long-acting_insulin_preparations_without_protamine_sulphate_are_complement_activators_in_vitro.

Dündar BN, Dündar N, Eren E. Comparison of the efficacy and safety of insulin glargine and insulin detemir with NPH insulin in children and adolescents with type 1 diabetes mellitus receiving intensive insulin therapy. Journal of clinical research in pediatric endocrinology. 2009;1(4):181-7. https://doi.org/10.4274/jcrpe.v1i4.56.

Dunn CJ, Plosker GL, Keating GM, McKeage K, Scott LJ. Insulin glargine: an updated review of its use in the management of diabetes mellitus. Drugs. 2003;63(16):1743-78. https://doi.org/10.2165/00003495-200363160-00007.

Eapen SS, Connor EL, Gern JE. Insulin desensitization with insulin lispro and an insulin pump in a 5-year-old child. Annals of allergy, asthma & immunology : official publication of the American College of Allergy, Asthma, & Immunology. 2000;85(5):395-7. https://doi.org/10.1016/s1081-1206(10)62554-9.

Ebeling P, Jansson PA, Smith U, Lalli C, Bolli GB, Koivisto VA. Strategies toward improved control during insulin lispro therapy in IDDM. Importance of basal insulin. Diabetes care. 1997;20(8):1287-9. https://doi.org/10.2337/diacare.20.8.1287.

Ekoe T, Bianpambe OI, Nguefack F, Pondi DM, Kana-Sop MM, Hays NP, et al. Efficacy of an iron-fortified infant cereal to reduce the risk of iron deficiency anemia in young children in East Cameroon. Food science & nutrition. 2020;8(7):3566-77. https://doi.org/10.1002/fsn3.1639.

Ekström K, Salemyr J, Zachrisson I, Carlsson-Skwirut C, Ortqvist E, Bang P. Normalization of the IGF-IGFBP axis by sustained nightly insulinization in type 1 diabetes. Diabetes care. 2007;30(6):1357-63. https://doi.org/10.2337/dc06-2328.

El-Hawary A, Salem N, Elsharkawy A, Metwali A, Wafa A, Chalaby N, et al. Safety and metabolic impact of Ramadan fasting in children and adolescents with type 1 diabetes. Journal of pediatric endocrinology & metabolism : JPEM. 2016;29(5):533-41. https://doi.org/10.1515/jpem-2015-0263.

Elfving K, Shakely D, Andersson M, Baltzell K, Ali AS, Bachelard M, et al. Acute Uncomplicated Febrile Illness in Children Aged 2-59 months in Zanzibar - Aetiologies, Antibiotic Treatment and Outcome. PLoS One. 2016;11(1):e0146054. https://doi.org/10.1371/journal.pone.0146054.

Emmett PM, Hays NP, Taylor CM. Factors Associated with Maternal Worry about Her Young Child Exhibiting Choosy Feeding Behaviour. Int J Environ Res Public Health. 2018;15(6). https://doi.org/10.3390/ijerph15061236.

Enoksson F, Ruiz Rodriguez A, Peno C, Balcazar Lopez C, Tjernström F, Bogaert D, et al. Niche- and Gender-Dependent Immune Reactions in Relation to the Microbiota Profile in Pediatric Patients with Otitis Media with Effusion. Infection and immunity. 2020;88(10). https://doi.org/10.1128/iai.00147-20.

Estrada-Cuzcano A, Koenekoop RK, Coppieters F, Kohl S, Lopez I, Collin RW, et al. IQCB1 mutations in patients with leber congenital amaurosis. Investigative ophthalmology & visual science. 2011;52(2):834-9. https://doi.org/10.1167/iovs.10-5221.

Farber SH, Parker SL, Adogwa O, Rigamonti D, McGirt MJ. Cost analysis of antibiotic-impregnated catheters in the treatment of hydrocephalus in adult patients. World neurosurgery. 2010;74(4-5):528-31. https://doi.org/10.1016/j.wneu.2010.07.014.

Feleder EC, Yerino GA, Halabe EK, Tombazzi JL, Farias JM. Phase IV study comparing diurnal glycemic profile following the administration of 2 NPH plus regular human DNA recombinant insulin regimens in type 1 diabetes mellitus (T1DM) adult patients. Arzneimittel-Forschung. 2012;62(6):267-73. https://doi.org/10.1055/s-0032-1306274.

Fila M, Morinière V, Eckart P, Terzic J, Gubler MC, Antignac C, et al. Bi-allelic mutations in renin-angiotensin system genes, associated with renal tubular dysgenesis, can also present as a progressive

chronic kidney disease. Pediatr Nephrol. 2020;35(6):1125-8. https://doi.org/10.1007/s00467-020-04524-4.

Filler G, Grygas R, Mai I, Stolpe HJ, Greiner C, Bauer S, et al. Pharmacokinetics of tacrolimus (FK 506) in children and adolescents with renal transplants. Nephrol Dial Transplant. 1997;12(8):1668-71. https://doi.org/10.1093/ndt/12.8.1668.

Fishel Bartal M, Ward C, Refuerzo JS, Ashimi SS, Joycelyn CA, Chen HY, et al. Basal Insulin Analogs versus Neutral Protamine Hagedorn for Type 2 Diabetics. American journal of perinatology. 2020;37(1):30-6. https://doi.org/10.1055/s-0039-1694733.

Fleming LR, Doherty DA, Parisi MA, Glass IA, Bryant J, Fischer R, et al. Prospective Evaluation of Kidney Disease in Joubert Syndrome. Clin J Am Soc Nephrol. 2017;12(12):1962-73. https://doi.org/10.2215/cjn.05660517.

Flyman S, Hermansson A, Gisselsson-Solén M. Nasopharyngeal cultures in children; when, what and why? International journal of pediatric otorhinolaryngology. 2020;130:109832. https://doi.org/10.1016/j.ijporl.2019.109832.

Flynn TJ, Cadzow M, Dalbeth N, Jones PB, Stamp LK, Hindmarsh JH, et al. Positive association of tomato consumption with serum urate: support for tomato consumption as an anecdotal trigger of gout flares. BMC Musculoskelet Disord. 2015;16:196. https://doi.org/10.1186/s12891-015-0661-8.

Forner Giner J, Sanz-Requena R, Flórez N, Alberich-Bayarri A, García-Martí G, Ponz A, et al. Quantitative phase-contrast MRI study of cerebrospinal fluid flow: a method for identifying patients with normal-pressure hydrocephalus. Neurologia (Barcelona, Spain). 2014;29(2):68-75. https://doi.org/10.1016/j.nrl.2013.02.016.

Fox LA, Buckloh LM, Smith SD, Wysocki T, Mauras N. A randomized controlled trial of insulin pump therapy in young children with type 1 diabetes. Diabetes care. 2005;28(6):1277-81. https://doi.org/10.2337/diacare.28.6.1277.

Fredheim S, Johansen A, Thorsen SU, Kremke B, Nielsen LB, Olsen BS, et al. Nationwide reduction in the frequency of severe hypoglycemia by half. Acta diabetologica. 2015;52(3):591-9. https://doi.org/10.1007/s00592-014-0697-5.

Fröhlich-Reiterer EE, Ong KK, Regan F, Salzano G, Acerini CL, Dunger DB. A randomized cross-over trial to identify the optimal use of insulin glargine in prepubertal children using a three-times daily insulin regimen. Diabetic medicine : a journal of the British Diabetic Association. 2007;24(12):1406-11. https://doi.org/10.1111/j.1464-5491.2007.02277.x.

Fuchshuber A, Deltas CC, Berthold S, Stavrou C, Vollmer M, Burton C, et al. Autosomal dominant medullary cystic kidney disease: evidence of gene locus heterogeneity. Nephrol Dial Transplant. 1998;13(8):1955-7. https://doi.org/10.1093/ndt/13.8.1955.

Fukuhara T, Luciano MG. Clinical features of late-onset idiopathic aqueductal stenosis. Surgical neurology. 2001;55(3):132-6; discussion 6-7. https://doi.org/10.1016/s0090-3019(01)00359-7.

Fulcher GR, Gilbert RE, Yue DK. Glargine is superior to neutral protamine Hagedorn for improving glycated haemoglobin and fasting blood glucose levels during intensive insulin therapy. Internal medicine journal. 2005;35(9):536-42. https://doi.org/10.1111/j.1445-5994.2005.00902.x.

Gaither JB, Stolz U, Ennis J, Moiser J, Sakles JC. Association Between Difficult Airway Predictors and Failed Prehosptial Endotracheal Intubation. Air medical journal. 2015;34(6):343-7. https://doi.org/10.1016/j.amj.2015.06.001.

Gale EA. A randomized, controlled trial comparing insulin lispro with human soluble insulin in patients with Type 1 diabetes on intensified insulin therapy. The UK Trial Group. Diabetic medicine : a journal of the British Diabetic Association. 2000;17(3):209-14. https://doi.org/10.1046/j.1464-5491.2000.00258.x.

Garg S, Moser E, Dain MP, Rodionova A. Clinical experience with insulin glargine in type 1 diabetes. Diabetes technology & therapeutics. 2010;12(11):835-46. https://doi.org/10.1089/dia.2010.0135.

Gee HY, Otto EA, Hurd TW, Ashraf S, Chaki M, Cluckey A, et al. Whole-exome resequencing distinguishes cystic kidney diseases from phenocopies in renal ciliopathies. Kidney Int. 2014;85(4):880-7. https://doi.org/10.1038/ki.2013.450.

Georges B, Cosyns JP, Dahan K, Snyers B, Carlier B, Loute G, et al. Late-onset renal failure in Senior-Loken syndrome. Am J Kidney Dis. 2000;36(6):1271-5. https://doi.org/10.1053/ajkd.2000.19845.

Gerich J, Becker RH, Zhu R, Bolli GB. Fluctuation of serum basal insulin levels following single and multiple dosing of insulin glargine. Diabetes technology & therapeutics. 2006;8(2):237-43. https://doi.org/10.1089/dia.2006.8.237.

Gholampour S, Bahmani M, Shariati A. Comparing the Efficiency of Two Treatment Methods of Hydrocephalus: Shunt Implantation and Endoscopic Third Ventriculostomy. Basic and clinical neuroscience. 2019;10(3):185-98. https://doi.org/10.32598/bcn.9.10.285.

Gleason PL, Black PM, Matsumae M. The neurobiology of normal pressure hydrocephalus. Neurosurgery clinics of North America. 1993;4(4):667-75. https://doi.org/10.1016/S1042-3680(18)30558-8.

Gole E, Oikonomou S, Ellard S, De Franco E, Karavanaki K. A Novel KCNJ11 Mutation Associated with Transient Neonatal Diabetes. Journal of clinical research in pediatric endocrinology. 2018;10(2):175-8. https://doi.org/10.4274/jcrpe.5166.

Gordon J, Pockett RD, Tetlow AP, McEwan P, Home PD. A comparison of intermediate and long-acting insulins in people with type 2 diabetes starting insulin: an observational database study. International journal of clinical practice. 2010;64(12):1609-18. https://doi.org/10.1111/j.1742-1241.2010.02520.x.

Gottschlich GM, Gravlee GP, Georgitis JW. Adverse reactions to protamine sulfate during cardiac surgery in diabetic and non-diabetic patients. Annals of allergy. 1988;61(4):277-81.

Goykhman S, Drincic A, Desmangles JC, Rendell M. Insulin Glargine: a review 8 years after its introduction. Expert opinion on pharmacotherapy. 2009;10(4):705-18. https://doi.org/10.1517/14656560902775677.

Granström G, Askelöf P, Granström M. Specific immunoglobulin A to Bordetella pertussis antigens in mucosal secretion for rapid diagnosis of whooping cough. Journal of clinical microbiology. 1988;26(5):869-74. https://doi.org/10.1128/jcm.26.5.869-874.1988.

Graziadio C, Bernardi P, Rosa RF, Zen PR, Paskulin GA. Type 1 diabetes in a patient with Ellis-van Creveld syndrome. Sao Paulo Med J. 2012;130(1):53-6. https://doi.org/10.1590/s1516-31802012000100009.

Gregory R, Tattersall RB. Bovine and human NPH insulins as T cell immunogens. Diabetes research and clinical practice. 1993;20(2):139-46. https://doi.org/10.1016/0168-8227(93)90008-s.

Greuter L, Schenker T, Guzman R, Soleman J. Endoscopic third ventriculostomy compared to ventriculoperitoneal shunt as treatment for idiopathic normal pressure hydrocephalus: a systematic review and meta-analysis. British journal of neurosurgery. 2022:1-7. https://doi.org/10.1080/02688697.2022.2149697.

Griffen SC, Oostema K, Stanhope KL, Graham J, Styne DM, Glaser N, et al. Administration of Lispro insulin with meals improves glycemic control, increases circulating leptin, and suppresses ghrelin, compared with regular/NPH insulin in female patients with type 1 diabetes. The Journal of clinical endocrinology and metabolism. 2006;91(2):485-91. https://doi.org/10.1210/jc.2005-1338.

Grover P, Thomas W, Moran A. Glargine versus NPH insulin in cystic fibrosis related diabetes. Journal of cystic fibrosis : official journal of the European Cystic Fibrosis Society. 2008;7(2):134-6. https://doi.org/10.1016/j.jcf.2007.07.004.

Gschwend MH, Aagren M, Valentine WJ. Cost-effectiveness of insulin detemir compared with neutral protamine Hagedorn insulin in patients with type 1 diabetes using a basal-bolus regimen in five European countries. Journal of medical economics. 2009;12(2):114-23. https://doi.org/10.3111/13696990903080344.

Gundgaard J, Christensen TE, Thomsen TL. Direct healthcare costs of patients with type 2 diabetes using long-acting insulin analogues or NPH insulin in a basal insulin-only regimen. Primary care diabetes. 2010;4(3):165-72. https://doi.org/10.1016/j.pcd.2010.04.004.

Habel LA, Danforth KN, Quesenberry CP, Capra A, Van Den Eeden SK, Weiss NS, et al. Cohort study of insulin glargine and risk of breast, prostate, and colorectal cancer among patients with diabetes. Diabetes care. 2013;36(12):3953-60. https://doi.org/10.2337/dc13-0140.

Hakim R, Black PM. Correlation between lumbo-ventricular perfusion and MRI-CSF flow studies in idiopathic normal pressure hydrocephalus. Surgical neurology. 1998;49(1):14-9; discussion 9-20. https://doi.org/10.1016/s0090-3019(97)00032-3.

Halbritter J, Bizet AA, Schmidts M, Porath JD, Braun DA, Gee HY, et al. Defects in the IFT-B component IFT172 cause Jeune and Mainzer-Saldino syndromes in humans. Am J Hum Genet. 2013;93(5):915-25. https://doi.org/10.1016/j.ajhg.2013.09.012.

Hamarat Y, Bartusis L, Deimantavicius M, Lucinskas P, Siaudvytyte L, Zakelis R, et al. Can the Treatment of Normal-Pressure Hydrocephalus Induce Normal-Tension Glaucoma? A Narrative Review of a Current Knowledge. Medicina (Kaunas, Lithuania). 2021;57(3). https://doi.org/10.3390/medicina57030234.

Hartemann-Heurtier A, Halbron M, Golmard JL, Jacqueminet S, Bastard JP, Rouault C, et al. Effects of bed-time insulin versus pioglitazone on abdominal fat accumulation, inflammation and gene expression in adipose tissue in patients with type 2 diabetes. Diabetes research and clinical practice. 2009;86(1):37-43. https://doi.org/10.1016/j.diabres.2009.06.028.

Hassan K, Rodriguez LM, Johnson SE, Tadlock S, Heptulla RA. A randomized, controlled trial comparing twice-a-day insulin glargine mixed with rapid-acting insulin analogs versus standard neutral protamine Hagedorn (NPH) therapy in newly diagnosed type 1 diabetes. Pediatrics. 2008;121(3):e466-72. https://doi.org/10.1542/peds.2007-1679.

Hassan MM, Arafa N, Abdou M, Hussein O. Characteristics of diabetes diagnosis and control in toddlers and preschoolers from families with limited resources: A single center experience. Diabetes research and clinical practice. 2020;159:107966. https://doi.org/10.1016/j.diabres.2019.107966.

Hecht H, Ohlsson J, Starck SA. Poor renal uptake of 99mtechnetium-dimercaptosuccinic acid and near-normal 99mtechnetium-mercaptoacetyltriglycine renogram in nephronophthisis. Pediatr Nephrol. 1996;10(2):167-70. https://doi.org/10.1007/s004670050088.

Hedera P, Gorski JL. Retinitis pigmentosa, growth hormone deficiency, and acromelic skeletal dysplasia in two brothers: possible familial RHYNS syndrome. American journal of medical genetics. 2001;101(2):142-5. https://doi.org/10.1002/ajmg.1338.

Heller S, Bode B, Kozlovski P, Svendsen AL. Meta-analysis of insulin aspart versus regular human insulin used in a basal-bolus regimen for the treatment of diabetes mellitus. Journal of diabetes. 2013;5(4):482-91. https://doi.org/10.1111/1753-0407.12060.

Heller S, Damm P, Mersebach H, Skjøth TV, Kaaja R, Hod M, et al. Hypoglycemia in type 1 diabetic pregnancy: role of preconception insulin aspart treatment in a randomized study. Diabetes care. 2010;33(3):473-7. https://doi.org/10.2337/dc09-1605.

Heller SR, Colagiuri S, Vaaler S, Wolffenbuttel BH, Koelendorf K, Friberg HH, et al. Hypoglycaemia with insulin aspart: a double-blind, randomised, crossover trial in subjects with Type 1 diabetes. Diabetic medicine : a journal of the British Diabetic Association. 2004;21(7):769-75. https://doi.org/10.1111/j.1464-5491.2004.01244.x.

Hemmingsen B, Metzendorf MI, Richter B. (Ultra-)long-acting insulin analogues for people with type 1 diabetes mellitus. The Cochrane database of systematic reviews. 2021;3(3):Cd013498. https://doi.org/10.1002/14651858.CD013498.pub2.

Hermansen K, Madsbad S, Perrild H, Kristensen A, Axelsen M. Comparison of the soluble basal insulin analog insulin detemir with NPH insulin: a randomized open crossover trial in type 1 diabetic subjects on basal-bolus therapy. Diabetes care. 2001;24(2):296-301. https://doi.org/10.2337/diacare.24.2.296.

Herrera KM, Rosenn BM, Foroutan J, Bimson BE, Al Ibraheemi Z, Moshier EL, et al. Randomized controlled trial of insulin detemir versus NPH for the treatment of pregnant women with diabetes. American journal of obstetrics and gynecology. 2015;213(3):426.e1-7. https://doi.org/10.1016/j.ajog.2015.06.010.

Hershon KS, Blevins TC, Mayo CA, Rosskamp R. Once-daily insulin glargine compared with twice-daily NPH insulin in patients with type 1 diabetes. Endocrine practice : official journal of the American College of Endocrinology and the American Association of Clinical Endocrinologists. 2004;10(1):10-7. https://doi.org/10.4158/ep.10.1.10.

Herwig J, Scholl-Schilling G, Böhles H. Glycaemic control and hypoglycaemia in children, adolescents and young adults with unstable type 1 diabetes mellitus treated with insulin glargine or intermediate-acting insulin. Journal of pediatric endocrinology & metabolism : JPEM. 2007;20(4):517-25. https://doi.org/10.1515/jpem.2007.20.4.517.

Hildebrandt F, Nothwang HG, Vossmerbäumer U, Springer C, Strahm B, Hoppe B, et al. Lack of large, homozygous deletions of the nephronophthisis 1 region in Joubert syndrome type B. APN Study Group. Arbeitsgemeinschaft für Pädiatrische Nephrologie. Pediatr Nephrol. 1998;12(1):16-9. https://doi.org/10.1007/s004670050394.

Hirabayashi S, Shigematsu H, Iai M, Takashima S. A neurodegenerative disorder with early myoclonic encephalopathy, retinal pigmentary degeneration and nephronophthisis. Brain & development. 2000;22(1):24-30. https://doi.org/10.1016/s0387-7604(99)00085-6.

Hirigo AT, Gutema S, Eifa A, Ketema W. Experience of dolutegravir-based antiretroviral treatment and risks of diabetes mellitus. SAGE open medical case reports. 2022;10:2050313x221079444. https://doi.org/10.1177/2050313x221079444.

Hissa MN, Hissa AS, Bruin VM, Fredrickson LP. Comparison between continuous subcutaneous insulin infusion and multiple insulin injection therapy in type 1 diabetes mellitus: 18-month follow-up. Endocrine

practice : official journal of the American College of Endocrinology and the American Association of Clinical Endocrinologists. 2002;8(6):411-6. https://doi.org/10.4158/ep.8.6.411.

Hiyama E, Hishiki T, Watanabe K, Ida K, Yano M, Oue T, et al. Mortality and morbidity in primarily resected hepatoblastomas in Japan: Experience of the JPLT (Japanese Study Group for Pediatric Liver Tumor) trials. Journal of pediatric surgery. 2015;50(12):2098-101. https://doi.org/10.1016/j.jpedsurg.2015.08.035.

Hod M, Mathiesen ER, Jovanovič L, McCance DR, Ivanisevic M, Durán-Garcia S, et al. A randomized trial comparing perinatal outcomes using insulin detemir or neutral protamine Hagedorn in type 1 diabetes. The journal of maternal-fetal & neonatal medicine : the official journal of the European Association of Perinatal Medicine, the Federation of Asia and Oceania Perinatal Societies, the International Society of Perinatal Obstet. 2014;27(1):7-13. https://doi.org/10.3109/14767058.2013.799650.

Holl RW, Teller WM, Heinze E. Semilente-insulin at bedtime is superior to NPH-insulin for the suppression of the dawn-phenomenon in adolescents with type-I-diabetes. Experimental and clinical endocrinology & diabetes : official journal, German Society of Endocrinology [and] German Diabetes Association. 1996;104(5):360-4. https://doi.org/10.1055/s-0029-1211468.

Holmberg H, Mersebach H, Kanc K, Ludvigsson J. Antibody response to insulin in children and adolescents with newly diagnosed Type 1 diabetes. Diabetic medicine : a journal of the British Diabetic Association. 2008;25(7):792-7. https://doi.org/10.1111/j.1464-5491.2008.02468.x.

Home PD, Lagarenne P. Combined randomised controlled trial experience of malignancies in studies using insulin glargine. Diabetologia. 2009;52(12):2499-506. https://doi.org/10.1007/s00125-009-1530-5.

Home PD, Rosskamp R, Forjanic-Klapproth J, Dressler A. A randomized multicentre trial of insulin glargine compared with NPH insulin in people with type 1 diabetes. Diabetes/metabolism research and reviews. 2005;21(6):545-53. https://doi.org/10.1002/dmrr.572.

Hong R, Sok T. Retrospective study of empyema cases in National Pediatric Hospital, Cambodia. The Southeast Asian journal of tropical medicine and public health. 1997;28(4):801-2. https://www.tm.mahidol.ac.th/seameo/1997-28-4/1997-28-4-801.pdf.

Hsia SH. Insulin glargine compared to NPH among insulin-naïve, U.S. inner city, ethnic minority type 2 diabetic patients. Diabetes research and clinical practice. 2011;91(3):293-9. https://doi.org/10.1016/j.diabres.2010.11.028.

Hung CT, Wang FF. Pen injector for insulin-requiring diabetic patients. Journal of the Formosan Medical Association = Taiwan yi zhi. 1992;91(10):1026-9.

Huynh Cong E, Bizet AA, Boyer O, Woerner S, Gribouval O, Filhol E, et al. A homozygous missense mutation in the ciliary gene TTC21B causes familial FSGS. Journal of the American Society of Nephrology : JASN. 2014;25(11):2435-43. https://doi.org/10.1681/asn.2013101126.

Iafusco D. Insulin therapy regimens in paediatric age. Acta bio-medica : Atenei Parmensis. 2005;76 Suppl 3:39-43. https://www.researchgate.net/publication/6871707_Insulin_therapy_regimens_in_paediatric_age.

Insolera R, Shao W, Airik R, Hildebrandt F, Shi SH. SDCCAG8 regulates pericentriolar material recruitment and neuronal migration in the developing cortex. Neuron. 2014;83(4):805-22. https://doi.org/10.1016/j.neuron.2014.06.029.

Iorember FM, Vehaskari VM. Uromodulin: old friend with new roles in health and disease. Pediatr Nephrol. 2014;29(7):1151-8. https://doi.org/10.1007/s00467-013-2563-z.

Jacobs MA, Keulen ET, Kanc K, Casteleijn S, Scheffer P, Devillé W, et al. Metabolic efficacy of preprandial administration of Lys(B28), Pro(B29) human insulin analog in IDDM patients. A comparison with human regular insulin during a three-meal test period. Diabetes care. 1997;20(8):1279-86. https://doi.org/10.2337/diacare.20.8.1279.

Janik JE, Bloch CA, Janik JS. Intrathyroid parathyroid gland and neonatal primary hyperparathyroidism. Journal of pediatric surgery. 2000;35(10):1517-9. https://doi.org/10.1053/jpsu.2000.16430.

Jean-Baptiste E, Larco P, von Oettingen J, Ogle GD, Moïse K, Fleury-Milfort E, et al. Efficacy of a New Protocol of Premixed 70/30 Human Insulin in Haitian Youth with Diabetes. Diabetes therapy : research, treatment and education of diabetes and related disorders. 2021;12(9):2545-56. https://doi.org/10.1007/s13300-021-01130-x.

Jennings T, Perry TE, Valeriani J. In The Best Interest Of The (Adult) Child: Ideas About Kinship Care Of Older Adults. Journal of family social work. 2014;17(1):35-50. https://doi.org/10.1080/10522158.2013.865289.

Ji J, He Z, Yang Z, Mi Y, Guo N, Zhao H, et al. Comparing the efficacy and safety of insulin detemir versus neutral protamine hagedorn insulin in treatment of diabetes during pregnancy: a randomized, controlled study. BMJ open diabetes research & care. 2020;8(1). https://doi.org/10.1136/bmjdrc-2019-001155.

Jones LR, Emmett PM, Hays NP, Shahkhalili Y, Taylor CM. Association of Nutrition in Early Childhood with Body Composition and Leptin in Later Childhood and Early Adulthood. Nutrients. 2021;13(9). https://doi.org/10.3390/nu13093264.

Jovanovic L. Achieving euglycaemia in women with gestational diabetes mellitus: current options for screening, diagnosis and treatment. Drugs. 2004;64(13):1401-17. https://doi.org/10.2165/00003495-200464130-00002.

Kadiri A, Al-Nakhi A, El-Ghazali S, Jabbar A, Al Arouj M, Akram J, et al. Treatment of type 1 diabetes with insulin lispro during Ramadan. Diabetes & metabolism. 2001;27(4 Pt 1):482-6. https://www.researchgate.net/publication/11800516_Treatment_of_Type-1_diabetes_with_lispro_during_Ramadan.

Kaestner S, Poetschke M, Roth C, Deinsberger W. Different origins of hydrocephalus lead to different shunt revision rates. Neurologia i neurochirurgia polska. 2017;51(1):72-6. https://doi.org/10.1016/j.pjnns.2016.11.007.

Kageyama H, Miyajima M, Ogino I, Nakajima M, Shimoji K, Fukai R, et al. Panventriculomegaly with a wide foramen of Magendie and large cisterna magna. Journal of neurosurgery. 2016;124(6):1858-66. https://doi.org/10.3171/2015.6.Jns15162.

Kaiserman K, Jung H, Benabbad I, Karges B, Polak M, Rosilio M. 20 Years of insulin lispro in pediatric type 1 diabetes: a review of available evidence. Pediatric diabetes. 2017;18(2):81-94. https://doi.org/10.1111/pedi.12401.

Kane RE, Black P. Glucose intolerance with low-, medium-, and high-carbohydrate formulas during nighttime enteral feedings in cystic fibrosis patients. Journal of pediatric gastroenterology and nutrition. 1989;8(3):321-6. https://doi.org/10.1097/00005176-198904000-00010.

Kang HG, Lee HK, Ahn YH, Joung JG, Nam J, Kim NK, et al. Targeted exome sequencing resolves allelic and the genetic heterogeneity in the genetic diagnosis of nephronophthisis-related ciliopathy. Experimental & molecular medicine. 2016;48(8):e251. https://doi.org/10.1038/emm.2016.63.

Kang YS, Park EK, Kim JS, Kim DS, Thomale UW, Shim KW. Efficacy of endoscopic third ventriculostomy in old aged patients with normal pressure hydrocephalus. Neurologia i neurochirurgia polska. 2018;52(1):29-34. https://doi.org/10.1016/j.pjnns.2017.10.004.

Kapellen TM, Wolf J, Rosenbauer J, Stachow R, Ziegler R, Szczepanski R, et al. Changes in the use of analogue insulins in 37 206 children and adolescents with type 1 diabetes in 275 German and Austrian centers during the last twelve years. Experimental and clinical endocrinology & diabetes : official journal, German Society of Endocrinology [and] German Diabetes Association. 2009;117(7):329-35. https://doi.org/10.1055/s-0028-1103289.

Karagüzel G, Satilmiş A, Akçurin S, Bircan I. Comparison of breakfast and bedtime administration of insulin glargine in children and adolescents with Type 1 diabetes. Diabetes research and clinical practice. 2006;74(1):15-20. https://doi.org/10.1016/j.diabres.2006.03.007.

Karges B, Boehm BO, Karges W. Early hypoglycaemia after accidental intramuscular injection of insulin glargine. Diabetic medicine : a journal of the British Diabetic Association. 2005;22(10):1444-5. https://doi.org/10.1111/j.1464-5491.2005.01654.x.

Karges B, Kapellen T, Neu A, Hofer SE, Rohrer T, Rosenbauer J, et al. Long-acting insulin analogs and the risk of diabetic ketoacidosis in children and adolescents with type 1 diabetes: a prospective study of 10,682 patients from 271 institutions. Diabetes care. 2010;33(5):1031-3. https://doi.org/10.2337/dc09-2249.

Karimova ED, Gulyaeva AS, Katermin NS. The degree of mu rhythm suppression in women is associated with presence of children as well as empathy and anxiety level. Social neuroscience. 2022;17(4):382-96. https://doi.org/10.1080/17470919.2022.2112753.

Karimova ED, Ovakimian AS, Katermin NS. Live vs video interaction: sensorimotor and visual cortical oscillations during action observation. Cereb Cortex. 2024;34(4). https://doi.org/10.1093/cercor/bhae168.

Katagiri S, Hayashi T, Yoshitake K, Murai N, Matsui Z, Kubo H, et al. Compound heterozygous splice site variants in the SCLT1 gene highlight an additional candidate locus for Senior-Løken syndrome. Sci Rep. 2018;8(1):16733. https://doi.org/10.1038/s41598-018-35152-6.

Katayama S, Asari S, Ohmoto T. Quantitative measurement of normal and hydrocephalic cerebrospinal fluid flow using phase contrast cine MR imaging. Acta medica Okayama. 1993;47(3):157-68. https://doi.org/10.18926/amo/31595.

Katsuragi S, Teraoka K, Ikegami K, Amano K, Yamashita K, Ishizuka K, et al. Late onset X-linked hydrocephalus with normal cerebrospinal fluid pressure. Psychiatry and clinical neurosciences. 2000;54(4):487-92. https://doi.org/10.1046/j.1440-1819.2000.00740.x.

Katz ML, Volkening LK, Anderson BJ, Laffel LM. Contemporary rates of severe hypoglycaemia in youth with type 1 diabetes: variability by insulin regimen. Diabetic medicine : a journal of the British Diabetic Association. 2012;29(7):926-32. https://doi.org/10.1111/j.1464-5491.2012.03646.x.

Kaufman FR, Halvorson M, Kim C, Pitukcheewanont P. Use of insulin pump therapy at nighttime only for children 7-10 years of age with type 1 diabetes. Diabetes care. 2000;23(5):579-82. https://doi.org/10.2337/diacare.23.5.579.

Keating GM. Insulin detemir: a review of its use in the management of diabetes mellitus. Drugs. 2012;72(17):2255-87. https://doi.org/10.2165/11470200-000000000-00000.

Kendler KS, Sheth K, Gardner CO, Prescott CA. Childhood parental loss and risk for first-onset of major depression and alcohol dependence: the time-decay of risk and sex differences. Psychological medicine. 2002;32(7):1187-94. https://doi.org/10.1017/s0033291702006219.

Kent DJ, McMahill-Walraven CN, Panozzo CA, Pawloski PA, Haynes K, Marshall J, et al. Descriptive Analysis of Long- and Intermediate-Acting Insulin and Key Safety Outcomes in Adults with Type 2 Diabetes Mellitus. Journal of managed care & specialty pharmacy. 2019;25(11):1162-71. https://doi.org/10.18553/jmcp.2019.19042.

Kerouz N, el-Hayek R, Langhough R, MacDonald MJ. Insulin doses in children using conventional therapy for insulin dependent diabetes. Diabetes research and clinical practice. 1995;29(2):113-20. https://doi.org/10.1016/0168-8227(95)01122-6.

Khadilkar VV, Khadilkar AV. Concomitant use of insulin glargine and NPH in type I diabetes. Indian pediatrics. 2005;42(8):796-800. https://www.indianpediatrics.net/aug2005/796.pdf.

Khan TN, Khan K, Sadeghpour A, Reynolds H, Perilla Y, McDonald MT, et al. Mutations in NCAPG2 Cause a Severe Neurodevelopmental Syndrome that Expands the Phenotypic Spectrum of Condensinopathies. Am J Hum Genet. 2019;104(1):94-111. https://doi.org/10.1016/j.ajhg.2018.11.017.

Khanna P, Maron JL, Walt DR. Development of a Rapid Salivary Proteomic Platform for Oral Feeding Readiness in the Preterm Newborn. Front Pediatr. 2017;5:268. https://doi.org/10.3389/fped.2017.00268.

Kilburn KH, Warshaw RH. Effects on neurobehavioral performance of chronic exposure to chemically contaminated well water. Toxicology and industrial health. 1993;9(3):391-404. https://doi.org/10.1177/074823379300900301.

Kim DS, Choi JU, Huh R, Yun PH, Kim DI. Quantitative assessment of cerebrospinal fluid hydrodynamics using a phase-contrast cine MR image in hydrocephalus. Child's nervous system : ChNS : official journal of the International Society for Pediatric Neurosurgery. 1999;15(9):461-7. https://doi.org/10.1007/s003810050440.

Kitzler TM, Schneider R, Kohl S, Kolvenbach CM, Connaughton DM, Dai R, et al. COL4A1 mutations as a potential novel cause of autosomal dominant CAKUT in humans. Human genetics. 2019;138(10):1105-15. https://doi.org/10.1007/s00439-019-02042-4.

Klatman EL, McKee M, Ogle GD. Documenting and visualising progress towards Universal Health Coverage of insulin and blood glucose test strips for people with diabetes. Diabetes research and clinical practice. 2019;157:107859. https://doi.org/10.1016/j.diabres.2019.107859.

Ko JS, Yi NJ, Suh KS, Seo JK. Pediatric liver transplantation for fibropolycystic liver disease. Pediatr Transplant. 2012;16(2):195-200. https://doi.org/10.1111/j.1399-3046.2012.01661.x.

Koleva M, De Jesus O. Hydrocephalus. StatPearls. Treasure Island (FL): StatPearls Publishing Copyright © 2022, StatPearls Publishing LLC.; 2022.

Komlakh K, Oveisi H, Hossein Aghamiri S. Endoscopic third ventricolustomy as treatment option for normal pressure hydrocephalus. European journal of translational myology. 2022;32(4). https://doi.org/10.4081/ejtm.2022.10618.

Korfias SI, Banos S, Alexoudi A, Themistoklis K, Vlachakis E, Patrikelis P, et al. Telemetric intracranial pressure monitoring: our experience with 22 patients investigated for intracranial hypertension. British journal of neurosurgery. 2021;35(4):430-7. https://doi.org/10.1080/02688697.2020.1849544.

Kosteniuk JG, Dickinson HD. Tracing the social gradient in the health of Canadians: primary and secondary determinants. Social science & medicine (1982). 2003;57(2):263-76. https://doi.org/10.1016/s0277-9536(02)00345-3.

Krauss JK, Regel JP, Droste DW, Orszagh M, Borremans JJ, Vach W. Movement disorders in adult hydrocephalus. Movement disorders : official journal of the Movement Disorder Society. 1997;12(1):53-60. https://doi.org/10.1002/mds.870120110.

Kristensen PL, Tarnow L, Bay C, Nørgaard K, Jensen T, Parving HH, et al. Comparing effects of insulin analogues and human insulin on nocturnal glycaemia in hypoglycaemia-prone people with Type 1 diabetes. Diabetic medicine : a journal of the British Diabetic Association. 2017;34(5):625-31. https://doi.org/10.1111/dme.13317.

Kroiss S, Huck K, Berthold S, Rüschendorf F, Scolari F, Caridi G, et al. Evidence of further genetic heterogeneity in autosomal dominant medullary cystic kidney disease. Nephrol Dial Transplant. 2000;15(6):818-21. https://doi.org/10.1093/ndt/15.6.818.

Kumada S, Hayashi M, Arima K, Nakayama H, Sugai K, Sasaki M, et al. Renal disease in Arima syndrome is nephronophthisis as in other Joubert-related Cerebello-oculo-renal syndromes. American journal of medical genetics Part A. 2004;131(1):71-6. https://doi.org/10.1002/ajmg.a.30294.

Kurnaz E, Aycan Z, Yıldırım N, Çetinkaya S. Conventional insulin pump therapy in two neonatal diabetes patients harboring the homozygous PTF1A enhancer mutation: Need for a novel approach for the management of neonatal diabetes. The Turkish journal of pediatrics. 2017;59(4):458-62. https://doi.org/10.24953/turkjped.2017.04.013.

Kurtoglu S, Atabek ME, Dizdarer C, Pirgon O, Isguven P, Emek S. Insulin detemir improves glycemic control and reduces hypoglycemia in children with type 1 diabetes: findings from the Turkish cohort of the PREDICTIVE observational study. Pediatric diabetes. 2009;10(6):401-7. https://doi.org/10.1111/j.1399-5448.2008.00497.x.

Lalou DA, Czosnyka M, Donnelly J, Lavinio A, Pickard JD, Garnett M, et al. Are Slow Waves of Intracranial Pressure Suppressed by General Anaesthesia? Acta neurochirurgica Supplement. 2018;126:129-32. https://doi.org/10.1007/978-3-319-65798-1_27.

Landing BH, Wells TR, Lipsey AI, Oyemade OA. Morphometric studies of cystic and tubulointerstitial kidney diseases with hepatic fibrosis in children. Pediatric pathology. 1990;10(6):959-72. https://doi.org/10.3109/15513819009064730.

Laranjeira FO, Silva EN, Pereira MG. Budget Impact of Long-Acting Insulin Analogues: The Case in Brazil. PLoS One. 2016;11(12):e0167039. https://doi.org/10.1371/journal.pone.0167039.

Larose S, Filliter C, Platt RW, Yu OHY, Filion KB. Long-acting insulin analogues and the risk of diabetic retinopathy among patients with type 2 diabetes: A population-based cohort study. Diabetes, obesity & metabolism. 2023;25(8):2279-89. https://doi.org/10.1111/dom.15106.

Lasebikan VO, Ayinde O, Odunleye M. Assessment of the alcohol consumption among outdoor bar drinkers in Nigeria by qualitative methods. BMC public health. 2018;18(1):318. https://doi.org/10.1186/s12889-018-5250-y.

Leary OP, Svokos KA, Klinge PM. Reappraisal of Pediatric Normal-Pressure Hydrocephalus. J Clin Med. 2021;10(9). https://doi.org/10.3390/jcm10092026.

Lee MJ, Chang CP, Lee YH, Wu YC, Tseng HW, Tung YY, et al. Longitudinal evaluation of an N-ethyl-N-nitrosourea-created murine model with normal pressure hydrocephalus. PLoS One. 2009;4(11):e7868. https://doi.org/10.1371/journal.pone.0007868.

Li J, Wang Y, Han L, Sun X, Yu H, Yu Y. Time-action profile of an oral enteric insulin formulation in healthy Chinese volunteers. Clinical therapeutics. 2012;34(12):2333-8. https://doi.org/10.1016/j.clinthera.2012.11.004.

Li W, Petersen RC, Algeciras-Schimnich A, Cogswell PM, Bornhorst JA, Kremers WK, et al. Alzheimer Disease Cerebrospinal Fluid Biomarkers in a Tertiary Neurology Practice. Mayo Clin Proc. 2024;99(8):1284-96. https://doi.org/10.1016/j.mayocp.2023.12.024.

Liang N, Jiang X, Zeng L, Li Z, Liang D, Wu L. 28 novel mutations identified from 33 Chinese patients with cilia-related kidney disorders. Clinica chimica acta; international journal of clinical chemistry. 2020;501:207-15. https://doi.org/10.1016/j.cca.2019.10.040.

Lilja A, Andresen M, Hadi A, Christoffersen D, Juhler M. Clinical experience with telemetric intracranial pressure monitoring in a Danish neurosurgical center. Clinical neurology and neurosurgery. 2014;120:36-40. https://doi.org/10.1016/j.clineuro.2014.02.010.

Limsuwan A, Platoshyn O, Yu Y, Rubin LJ, Rothman A, Yuan JX. Inhibition of K(+) channel activity in human pulmonary artery smooth muscle cells by serum from patients with pulmonary hypertension secondary to congenital heart disease. Pediatric research. 2001;50(1):23-8. https://doi.org/10.1203/00006450-200107000-00007.

Lin JC, Shau WY, Lai MS. Long-acting insulin analogues and diabetic retinopathy: a retrospective cohort study. Clinical therapeutics. 2014;36(9):1255-68. https://doi.org/10.1016/j.clinthera.2014.06.031.

Lindstrand A, Davis EE, Carvalho CM, Pehlivan D, Willer JR, Tsai IC, et al. Recurrent CNVs and SNVs at the NPHP1 locus contribute pathogenic alleles to Bardet-Biedl syndrome. Am J Hum Genet. 2014;94(5):745-54. https://doi.org/10.1016/j.ajhg.2014.03.017.

Liu JT, Su PH. The efficacy and limitation of lumboperitoneal shunt in normal pressure hydrocephalus. Clinical neurology and neurosurgery. 2020;193:105748. https://doi.org/10.1016/j.clineuro.2020.105748.

Liu M, Zhou Z, Yan J, Li P, Song W, Fu J, et al. A randomised, open-labelstudy of insulin glargine or neutral protamine Hagedorn insulin in Chinese paediatric patients with type 1 diabetes mellitus. BMC endocrine disorders. 2016;16(1):67. https://doi.org/10.1186/s12902-016-0146-2.

Lollis SS, Mamourian AC, Vaccaro TJ, Duhaime AC. Programmable CSF shunt valves: radiographic identification and interpretation. AJNR American journal of neuroradiology. 2010;31(7):1343-6. https://doi.org/10.3174/ajnr.A1997.

Lteif AN, Schwenk WF. Accuracy of pen injectors versus insulin syringes in children with type 1 diabetes. Diabetes care. 1999;22(1):137-40. https://doi.org/10.2337/diacare.22.1.137.

Lucidi P, Porcellati F, Marinelli Andreoli A, Carriero I, Candeloro P, Cioli P, et al. Pharmacokinetics and Pharmacodynamics of NPH Insulin in Type 1 Diabetes: The Importance of Appropriate Resuspension Before Subcutaneous Injection. Diabetes care. 2015;38(12):2204-10. https://doi.org/10.2337/dc15-0801.

Ludvigsson J, Bolli GB. Intensive insulin treatment in diabetic children. Diabetes, nutrition & metabolism. 2001;14(5):292-304. https://www.researchgate.net/publication/11553630_Intensive_insulin_treatment_in_diabetic_children.

Luo Y, Xia J, Zhao Z, Chang Y, Bee YM, Nguyen KT, et al. Effectiveness, safety, initial optimal dose, and optimal maintenance dose range of basal insulin regimens for type 2 diabetes: A systematic review with meta-analysis. Journal of diabetes. 2023;15(5):419-35. https://doi.org/10.1111/1753-0407.13381.

Ly KR, Saito S, Kusama K. Cooperation between Japanese and Cambodian Dietitians in Setting up a Hospital Diet Management System. Journal of nutritional science and vitaminology. 2015;61 Suppl:S58-9. https://doi.org/10.3177/jnsv.61.S58.

Mai JK, Lensing-Höhn S, Ende AA, Sofroniew MV. Developmental organization of neurophysin neurons in the human brain. The Journal of comparative neurology. 1997;385(3):477-89. https://doi.org/10.1002/(SICI)1096-9861(19970901)385:3<477::AID-CNE10>3.0.CO;2-H.

Mao M, Zhang L, Ge J, Yan J, Northington R, Yao M, et al. Infant Feeding Regimens and Gastrointestinal Tolerance: A Multicenter, Prospective, Observational Cohort Study in China. Global pediatric health. 2018;5:2333794x17750271. https://doi.org/10.1177/2333794x17750271.

Maria M, Lamers IJ, Schmidts M, Ajmal M, Jaffar S, Ullah E, et al. Genetic and clinical characterization of Pakistani families with Bardet-Biedl syndrome extends the genetic and phenotypic spectrum. Sci Rep. 2016;6:34764. https://doi.org/10.1038/srep34764.

Maron JL, Hwang JS, Pathak S, Ruthazer R, Russell RL, Alterovitz G. Computational gene expression modeling identifies salivary biomarker analysis that predict oral feeding readiness in the newborn. J Pediatr. 2015;166(2):282-8.e5. https://doi.org/10.1016/j.jpeds.2014.10.065.

Marra LP, Araújo VE, Oliveira GC, Diniz LM, Guerra Júnior AA, Acurcio FA, et al. The clinical effectiveness of insulin glargine in patients with Type I diabetes in Brazil: findings and implications. Journal of comparative effectiveness research. 2017;6(6):519-27. https://doi.org/10.2217/cer-2016-0099.

Marzouk A, Lajili M, Ben Yahya I, Thebti R, Ayeb S, Bouaziz A. Transitioning to Insulin Analogs in Tunisian Children with Type 1 Diabetes: Efficacy and Safety. Tunis Med. 2024;102(8):452-6. https://doi.org/10.62438/tunismed.v102i8.4435.

Mascalchi M, Arnetoli G, Inzitari D, Dal Pozzo G, Lolli F, Caramella D, et al. Cine-MR imaging of aqueductal CSF flow in normal pressure hydrocephalus syndrome before and after CSF shunt. Acta radiologica (Stockholm, Sweden : 1987). 1993;34(6):586-92. https://doi.org/10.3109/02841859309175413.

Masuda H, Sakamoto M, Irie J, Kitaoka A, Shiono K, Inoue G, et al. Comparison of twice-daily injections of biphasic insulin lispro and basal-bolus therapy: glycaemic control and quality-of-life of insulin-naïve type 2 diabetic patients. Diabetes, obesity & metabolism. 2008;10(12):1261-5. https://doi.org/10.1111/j.1463-1326.2008.00897.x.

Mayordomo-Colunga J, Rey C, Medina A, Martínez-Camblor P, Vivanco-Allende A, Concha A. Helmet Versus Nasal-Prong CPAP in Infants With Acute Bronchiolitis. Respiratory care. 2018;63(4):455-63. https://doi.org/10.4187/respcare.05840.

McAllister JP, 2nd, Williams MA, Walker ML, Kestle JR, Relkin NR, Anderson AM, et al. An update on research priorities in hydrocephalus: overview of the third National Institutes of Health-sponsored symposium "Opportunities for Hydrocephalus Research: Pathways to Better Outcomes". Journal of neurosurgery. 2015;123(6):1427-38. https://doi.org/10.3171/2014.12.Jns132352.

McGovern RA, Kelly KM, Chan AK, Morrissey NJ, McKhann GM, 2nd. Should ventriculoatrial shunting be the procedure of choice for normal-pressure hydrocephalus? Journal of neurosurgery. 2014;120(6):1458-64. https://doi.org/10.3171/2014.1.Jns131808.

McKeage K, Goa KL. Insulin glargine: a review of its therapeutic use as a long-acting agent for the management of type 1 and 2 diabetes mellitus. Drugs. 2001;61(11):1599-624. https://doi.org/10.2165/00003495-200161110-00007.

McKeage K, Goa KL. Spotlight on insulin glargine in type 1 and 2 diabetes mellitus. Treatments in endocrinology. 2002;1(1):55-8. https://doi.org/10.2165/00024677-200201010-00006.

Meier U, Kintzel D. Clinical experiences with different valve systems in patients with normal-pressure hydrocephalus: evaluation of the Miethke dual-switch valve. Child's nervous system : ChNS : official

journal of the International Society for Pediatric Neurosurgery. 2002;18(6-7):288-94. https://doi.org/10.1007/s00381-002-0577-0.

Mendley SR, Poznanski AK, Spargo BH, Langman CB. Hereditary sclerosing glomerulopathy in the conorenal syndrome. Am J Kidney Dis. 1995;25(5):792-7. https://doi.org/10.1016/0272-6386(95)90556-1.

Meng T, He H, Liu H, Lv X, Huang C, Zhong L, et al. Investigation of the feasibility of synthetic MRI in the differential diagnosis of non-keratinising nasopharyngeal carcinoma and benign hyperplasia using different contoured methods for delineation of the region of interest. Clinical radiology. 2021;76(3):238.e9-.e15. https://doi.org/10.1016/j.crad.2020.10.010.

Mesquita RF, Reis M, Beppler AP, Bellinazzi VR, Mattos SS, Lima-Filho JL, et al. Onset of hypertension during pregnancy is associated with long-term worse blood pressure control and adverse cardiac remodeling. Journal of the American Society of Hypertension : JASH. 2014;8(11):827-31. https://doi.org/10.1016/j.jash.2014.09.006.

Mianowska B, Szadkowska A, Pietrzak I, Zmysłowska A, Wegner O, Tomczonek J, et al. Immunogenicity of different brands of human insulin and rapid-acting insulin analogs in insulin-naïve children with type 1 diabetes. Pediatric diabetes. 2011;12(2):78-84. https://doi.org/10.1111/j.1399-5448.2010.00659.x.

Miles HL, Acerini CL. Insulin analog preparations and their use in children and adolescents with type 1 diabetes mellitus. Paediatric drugs. 2008;10(3):163-76. https://doi.org/10.2165/00148581-200810030-00005.

Miltenburg AM, Prohn M, van Kuijk JH, Tiessen RG, de Kort M, Berg RJ. Half-life prolongation of therapeutic proteins by conjugation to ATIII-binding pentasaccharides: a first-in-human study of CarboCarrier® insulin. British journal of clinical pharmacology. 2013;75(5):1221-30. https://doi.org/10.1111/j.1365-2125.2012.04460.x.

Mitamura R, Kimura H, Murakami Y, Nagaya K, Makita Y, Okuno A. Ultralente insulin treatment of transient neonatal diabetes mellitus. J Pediatr. 1996;128(2):268-70. https://doi.org/10.1016/s0022-3476(96)70406-7.

Mohn A, Matyka KA, Harris DA, Ross KM, Edge JA, Dunger DB. Lispro or regular insulin for multiple injection therapy in adolescence. Differences in free insulin and glucose levels overnight. Diabetes care. 1999;22(1):27-32. https://doi.org/10.2337/diacare.22.1.27.

Monami M, Marchionni N, Mannucci E. Long-acting insulin analogues vs. NPH human insulin in type 1 diabetes. A meta-analysis. Diabetes, obesity & metabolism. 2009;11(4):372-8. https://doi.org/10.1111/j.1463-1326.2008.00976.x.

Moraes D, Munhoz TP, Pinheiro da Costa BE, Hentschke MR, Sontag F, Silveira Lucas L, et al. Immature platelet fraction in hypertensive pregnancy. Platelets. 2016;27(4):333-7. https://doi.org/10.3109/09537104.2015.1101060.

Morimoto Y, Yoshida S, Kinoshita A, Satoh C, Mishima H, Yamaguchi N, et al. Nonsense mutation in CFAP43 causes normal-pressure hydrocephalus with ciliary abnormalities. Neurology. 2019;92(20):e2364-e74. https://doi.org/10.1212/wnl.0000000000007505.

Mortensen H, Kocova M, Teng LY, Keiding J, Bruckner I, Philotheou A. Biphasic insulin aspart vs. human insulin in adolescents with type 1 diabetes on multiple daily insulin injections. Pediatric diabetes. 2006;7(1):4-10. https://doi.org/10.1111/j.1399-543X.2006.00138.x.

Mudaliar SR, Mohideen P, Baxi SC, Joyce M, Armstrong DA, Strange P, et al. Pharmacodynamic and pharmacokinetic properties of a premixed 85/15 human insulin preparation. Clinical therapeutics. 2001;23(3):404-12. https://doi.org/10.1016/s0149-2918(01)80045-x.

Müller N, Kloos C, Sämann A, Wolf G, Müller UA. Evaluation of a treatment and teaching refresher programme for the optimization of intensified insulin therapy in type 1 diabetes. Patient Educ Couns. 2013;93(1):108-13. https://doi.org/10.1016/j.pec.2013.05.008.

Murphy NP, Keane SM, Ong KK, Ford-Adams M, Edge JA, Acerini CL, et al. Randomized cross-over trial of insulin glargine plus lispro or NPH insulin plus regular human insulin in adolescents with type 1 diabetes on intensive insulin regimens. Diabetes care. 2003;26(3):799-804. https://doi.org/10.2337/diacare.26.3.799.

Mysiw WJ, Jackson RD. Relationship of new-onset systemic hypertension and normal pressure hydrocephalus. Brain injury. 1990;4(3):233-8. https://doi.org/10.3109/02699059009026172.

Nabaweesi R, Ramakrishnaiah RH, Aitken ME, Rettiganti MR, Luo C, Maxson RT, et al. Injured Children Receive Twice the Radiation Dose at Nonpediatric Trauma Centers Compared With Pediatric Trauma Centers. Journal of the American College of Radiology : JACR. 2018;15(1 Pt A):58-64. https://doi.org/10.1016/j.jacr.2017.06.035.

Naess H, Nyland H. Poor health-related quality of life is associated with long-term mortality in young adults with cerebral infarction. Journal of stroke and cerebrovascular diseases : the official journal of National Stroke Association. 2013;22(7):e79-83. https://doi.org/10.1016/j.jstrokecerebrovasdis.2012.06.010.

Naftel RP, Argo JL, Shannon CN, Taylor TH, Tubbs RS, Clements RH, et al. Laparoscopic versus open insertion of the peritoneal catheter in ventriculoperitoneal shunt placement: review of 810 consecutive cases. Journal of neurosurgery. 2011;115(1):151-8. https://doi.org/10.3171/2011.1.Jns101492.

Nakajima M, Yamada S, Miyajima M, Ishii K, Kuriyama N, Kazui H, et al. Guidelines for Management of Idiopathic Normal Pressure Hydrocephalus (Third Edition): Endorsed by the Japanese Society of Normal Pressure Hydrocephalus. Neurologia medico-chirurgica. 2021;61(2):63-97. https://doi.org/10.2176/nmc.st.2020-0292.

Negrato CA, Rafacho A, Negrato G, Teixeira MF, Araújo CA, Vieira L, et al. Glargine vs. NPH insulin therapy in pregnancies complicated by diabetes: an observational cohort study. Diabetes research and clinical practice. 2010;89(1):46-51. https://doi.org/10.1016/j.diabres.2010.03.015.

Neklyudova A, Kuramagomedova R, Voinova V, Sysoeva O. Atypical brain responses to 40-Hz click trains in girls with Rett syndrome: Auditory steady-state response and sustained wave. Psychiatry and clinical neurosciences. 2024;78(5):282-90. https://doi.org/10.1111/pcn.13638.

Nell LJ, Thomas JW. Frequency and specificity of protamine antibodies in diabetic and control subjects. Diabetes. 1988;37(2):172-6. https://doi.org/10.2337/diab.37.2.172.

Newfield RS, Cohen D, Capparelli EV, Shragg P. Rapid weight gain in children soon after diagnosis of type 1 diabetes: is there room for concern? Pediatric diabetes. 2009;10(5):310-5. https://doi.org/10.1111/j.1399-5448.2008.00475.x.

Newman KD, Weaver MT. Insulin measurement and preparation among diabetic patients at a county hospital. The Nurse practitioner. 1994;19(3):44-5, 8. https://doi.org/10.1097/00006205-199403000-00011.

Nexøe-Larsen CC, Sørensen PH, Hausner H, Agersnap M, Baekdal M, Brønden A, et al. Effects of liraglutide on gallbladder emptying: A randomized, placebo-controlled trial in adults with overweight or obesity. Diabetes, obesity & metabolism. 2018;20(11):2557-64. https://doi.org/10.1111/dom.13420.

Nigim F, Critchlow JF, Schneider BE, Chen C, Kasper EM. Shunting for hydrocephalus: analysis of techniques and failure patterns. The Journal of surgical research. 2014;191(1):140-7. https://doi.org/10.1016/j.jss.2014.03.075.

Nunn AC, Jones HE, Morosanu CO, Singleton WGB, Williams MA, Nagel SJ, et al. Extended lumbar drainage in idiopathic normal pressure hydrocephalus: a systematic review and meta-analysis of diagnostic test accuracy. British journal of neurosurgery. 2021;35(3):285-91. https://doi.org/10.1080/02688697.2020.1787948.

Nuovo S, Fuiano L, Micalizzi A, Battini R, Bertini E, Borgatti R, et al. Impaired urinary concentration ability is a sensitive predictor of renal disease progression in Joubert syndrome. Nephrol Dial Transplant. 2020;35(7):1195-202. https://doi.org/10.1093/ndt/gfy333.

Omran H, Sasmaz G, Häffner K, Volz A, Olbrich H, Melkaoui R, et al. Identification of a gene locus for Senior-Løken syndrome in the region of the nephronophthisis type 3 gene. Journal of the American Society of Nephrology : JASN. 2002;13(1):75-9. https://doi.org/10.1681/asn.V13175.

Ost M, Nylén K, Csajbok L, Ohrfelt AO, Tullberg M, Wikkelsö C, et al. Initial CSF total tau correlates with 1-year outcome in patients with traumatic brain injury. Neurology. 2006;67(9):1600-4. https://doi.org/10.1212/01.wnl.0000242732.06714.0f.

Otaki Y, Watanabe T, Sato J, Kobayashi Y, Aono T, Saito Y, et al. Association of Nephronophthisis 4 genetic variation with cardiorenal syndrome and cardiovascular events in Japanese general population: the Yamagata (Takahata) study. Heart and vessels. 2022;37(4):673-82. https://doi.org/10.1007/s00380-021-01953-5.

Otto EA, Trapp ML, Schultheiss UT, Helou J, Quarmby LM, Hildebrandt F. NEK8 mutations affect ciliary and centrosomal localization and may cause nephronophthisis. Journal of the American Society of Nephrology : JASN. 2008;19(3):587-92. https://doi.org/10.1681/asn.2007040490.

Owens DR, Jones AL, Dolben J, Dean JD, Petocz P, Coates PA, et al. The pharmacokinetics of five pre-mixed combinations of 'short-' and 'intermediate-acting' (NPH) insulins in healthy subjects following subcutaneous administration. Diabetes research (Edinburgh, Scotland). 1993;22(2):77-86.

Päivärinta M, Tapanainen P, Veijola R. Basal insulin switch from NPH to glargine in children and adolescents with type 1 diabetes. Pediatric diabetes. 2008;9(3 Pt 2):83-90. https://doi.org/10.1111/j.1399-5448.2007.00341.x.

Palmer AJ, Roze S, Valentine WJ, Smith I, Wittrup-Jensen KU. Cost-effectiveness of detemir-based basal/bolus therapy versus NPH-based basal/bolus therapy for type 1 diabetes in a UK setting: an economic analysis based on meta-analysis results of four clinical trials. Current medical research and opinion. 2004;20(11):1729-46. https://doi.org/10.1185/030079904x5661.

Pandya N, DiGenio A, Gao L, Patel M. Efficacy and safety of insulin glargine compared to other interventions in younger and older adults: a pooled analysis of nine open-label, randomized controlled trials in patients with type 2 diabetes. Drugs & aging. 2013;30(6):429-38. https://doi.org/10.1007/s40266-013-0069-9.

Pańkowska E, Nazim J, Szalecki M, Urban M. Equal metabolic control but superior caregiver treatment satisfaction with insulin aspart in preschool children. Diabetes technology & therapeutics. 2010;12(5):413-8. https://doi.org/10.1089/dia.2009.0155.

Parasyri M, Brandström P, Uusimaa J, Ostergaard E, Hikmat O, Isohanni P, et al. Renal Phenotype in Mitochondrial Diseases: A Multicenter Study. Kidney diseases (Basel, Switzerland). 2022;8(2):148-59. https://doi.org/10.1159/000521148.

Parisi M, Glass I. Joubert Syndrome. In: Adam MP, Everman DB, Mirzaa GM, Pagon RA, Wallace SE, Bean LJH, et al., editors. GeneReviews(®). Seattle (WA): University of Washington, Seattle Copyright © 1993-2023, University of Washington, Seattle. GeneReviews is a registered trademark of the University of Washington, Seattle. All rights reserved.; 1993.

Parisi MA, Doherty D, Eckert ML, Shaw DW, Ozyurek H, Aysun S, et al. AHI1 mutations cause both retinal dystrophy and renal cystic disease in Joubert syndrome. J Med Genet. 2006;43(4):334-9. https://doi.org/10.1136/jmg.2005.036608.

Park E, Lee JM, Ahn YH, Kang HG, Ha, II, Lee JH, et al. Hepatorenal fibrocystic diseases in children. Pediatr Nephrol. 2016;31(1):113-9. https://doi.org/10.1007/s00467-015-3185-4.

Park EH, Eide PK, Zurakowski D, Madsen JR. Impaired pulsation absorber mechanism in idiopathic normal pressure hydrocephalus: laboratory investigation. Journal of neurosurgery. 2012;117(6):1189-96. https://doi.org/10.3171/2012.9.Jns121227.

Perrault I, Halbritter J, Porath JD, Gérard X, Braun DA, Gee HY, et al. IFT81, encoding an IFT-B core protein, as a very rare cause of a ciliopathy phenotype. J Med Genet. 2015;52(10):657-65. https://doi.org/10.1136/jmedgenet-2014-102838.

Petit-Bibal C, Rothenbuhler A, Lucchini P, Aboumrad B, Castell AL, Le Fur S, et al. Decrease in clinical hypoglycemia in young children with type 1 diabetes treated with free-mixed aspart and detemir insulin: an open labeled randomized trial. Pediatric diabetes. 2015;16(5):345-53. https://doi.org/10.1111/pedi.12156.

Pfohl M, Schädlich PK, Dippel FW, Koltermann KC. Health economic evaluation of insulin glargine vs NPH insulin in intensified conventional therapy for type 1 diabetes in Germany. Journal of medical economics. 2012;15 Suppl 2:14-27. https://doi.org/10.3111/13696998.2012.713879.

Pieber TR, Draeger E, Kristensen A, Grill V. Comparison of three multiple injection regimens for Type 1 diabetes: morning plus dinner or bedtime administration of insulin detemir vs. morning plus bedtime NPH insulin. Diabetic medicine : a journal of the British Diabetic Association. 2005;22(7):850-7. https://doi.org/10.1111/j.1464-5491.2005.01545.x.

Pitocco D, Crinò A, Di Stasio E, Manfrini S, Guglielmi C, Spera S, et al. The effects of calcitriol and nicotinamide on residual pancreatic beta-cell function in patients with recent-onset Type 1 diabetes (IMDIAB XI). Diabetic medicine : a journal of the British Diabetic Association. 2006;23(8):920-3. https://doi.org/10.1111/j.1464-5491.2006.01921.x.

Plager P, Nurie K, Omann T, Moran A, Piloya T, Bahendeka S, et al. Is insulin diluted when stored in water? Pediatric diabetes. 2017;18(3):237-40. https://doi.org/10.1111/pedi.12367.

Plank J, Bodenlenz M, Sinner F, Magnes C, Görzer E, Regittnig W, et al. A double-blind, randomized, dose-response study investigating the pharmacodynamic and pharmacokinetic properties of the long-acting insulin analog detemir. Diabetes care. 2005;28(5):1107-12. https://doi.org/10.2337/diacare.28.5.1107.

Pollex E, Moretti ME, Koren G, Feig DS. Safety of insulin glargine use in pregnancy: a systematic review and meta-analysis. The Annals of pharmacotherapy. 2011;45(1):9-16. https://doi.org/10.1345/aph.1P327.

Popović-Rolović M, Kostić M, Sindjić M, Jovanović O, Peco-Antić A, Kruscić D. Progressive tubulointerstitial nephritis and chronic cholestatic liver disease. Pediatr Nephrol. 1993;7(4):396-400. https://doi.org/10.1007/bf00857550.

Porcellati F, Lin J, Lucidi P, Bolli GB, Fanelli CG. Impact of patient and treatment characteristics on glycemic control and hypoglycemia in patients with type 2 diabetes initiated to insulin glargine or NPH: A post hoc, pooled, patient-level analysis of 6 randomized controlled trials. Medicine. 2017;96(5):e6022. https://doi.org/10.1097/md.0000000000006022.

Pöyhönen-Alho M, Rönnemaa T, Saltevo J, Ekblad U, Kaaja RJ. Use of insulin glargine during pregnancy. Acta obstetricia et gynecologica Scandinavica. 2007;86(10):1171-4. https://doi.org/10.1080/00016340701515282.

Pozzilli P, Manfrini S, Buzzetti R, Lampeter E, Leeuw ID, Iafusco D, et al. Glucose evaluation trial for remission (GETREM) in type 1 diabetes: a European multicentre study. Diabetes research and clinical practice. 2005;68(3):258-64. https://doi.org/10.1016/j.diabres.2004.10.001.

Quaglia M, Musetti C, Ghiggeri GM, Fogazzi GB, Settanni F, Boldorini RL, et al. Unexpectedly high prevalence of rare genetic disorders in kidney transplant recipients with an unknown causal nephropathy. Clinical transplantation. 2014;28(9):995-1003. https://doi.org/10.1111/ctr.12408.

Quattrin T, Bélanger A, Bohannon NJ, Schwartz SL. Efficacy and safety of inhaled insulin (Exubera) compared with subcutaneous insulin therapy in patients with type 1 diabetes: results of a 6-month, randomized, comparative trial. Diabetes care. 2004;27(11):2622-7. https://doi.org/10.2337/diacare.27.11.2622.

Quattrin T, Thrailkill K, Baker L, Kuntze J, Compton P, Martha P. Improvement of HbA1c without increased hypoglycemia in adolescents and young adults with type 1 diabetes mellitus treated with recombinant human insulin-like growth factor-I and insulin. rhIGF-I in IDDM Study Group. Journal of pediatric endocrinology & metabolism : JPEM. 2001;14(3):267-77. https://doi.org/10.1515/jpem.2001.14.3.267.

Raskin P, Guthrie RA, Leiter L, Riis A, Jovanovic L. Use of insulin aspart, a fast-acting insulin analog, as the mealtime insulin in the management of patients with type 1 diabetes. Diabetes care. 2000;23(5):583-8. https://doi.org/10.2337/diacare.23.5.583.

Rauchman M, Hoffman WH, Hanna JD, Kulharya AS, Figueroa RE, Yang J, et al. Exclusion of SIX6 hemizygosity in a child with anophthalmia, panhypopituitarism and renal failure. American journal of medical genetics. 2001;104(1):31-6. https://doi.org/10.1002/ajmg.10016.

Razavi Z, Ahmadi M. Efficacy of Thrice-daily versus Twice-daily Insulin Regimens on Glycohemoglobin (Hb A1c) in Type 1 Diabetes Mellitus: A Randomized Controlled Trial. Oman medical journal. 2011;26(1):10-3. https://doi.org/10.5001/omj.2011.03.

Reynolds NA, Wagstaff AJ. Insulin aspart: a review of its use in the management of type 1 or 2 diabetes mellitus. Drugs. 2004;64(17):1957-74. https://doi.org/10.2165/00003495-200464170-00013.

Rigo J, Hascoët JM, Picaud JC, Mosca F, Rubio A, Saliba E, et al. Comparative study of preterm infants fed new and existing human milk fortifiers showed favourable markers of gastrointestinal status. Acta paediatrica (Oslo, Norway : 1992). 2020;109(3):527-33. https://doi.org/10.1111/apa.14981.

Rinaldo L, Brown D, Lanzino G, Parney IF. Outcomes following cerebrospinal fluid shunting in high-grade glioma patients. Journal of neurosurgery. 2018;129(4):984-96. https://doi.org/10.3171/2017.6.Jns17859.

Rizzi VH, Sene LD, Fernandez CD, Gontijo JA, Boer PA. Impact of long-term high-fat diet intake gestational protein-restricted offspring on kidney morphology and function. Journal of developmental origins of health and disease. 2017;8(1):89-100. https://doi.org/10.1017/s2040174416000398.

Roach P, Strack T, Arora V, Zhao Z. Improved glycaemic control with the use of self-prepared mixtures of insulin lispro and insulin lispro protamine suspension in patients with types 1 and 2 diabetes. International journal of clinical practice. 2001;55(3):177-82. https://doi.org/10.1111/j.1742-1241.2001.tb11010.x.

Robert JJ, Chevenne D, Debray M. The contribution of intermediate-acting insulin preparations to daytime insulin treatment. Diabetic medicine : a journal of the British Diabetic Association. 1989;6(6):531-6. https://doi.org/10.1111/j.1464-5491.1989.tb01222.x.

Robertson KJ, Schoenle E, Gucev Z, Mordhorst L, Gall MA, Ludvigsson J. Insulin detemir compared with NPH insulin in children and adolescents with Type 1 diabetes. Diabetic medicine : a journal of the British Diabetic Association. 2007;24(1):27-34. https://doi.org/10.1111/j.1464-5491.2007.02024.x.

Rodríguez-Villar C, Conget I, Casamitjana R, Ercilla G, Gomis R. Effects of insulin administration in a group of high-risk, non-diabetic, first-degree relatives of Type 1 diabetic patients: an open pilot trial. Diabetic medicine : a journal of the British Diabetic Association. 1999;16(2):160-3. https://doi.org/10.1046/j.1464-5491.1999.00044.x.

Rodríguez-Villar C, Conget I, González-Clemente JM, Vidal J, Navarro P, Casamitjana R, et al. Effects of insulin administration on beta-cell function in subjects at high risk for type I diabetes mellitus. Metabolism: clinical and experimental. 1996;45(7):873-5. https://doi.org/10.1016/s0026-0495(96)90162-6.

Roland PS, Belcher BP, Bettis R, Makabale RL, Conroy PJ, Wall GM, et al. A single topical agent is clinically equivalent to the combination of topical and oral antibiotic treatment for otitis externa. American journal of otolaryngology. 2008;29(4):255-61. https://doi.org/10.1016/j.amjoto.2007.09.002.

Roland PS, Pien FD, Schultz CC, Henry DC, Conroy PJ, Wall GM, et al. Efficacy and safety of topical ciprofloxacin/dexamethasone versus neomycin/polymyxin B/hydrocortisone for otitis externa. Current medical research and opinion. 2004;20(8):1175-83. https://doi.org/10.1185/030079902125004312.

Rome K, Campbell R, Flint A, Haslock I. Heel pad thickness--a contributing factor associated with plantar heel pain in young adults. Foot & ankle international. 2002;23(2):142-7. https://doi.org/10.1177/107110070202300211.

Rönnemaa T, Viikari J. Reducing snacks when switching from conventional soluble to lispro insulin treatment: effects on glycaemic control and hypoglycaemia. Diabetic medicine : a journal of the British Diabetic Association. 1998;15(7):601-7. https://doi.org/10.1002/(sici)1096-9136(199807)15:7<601::Aid-dia627>3.0.Co;2-m.

Roos K, Lind L, Holm SE. Beta-lactamase production and bacterial tolerance in recurrent acute otitis media. International journal of pediatric otorhinolaryngology. 1991;21(2):155-61. https://doi.org/10.1016/0165-5876(91)90147-4.

Rostami PM, Setoodeh AM, Rabbani AM, Nakhaei-Moghadam MM, Najmi-Varzaneh FM, Rezaei NMP. A Randomized Clinical Trial of Insulin Glargine and Aspart, Compared to NPH and Regular Insulin in Children with Type 1 Diabetes Mellitus. Iranian journal of pediatrics. 2014;24(2):173-8. https://pmc.ncbi.nlm.nih.gov/articles/PMC4268837/.

Rubin DJ, Rybin D, Doros G, McDonnell ME. Weight-based, insulin dose-related hypoglycemia in hospitalized patients with diabetes. Diabetes care. 2011;34(8):1723-8. https://doi.org/10.2337/dc10-2434.

Ruiz de Adana MS, Colomo N, Maldonado-Araque C, Fontalba MI, Linares F, García-Torres F, et al. Randomized clinical trial of the efficacy and safety of insulin glargine vs. NPH insulin as basal insulin for the treatment of glucocorticoid induced hyperglycemia using continuous glucose monitoring in hospitalized patients with type 2 diabetes and respiratory disease. Diabetes research and clinical practice. 2015;110(2):158-65. https://doi.org/10.1016/j.diabres.2015.09.015.

Ruiz-de-Adana MS, Dominguez-Lopez ME, Gonzalez-Molero I, Machado A, Martin V, Cardona I, et al. Comparison between a multiple daily insulin injection regimen (basal once-daily glargine plus mealtime lispro) and continuous subcutaneous insulin infusion (lispro) using continuous glucose monitoring in metabolically optimized type 1 diabetes patients: A randomized open-labelled parallel study. Medicina clinica. 2016;146(6):239-46. https://doi.org/10.1016/j.medcli.2015.09.020.

Sacot A, López-Ros V, Prats-Puig A, Escosa J, Barretina J, Calleja-González J. Multidisciplinary Neuromuscular and Endurance Interventions on Youth Basketball Players: A Systematic Review with Meta-Analysis and Meta-Regression. Int J Environ Res Public Health. 2022;19(15). https://doi.org/10.3390/ijerph19159642.

Saeed MB, Sherif S. Pediatric renal transplantation in syria: a single center experience. Saudi J Kidney Dis Transpl. 2005;16(3):342-7. https://www.researchgate.net/publication/6195419_Pediatric_renal_transplantation_in_Syria_A_single_center_experience.

Sagna Y, Bagbila W, Bognounou R, Ilboudo A, Sawadogo N, Kyelem CG, et al. Comparison of regular with NPH insulin vs. premix insulin in children and adolescents with type 1 diabetes in a resources-limited setting: a retrospective data analysis. Journal of pediatric endocrinology & metabolism : JPEM. 2023;36(5):447-50. https://doi.org/10.1515/jpem-2022-0637.

Saiyed M, Saboo B, Pancholi M. Comparison of Analogue insulin with other insulins in patients with type 1 diabetes in Ahmedabad, Western India: A Rretrospective study. Diabetes & metabolic syndrome. 2020;14(6):1923-5. https://doi.org/10.1016/j.dsx.2020.09.025.

Salemyr J, Bang P, Örtqvist E. Lower HbA1c after 1 year, in children with type 1 diabetes treated with insulin glargine vs. NPH insulin from diagnosis: a retrospective study. Pediatric diabetes. 2011;12(5):501-5. https://doi.org/10.1111/j.1399-5448.2010.00723.x.

Salma A. Normal pressure hydrocephalus as a failure of ICP homeostasis mechanism: the hidden role of Monro-Kellie doctrine in the genesis of NPH. Child's nervous system : ChNS : official journal of the International Society for Pediatric Neurosurgery. 2014;30(5):825-30. https://doi.org/10.1007/s00381-014-2385-8.

Savoldelli RD, Farhat SC, Manna TD. Alternative management of diabetic ketoacidosis in a Brazilian pediatric emergency department. Diabetology & metabolic syndrome. 2010;2:41. https://doi.org/10.1186/1758-5996-2-41.

Sawathiparnich P, Kiattisakthavee P, Santiprabhob J, Likitmaskul S. Preliminary data of insulin glargine use among Thai adolescents and young adults with type 1 diabetes mellitus treated at Siriraj Hospital. Journal of the Medical Association of Thailand = Chotmaihet thangphaet. 2005;88 Suppl 8:S48-52. https://www.thaiscience.info/Journals/Article/JMAT/10791562.pdf.

Schaeffer AJ, Chow JS, Ivanova A, Cui G, Greenfield SP, Zerin JM, et al. Variation in the level of detail in pediatric voiding cystourethrogram reports. Journal of pediatric urology. 2017;13(3):257-62. https://doi.org/10.1016/j.jpurol.2016.08.025.

Schatlo B, Hamed M, Grote A, Schramm J, Neuloh G. Gravity assisted vs. medium pressure valves for communicating hydrocephalus show similar valve-revision rates. Acta neurochirurgica. 2013;155(10):1987-91. https://doi.org/10.1007/s00701-013-1852-4.

Schatz D, Cuthbertson D, Atkinson M, Salzler MC, Winter W, Muir A, et al. Preservation of C-peptide secretion in subjects at high risk of developing type 1 diabetes mellitus--a new surrogate measure of non-progression? Pediatric diabetes. 2004;5(2):72-9. https://doi.org/10.1111/j.1399-543X.2004.00047.x.

Schenker P, Stieglitz LH, Sick B, Stienen MN, Regli L, Sarnthein J. Patients with a Normal Pressure Hydrocephalus Shunt Have Fewer Complications than Do Patients with Other Shunts. World neurosurgery. 2018;110:e249-e57. https://doi.org/10.1016/j.wneu.2017.10.151.

Scherdel P, Reynaud R, Pietrement C, Salaün JF, Bellaïche M, Arnould M, et al. Priority target conditions for algorithms for monitoring children's growth: Interdisciplinary consensus. PLoS One. 2017;12(4):e0176464. https://doi.org/10.1371/journal.pone.0176464.

Schiaffini R, Ciampalini P, Spera S, Cappa M, Crinó A. An observational study comparing continuous subcutaneous insulin infusion (CSII) and insulin glargine in children with type 1 diabetes. Diabetes/metabolism research and reviews. 2005;21(4):347-52. https://doi.org/10.1002/dmrr.520.

Schiel R, Müller UA. Intensive or conventional insulin therapy in type 2 diabetic patients? A population-based study on metabolic control and quality of life (The JEVIN-trial). Experimental and clinical endocrinology & diabetes : official journal, German Society of Endocrinology [and] German Diabetes Association. 1999;107(8):506-11. https://doi.org/10.1055/s-0029-1232559.

Schmitt M, Eymann R, Antes S, Kiefer M. Intraventricular cooling during CSF infusion studies. Acta neurochirurgica Supplement. 2012;114:231-4. https://doi.org/10.1007/978-3-7091-0956-4_45.

Schneider GM, Jacobs DW, Gevirtz RN, O'Connor DT. Cardiovascular haemodynamic response to repeated mental stress in normotensive subjects at genetic risk of hypertension: evidence of enhanced reactivity, blunted adaptation, and delayed recovery. Journal of human hypertension. 2003;17(12):829-40. https://doi.org/10.1038/sj.jhh.1001624.

Schober E, Schoenle E, Van Dyk J, Wernicke-Panten K. Comparative trial between insulin glargine and NPH insulin in children and adolescents with type 1 diabetes. Diabetes care. 2001;24(11):2005-6. https://doi.org/10.2337/diacare.24.11.2005.

Schober E, Schoenle E, Van Dyk J, Wernicke-Panten K. Comparative trial between insulin glargine and NPH insulin in children and adolescents with type 1 diabetes mellitus. Journal of pediatric endocrinology & metabolism : JPEM. 2002;15(4):369-76. https://doi.org/10.1515/jpem.2002.15.4.369.

Scholtz HE, Pretorius SG, Wessels DH, Becker RH. Pharmacokinetic and glucodynamic variability: assessment of insulin glargine, NPH insulin and insulin ultralente in healthy volunteers using a euglycaemic clamp technique. Diabetologia. 2005;48(10):1988-95. https://doi.org/10.1007/s00125-005-1916-y.

Schröder S, Yigit G, Li Y, Altmüller J, Büttel HM, Fiedler B, et al. The genetic spectrum of congenital ocular motor apraxia type Cogan: an observational study, continued. Orphanet J Rare Dis. 2023;18(1):101. https://doi.org/10.1186/s13023-023-02706-5.

Schueler M, Braun DA, Chandrasekar G, Gee HY, Klasson TD, Halbritter J, et al. DCDC2 mutations cause a renal-hepatic ciliopathy by disrupting Wnt signaling. Am J Hum Genet. 2015;96(1):81-92. https://doi.org/10.1016/j.ajhg.2014.12.002.

Schurman SJ, Scheinman SJ. Inherited cerebrorenal syndromes. Nature reviews Nephrology. 2009;5(9):529-38. https://doi.org/10.1038/nrneph.2009.124.

Schuster MW, Chauhan SP, McLaughlin BN, Perry KG, Jr., Morrison JC. Comparison of insulin regimens and administration modalities in pregnancy complicated by diabetes. Journal of the Mississippi State Medical Association. 1998;39(2):51-5.

Schwartz TH, Yoon SS, Cutruzzola FW, Goodman RR. Third ventriculostomy: post-operative ventricular size and outcome. Minimally invasive neurosurgery : MIN. 1996;39(4):122-9. https://doi.org/10.1055/s-2008-1052231.

Scolari F, Ghiggeri GM, Casari G, Amoroso A, Puzzer D, Caridi GL, et al. Autosomal dominant medullary cystic disease: a disorder with variable clinical pictures and exclusion of linkage with the NPH1 locus. Nephrol Dial Transplant. 1998;13(10):2536-46. https://doi.org/10.1093/ndt/13.10.2536.

Scolari F, Valzorio B, Vizzardi V, Carli O, Costantino E, Viola F, et al. Nephronophthisis-medullary cystic kidney disease complex: a report on 24 patients from 5 families with Italian ancestry. Contributions to nephrology. 1997;122:61-3. https://doi.org/10.1159/000059870.

Secretariat MA. Continuous Subcutaneous Insulin Infusion (CSII) Pumps for Type 1 and Type 2 Adult Diabetic Populations: An Evidence-Based Analysis. Ontario health technology assessment series. 2009;9(20):1-58. https://pmc.ncbi.nlm.nih.gov/articles/PMC3377523/.

Senol D, Ozbag D, Dedeoglu N, Cevirgen F, Toy S, Ogeturk M, et al. Comparison of anthropometric and conic beam computed tomography measurements of patients with and without difficult intubation risk according to modified mallampati score: New markers for difficult intubation. Nigerian journal of clinical practice. 2021;24(11):1609-15. https://doi.org/10.4103/njcp.njcp_694_20.

Seruyange E, Gahutu JB, Mambo Muvunyi C, Uwimana ZG, Gatera M, Twagirumugabe T, et al. Measles seroprevalence, outbreaks, and vaccine coverage in Rwanda. Infectious diseases (London, England). 2016;48(11-12):800-7. https://doi.org/10.1080/23744235.2016.1201720.

Shah SC, Malone JI, Simpson NE. A randomized trial of intensive insulin therapy in newly diagnosed insulin-dependent diabetes mellitus. N Engl J Med. 1989;320(9):550-4. https://doi.org/10.1056/nejm198903023200902.

Shakhnovich AR, Razumovsky AY, Gasparjan SS, Ozerova VI. Venous and cerebrospinal fluid outflow in patients with brain swelling and oedema. Acta neurochirurgica Supplementum. 1990;51:357-61. https://doi.org/10.1007/978-3-7091-9115-6_121.

Sharplin P, Gordon J, Peters JR, Tetlow AP, Longman AJ, McEwan P. Improved glycaemic control by switching from insulin NPH to insulin glargine: a retrospective observational study. Cardiovascular diabetology. 2009;8:3. https://doi.org/10.1186/1475-2840-8-3.

Siman R, Toraskar N, Dang A, McNeil E, McGarvey M, Plaum J, et al. A panel of neuron-enriched proteins as markers for traumatic brain injury in humans. Journal of neurotrauma. 2009;26(11):1867-77. https://doi.org/10.1089/neu.2009.0882.

Skillgate E, Bill AS, Côté P, Viklund P, Peterson A, Holm LW. The effect of massage therapy and/or exercise therapy on subacute or long-lasting neck pain--the Stockholm neck trial (STONE): study protocol for a randomized controlled trial. Trials. 2015;16:414. https://doi.org/10.1186/s13063-015-0926-4.

Skogsberg L, Fors H, Hanas R, Chaplin JE, Lindman E, Skogsberg J. Improved treatment satisfaction but no difference in metabolic control when using continuous subcutaneous insulin infusion vs. multiple daily injections in children at onset of type 1 diabetes mellitus. Pediatric diabetes. 2008;9(5):472-9. https://doi.org/10.1111/j.1399-5448.2008.00390.x.

Skyler JS, Weinstock RS, Raskin P, Yale JF, Barrett E, Gerich JE, et al. Use of inhaled insulin in a basal/bolus insulin regimen in type 1 diabetic subjects: a 6-month, randomized, comparative trial. Diabetes care. 2005;28(7):1630-5. https://doi.org/10.2337/diacare.28.7.1630.

Sleeman A, Odom J, Schellinger M. Comparison of Hypoglycemia and Safety Outcomes With Long-Acting Insulins Versus Insulin NPH in Pregestational and Gestational Diabetes. The Annals of pharmacotherapy. 2020;54(7):669-75. https://doi.org/10.1177/1060028019897897.

Smith JG, Manuck TA, White J, Merrill DC. Insulin glargine versus neutral protamine Hagedorn insulin for treatment of diabetes in pregnancy. American journal of perinatology. 2009;26(1):57-62. https://doi.org/10.1055/s-0028-1095181.

Soliman AT, Omar M, Assem HM, Nasr IS, Rizk MM, El Matary W, et al. Serum leptin concentrations in children with type 1 diabetes mellitus: relationship to body mass index, insulin dose, and glycemic control. Metabolism: clinical and experimental. 2002;51(3):292-6. https://doi.org/10.1053/meta.2002.30502.

Solomon MD, Vijan S, Forma FM, Conrad RM, Summers NT, Lakdawalla DN. The impact of insulin type on severe hypoglycaemia events requiring inpatient and emergency department care in patients with type 2 diabetes. Diabetes research and clinical practice. 2013;102(3):175-82. https://doi.org/10.1016/j.diabres.2013.09.013.

Sørensen LP, Brock B, Mengel A, Rungby J, Moller N, Nielsen S, et al. Similarity of pharmacodynamic effects of a single injection of insulin glargine, insulin detemir and NPH insulin on glucose metabolism assessed by 24-h euglycaemic clamp studies in healthy humans. Diabetic medicine : a journal of the British Diabetic Association. 2010;27(7):830-7. https://doi.org/10.1111/j.1464-5491.2010.03026.x.

Soyaltın E, Kasap-Demir B, Alparslan C, Arslansoyu-Çamlar S, Öncel EP, Kırbıyık Ö, et al. Can a hand radiograph indicate a special diagnosis in a child with chronic kidney disease? Answers. Pediatr Nephrol. 2018;33(5):801-3. https://doi.org/10.1007/s00467-017-3742-0.

Soyaltın E, Kasap-Demir B, Alparslan C, Arslansoyu-Çamlar S, Öncel EP, Kırbıyık Ö, et al. Can a hand radiograph indicate a special diagnosis in a child with chronic kidney disease? Questions. Pediatr Nephrol. 2018;33(5):799-800. https://doi.org/10.1007/s00467-017-3740-2.

Stenfors LE, Räisänen S. Age-dependent changes in bacterial adherence to epithelial cells of nasopharynx in vivo. Acta oto-laryngologica. 1990;110(3-4):292-9. https://doi.org/10.3109/00016489009122551.

Stenfors LE, Räisänen S. Occurrence of middle ear pathogens in the nasopharynx of young individuals. A quantitative study in four age groups. Acta oto-laryngologica. 1990;109(1-2):142-8. https://doi.org/10.3109/00016489009107426.

Stenfors LE, Räisänen S. Secretory IgA- and IgG-coated bacteria in the nasopharynx of children. An immunofluorescence study. Acta oto-laryngologica. 1991;111(6):1139-45. https://doi.org/10.3109/00016489109100769.

Stenfors LE, Räisänen S. Bacterial attachment in vivo to epithelial cells of the nasopharynx during otitis media with effusion. The Journal of laryngology and otology. 1992;106(2):111-5. https://doi.org/10.1017/s0022215100118833.

Stenfors LE, Räisänen S. In vivo attachment of Streptococcus pneumoniae and Haemophilus influenzae to nasopharyngeal epithelium in children. ORL; journal for oto-rhino-laryngology and its related specialties. 1992;54(1):25-8. https://doi.org/10.1159/000276254.

Stenfors LE, Räisänen S. Secretory IgA-, IgG- and C3b-coated bacteria in the nasopharynx of otitis-prone and non-otitis-prone children. Acta oto-laryngologica. 1993;113(2):191-5. https://doi.org/10.3109/00016489309135791.

Stickelmeyer MP, Graf CJ, Frank BH, Ballard RL, Storms SM. Stability of U-10 and U-50 dilutions of insulin lispro. Diabetes technology & therapeutics. 2000;2(1):61-6. https://doi.org/10.1089/152091599316757.

Stippel M, Riedhammer KM, Lange-Sperandio B, Geßner M, Braunisch MC, Günthner R, et al. Renal and Skeletal Anomalies in a Cohort of Individuals With Clinically Presumed Hereditary Nephropathy Analyzed by Molecular Genetic Testing. Frontiers in genetics. 2021;12:642849. https://doi.org/10.3389/fgene.2021.642849.

Stokman MF, Lilien M, Knoers N. Nephronophthisis. In: Adam MP, Everman DB, Mirzaa GM, Pagon RA, Wallace SE, Bean LJH, et al., editors. GeneReviews(®). Seattle (WA): University of Washington, Seattle Copyright © 1993-2023, University of Washington, Seattle. GeneReviews is a registered trademark of the University of Washington, Seattle. All rights reserved.; 1993.

Strandberg AY, Khanfir H, Mäkimattila S, Saukkonen T, Strandberg TE, Hoti F. Insulins NPH, glargine, and detemir, and risk of severe hypoglycemia among working-age adults. Annals of medicine. 2017;49(4):357-64. https://doi.org/10.1080/07853890.2016.1278302.

Strong A, Li D, Mentch F, Hakonarson H. A novel heterotaxy gene: Expansion of the phenotype of TTC21B-spectrum disease. American journal of medical genetics Part A. 2021;185(4):1266-9. https://doi.org/10.1002/ajmg.a.62093.

Sundaresan P, Vijayalakshmi P, Thompson S, Ko AC, Fingert JH, Stone EM. Mutations that are a common cause of Leber congenital amaurosis in northern America are rare in southern India. Molecular vision. 2009;15:1781-7. https://pmc.ncbi.nlm.nih.gov/articles/PMC2742639/.

Sutoko S, Monden Y, Funane T, Tokuda T, Katura T, Sato H, et al. Erratum: Adaptive algorithm utilizing acceptance rate for eliminating noisy epochs in block-design functional near-infrared spectroscopy data: application to study in attention deficit/hyperactivity disorder children. Neurophotonics. 2018;5(4):049801. https://doi.org/10.1117/1.NPh.5.4.049801.

Svensson MJ, Lind I, Wirgart BZ, Östlund MR, Albert J. Performance of the Simplexa™ Flu A/B & RSV Direct Kit on respiratory samples collected in saline solution. Scandinavian journal of infectious diseases. 2014;46(12):825-31. https://doi.org/10.3109/00365548.2014.946444.

Syryn H, Hoorens A, Grammatikopoulos T, Deheragoda M, Symoens S, Vande Velde S, et al. Two cases of DCDC2-related neonatal sclerosing cholangitis with developmental delay and literature review. Clinical genetics. 2021;100(4):447-52. https://doi.org/10.1111/cge.14012.

Szypowska A, Golicki D, Groele L, Pańkowska E. Long-acting insulin analogue detemir compared with NPH insulin in type 1 diabetes: a systematic review and meta-analysis. Polskie Archiwum Medycyny Wewnetrznej. 2011;121(7-8):237-46. https://doi.org/10.20452/pamw.1065.

Tan K, Meiri A, Mowrey WB, Abbott R, Goodrich JT, Sandler AL, et al. Diffusion tensor imaging and ventricle volume quantification in patients with chronic shunt-treated hydrocephalus: a matched case-control study. Journal of neurosurgery. 2018;129(6):1611-22. https://doi.org/10.3171/2017.6.Jns162784.

Tan W, Lin A, Keppler-Noreuil K. Cranioectodermal Dysplasia. In: Adam MP, Everman DB, Mirzaa GM, Pagon RA, Wallace SE, Bean LJH, et al., editors. GeneReviews(®). Seattle (WA): University of Washington, Seattle Copyright © 1993-2023, University of Washington, Seattle. GeneReviews is a registered trademark of the University of Washington, Seattle. All rights reserved.; 1993.

Tanyel FC, Dağdeviren A, Müftüoğlu S, Gürsoy MH, Yürüker S, Büyükpamukçu N. Inguinal hernia revisited through comparative evaluation of peritoneum, processus vaginalis, and sacs obtained from children with hernia, hydrocele, and undescended testis. Journal of pediatric surgery. 1999;34(4):552-5. https://doi.org/10.1016/s0022-3468(99)90071-4.

Taylor CM, Hays NP, Emmett PM. Diet at Age 10 and 13 Years in Children Identified as Picky Eaters at Age 3 Years and in Children Who Are Persistent Picky Eaters in A Longitudinal Birth Cohort Study. Nutrients. 2019;11(4). https://doi.org/10.3390/nu11040807.

Taylor CM, Steer CD, Hays NP, Emmett PM. Growth and body composition in children who are picky eaters: a longitudinal view. European journal of clinical nutrition. 2019;73(6):869-78. https://doi.org/10.1038/s41430-018-0250-7.

Testa MA, Simonson DC. Satisfaction and quality of life with premeal inhaled versus injected insulin in adolescents and adults with type 1 diabetes. Diabetes care. 2007;30(6):1399-405. https://doi.org/10.2337/dc06-1497.

Thalange N, Bereket A, Larsen J, Hiort LC, Peterkova V. Treatment with insulin detemir or NPH insulin in children aged 2-5 yr with type 1 diabetes mellitus. Pediatric diabetes. 2011;12(7):632-41. https://doi.org/10.1111/j.1399-5448.2010.00750.x.

Thalange N, Bereket A, Larsen J, Hiort LC, Peterkova V. Insulin analogues in children with Type 1 diabetes: a 52-week randomized clinical trial. Diabetic medicine : a journal of the British Diabetic Association. 2013;30(2):216-25. https://doi.org/10.1111/dme.12041.

Tham LS, Schneck K, Ertekin A, Reviriego J. Modeling Pharmacokinetic Profiles of Insulin Regimens to Enhance Understanding of Subcutaneous Insulin Regimens. Journal of clinical pharmacology. 2017;57(9):1126-37. https://doi.org/10.1002/jcph.899.

Thomas CP, Gupta S, Freese ME, Chouhan KK, Dantuma MI, Holanda DG, et al. Sequential genetic testing of living-related donors for inherited renal disease to promote informed choice and enhance safety of living donation. Transplant international : official journal of the European Society for Organ Transplantation. 2021;34(12):2696-705. https://doi.org/10.1111/tri.14133.

Thompson SD, Coutts A, Craven CL, Toma AK, Thorne LW, Watkins LD. Elective ICP monitoring: how long is long enough? Acta neurochirurgica. 2017;159(3):485-90. https://doi.org/10.1007/s00701-016-3074-z.

Thun GA, Ferrarotti I, Imboden M, Rochat T, Gerbase M, Kronenberg F, et al. SERPINA1 PiZ and PiS heterozygotes and lung function decline in the SAPALDIA cohort. PLoS One. 2012;7(8):e42728. https://doi.org/10.1371/journal.pone.0042728.

Tian JH, Patel N, Haupt R, Zhou H, Weston S, Hammond H, et al. SARS-CoV-2 spike glycoprotein vaccine candidate NVX-CoV2373 immunogenicity in baboons and protection in mice. Nature communications. 2021;12(1):372. https://doi.org/10.1038/s41467-020-20653-8.

Tipene-Leach D, Pahau H, Joseph N, Coppell K, McAuley K, Booker C, et al. Insulin resistance in a rural Maori community. The New Zealand medical journal. 2004;117(1207):U1208. https://www.researchgate.net/publication/8120879_Insulin_resistance_in_a_rural_Maori_community.

Tisell M, Hellström P, Ahl-Börjesson G, Barrows G, Blomsterwall E, Tullberg M, et al. Long-term outcome in 109 adult patients operated on for hydrocephalus. British journal of neurosurgery. 2006;20(4):214-21. https://doi.org/10.1080/02688690600852324.

Tisell M, Höglund M, Wikkelsø C. National and regional incidence of surgery for adult hydrocephalus in Sweden. Acta neurologica Scandinavica. 2005;112(2):72-5. https://doi.org/10.1111/j.1600-0404.2005.00451.x.

Tokoro K, Chiba Y, Abe H, Tanaka N, Yamataki A, Kanno H. Importance of anti-siphon devices in the treatment of pediatric hydrocephalus. Child's nervous system : ChNS : official journal of the International Society for Pediatric Neurosurgery. 1994;10(4):236-8. https://doi.org/10.1007/bf00301160.

Traczewski W, Moskala M, Kruk D, Gościński I, Szwabowska D, Polak J, et al. The role of computerized rheoencephalography in the assessment of normal pressure hydrocephalus. Journal of neurotrauma. 2005;22(7):836-43. https://doi.org/10.1089/neu.2005.22.836.

Tsai JD, Lin SP, Huang FY, Hsu HC, Tsai TC. Juvenile nephronophthisis-medullary cystic disease complex: a family study. Zhonghua Minguo xiao er ke yi xue hui za zhi [Journal] Zhonghua Minguo xiao er ke yi xue hui. 1997;38(2):116-20.

Tsou CH, Cheng YC, Huang CY, Chen JH, Chen WH, Chai JW, et al. Using deep learning convolutional neural networks to automatically perform cerebral aqueduct CSF flow analysis. Journal of clinical neuroscience : official journal of the Neurosurgical Society of Australasia. 2021;90:60-7. https://doi.org/10.1016/j.jocn.2021.05.010.

Tsunoda A, Mitsuoka H, Sato K, Kanayama S. A quantitative index of intracranial cerebrospinal fluid distribution in normal pressure hydrocephalus using an MRI-based processing technique. Neuroradiology. 2000;42(6):424-9. https://doi.org/10.1007/s002349900241.

Ubeyli ED, Ilbay K, Ilbay G, Sahin D, Akansel G. Differentiation of two subtypes of adult hydrocephalus by mixture of experts. Journal of medical systems. 2010;34(3):281-90. https://doi.org/10.1007/s10916-008-9239-4.

Umpierrez GE, Hor T, Smiley D, Temponi A, Umpierrez D, Ceron M, et al. Comparison of inpatient insulin regimens with detemir plus aspart versus neutral protamine hagedorn plus regular in medical patients with type 2 diabetes. The Journal of clinical endocrinology and metabolism. 2009;94(2):564-9. https://doi.org/10.1210/jc.2008-1441.

Urakami T, Morimoto S, Kubota S, Funaki S, Harada K. Usefulness of the long-acting insulin analogue glargine in basal-bolus therapy for Japanese children and adolescents with type 1 diabetes mellitus. Journal of pediatric endocrinology & metabolism : JPEM. 2007;20(7):807-15. https://doi.org/10.1515/jpem.2007.20.7.807.

Usami T, Okita K, Shimane T, Matsumoto T. Comparison of patients with benzodiazepine receptor agonist-related psychiatric disorders and over-the-counter drug-related psychiatric disorders before and after the COVID-19 pandemic: Changes in psychosocial characteristics and types of abused drugs. Neuropsychopharmacol Rep. 2024;44(2):437-46. https://doi.org/10.1002/npr2.12440.

Ushewokunze S, Haja Mydin HN, Prasad R, Mendelow AD. Lumbar subcutaneous shunt: a novel technique for therapeutic decision making in normal pressure hydrocephalus (NPH) and benign intracranial hypertension (BIH). British journal of neurosurgery. 2008;22(5):678-81. https://doi.org/10.1080/02688690802007883.

Utsch B, Sayer JA, Attanasio M, Pereira RR, Eccles M, Hennies HC, et al. Identification of the first AHI1 gene mutations in nephronophthisis-associated Joubert syndrome. Pediatr Nephrol. 2006;21(1):32-5. https://doi.org/10.1007/s00467-005-2054-y.

Valente EM, Salpietro DC, Brancati F, Bertini E, Galluccio T, Tortorella G, et al. Description, nomenclature, and mapping of a novel cerebello-renal syndrome with the molar tooth malformation. Am J Hum Genet. 2003;73(3):663-70. https://doi.org/10.1086/378241.

van Avendonk MJ, Rutten GE. Insulin therapy in type 2 diabetes: what is the evidence? Diabetes, obesity & metabolism. 2009;11(5):415-32. https://doi.org/10.1111/j.1463-1326.2008.00981.x.

van Golen LW, IJzerman RG, Huisman MC, Hensbergen JF, Hoogma RP, Drent ML, et al. Cerebral blood flow and glucose metabolism in appetite-related brain regions in type 1 diabetic patients after treatment with insulin detemir and NPH insulin: a randomized controlled crossover trial. Diabetes care. 2013;36(12):4050-6. https://doi.org/10.2337/dc13-0093.

Vignati L, Anderson JH, Jr., Iversen PW. Efficacy of insulin lispro in combination with NPH human insulin twice per day in patients with insulin-dependent or non-insulin-dependent diabetes mellitus. Multicenter Insulin Lispro Study Group. Clinical therapeutics. 1997;19(6):1408-21. https://doi.org/10.1016/s0149-2918(97)80014-8.

Vollbach H, Auzanneau M, Reinehr T, Wiegand S, Schwab KO, Oeverink R, et al. Choice of basal insulin therapy is associated with weight and height development in type 1 diabetes: A multicenter analysis from the German/Austrian DPV registry in 10 338 children and adolescents. Journal of diabetes. 2021;13(11):930-9. https://doi.org/10.1111/1753-0407.13207.

Wadsworth HE, Horton DK, Dhima K, Cullum CM, White J, Ruchinskas R. Change in Balance and Neuropsychological Measures Post-Lumbar Drain Trial in Patients with Suspected Normal Pressure Hydrocephalus. Dementia and geriatric cognitive disorders. 2021;50(6):529-34. https://doi.org/10.1159/000520693.

Wahadat MJ, Schonenberg-Meinema D, van Helden-Meeuwsen CG, van Tilburg SJ, Groot N, Schatorjé EJH, et al. Gene signature fingerprints stratify SLE patients in groups with similar biological disease profiles: a multicentre longitudinal study. Rheumatology (Oxford, England). 2022;61(11):4344-54. https://doi.org/10.1093/rheumatology/keac083.

Waki N, Memon A, Khan MO, Faruqi H, Masood S, Rauf M, et al. Current understanding of treatment and management protocol for adult diabetic in-patients at a tertiary care hospital. JPMA The Journal of the Pakistan Medical Association. 2012;62(5):520-4. https://www.archive.jpma.org.pk/article-details/3418.

Wang F, Carabino JM, Vergara CM. Insulin glargine: a systematic review of a long-acting insulin analogue. Clinical therapeutics. 2003;25(6):1541-77, discussion 39-40. https://doi.org/10.1016/s0149-2918(03)80156-x.

Wang W, Wang M, Li Z, Wang T, Da R. The intraocular pressure could not be used to determine the intracranial pressure in patients with hydrocephalus. The International journal of neuroscience. 2019;129(1):42-8. https://doi.org/10.1080/00207454.2018.1486832.

Ware N, Sebire NJ, Chong WK, Krishnan R, Marks SD. When is biopsy-proven TIN not simply TIN? Answers. Pediatr Nephrol. 2017;32(6):977-9. https://doi.org/10.1007/s00467-016-3478-2.

Ware N, Sebire NJ, Chong WK, Krishnan R, Marks SD. When is biopsy-proven TIN not simply TIN? Questions. Pediatr Nephrol. 2017;32(6):975-6. https://doi.org/10.1007/s00467-016-3465-7.

Weiner HL, Constantini S, Cohen H, Wisoff JH. Current treatment of normal-pressure hydrocephalus: comparison of flow-regulated and differential-pressure shunt valves. Neurosurgery. 1995;37(5):877-84. https://doi.org/10.1227/00006123-199511000-00005.

Wen JW, Furth SL, Ruebner RL. Kidney and liver transplantation in children with fibrocystic liver-kidney disease: data from the US Scientific Registry of Transplant Recipients: 1990-2010. Pediatr Transplant. 2014;18(7):726-32. https://doi.org/10.1111/petr.12330.

Wheeler MD, Barrientos-Perez M, Lo FS, Liang B, Lunsford A, Thórisdóttir Ó, et al. A 26-week, randomized trial of insulin detemir versus NPH insulin in children and adolescents with type 2 diabetes (iDEAt2). European journal of pediatrics. 2018;177(10):1497-503. https://doi.org/10.1007/s00431-018-3205-z.

White NH, Chase HP, Arslanian S, Tamborlane WV. Comparison of glycemic variability associated with insulin glargine and intermediate-acting insulin when used as the basal component of multiple daily injections for adolescents with type 1 diabetes. Diabetes care. 2009;32(3):387-93. https://doi.org/10.2337/dc08-0800.

Witthaus E, Stewart J, Bradley C. Treatment satisfaction and psychological well-being with insulin glargine compared with NPH in patients with Type 1 diabetes. Diabetic medicine : a journal of the British Diabetic Association. 2001;18(8):619-25. https://doi.org/10.1046/j.1464-5491.2001.00529.x.

Wolever TM, Hamad S, Chiasson JL, Josse RG, Leiter LA, Rodger NW, et al. Day-to-day consistency in amount and source of carbohydrate intake associated with improved blood glucose control in type 1 diabetes. Journal of the American College of Nutrition. 1999;18(3):242-7. https://doi.org/10.1080/07315724.1999.10718858.

Wolfsdorf JI, Laffel LM, Pasquarello C, Vernon A, Herskowitz RD. Split-mixed insulin regimen with human ultralente before supper and NPH (isophane) before breakfast in children and adolescents with IDDM. Diabetes care. 1991;14(11):1100-6. https://doi.org/10.2337/diacare.14.11.1100.

Xiao H, Hildebrandt F. Whole exome sequencing identifies monogenic forms of nephritis in a previously unsolved cohort of children with steroid-resistant nephrotic syndrome and hematuria. Pediatr Nephrol. 2022;37(7):1567-74. https://doi.org/10.1007/s00467-021-05312-4.

Yagasaki H, Kobayashi K, Saitou T, Nagamine K, Mitsui Y, Mochizuki M, et al. Nocturnal blood glucose and IGFBP-1 changes in type 1 diabetes: Differences in the dawn phenomenon between insulin regimens. Experimental and clinical endocrinology & diabetes : official journal, German Society of Endocrinology [and] German Diabetes Association. 2010;118(3):195-9. https://doi.org/10.1055/s-0029-1239518.

Yamada N, Iwasa H, Mori S, Kurokawa N, Fujimoto K, Kawashima K, et al. Melatonin secretion in normal pressure hydrocephalus after cerebral aneurysm rupture--investigation before and after ventriculoperitoneal shunt. Neurologia medico-chirurgica. 1991;31(8):490-7. https://doi.org/10.2176/nmc.31.490.

Yamauchi T, Imamura M, Takasawa K, Nakajima K, Nakagawa R, Gau M, et al. Prematurity at less than 24 weeks of gestation is a risk for prolonged hyperglycemia in extremely low-birth weight infants. Endocrine. 2020;70(1):71-7. https://doi.org/10.1007/s12020-020-02393-3.

Yang J, Dombrowski SM, Krishnan C, Krajcir N, Deshpande A, El-Khoury S, et al. Vascular endothelial growth factor in the CSF of elderly patients with ventriculomegaly: variability, periodicity and levels in drainage responders and non-responders. Clinical neurology and neurosurgery. 2013;115(9):1729-34. https://doi.org/10.1016/j.clineuro.2013.03.017.

Yao T, Udwan K, John R, Rana A, Haghighi A, Xu L, et al. Integration of Genetic Testing and Pathology for the Diagnosis of Adults with FSGS. Clin J Am Soc Nephrol. 2019;14(2):213-23. https://doi.org/10.2215/cjn.08750718.

Yashikhina A, Romanov D, Strelnik S, Gradinar A, Markina E, Kuvshinova N, et al. Non-Psychiatrist Healthcare Professionals? Attitudes Toward Patients with Mental Disorders: Lower Scores in Social Distance as a Fragile Facet of Public Stigma Against Depression. Psychiatria Danubina. 2022;34(Suppl 8):238-45. https://www.psychiatria-danubina.com/UserDocsImages/pdf/dnb_vol34_noSuppl%208/dnb_vol34_noSuppl%208_238.pdf.

Yatsyshina S, Mayanskiy N, Shipulina O, Kulichenko T, Alyabieva N, Katosova L, et al. Detection of respiratory pathogens in pediatric acute otitis media by PCR and comparison of findings in the middle ear and nasopharynx. Diagnostic microbiology and infectious disease. 2016;85(1):125-30. https://doi.org/10.1016/j.diagmicrobio.2016.02.010.

Young S, Leodoro B, Toukune A, Ala R, Bissett I, Windsor JA, et al. Patient-Reported Barriers to Accessing Surgical Care in Northern Vanuatu. World journal of surgery. 2019;43(12):2979-85. https://doi.org/10.1007/s00268-019-05146-0.

Yücel MA, Lühmann AV, Scholkmann F, Gervain J, Dan I, Ayaz H, et al. Errata: Best practices for fNIRS publications. Neurophotonics. 2021;8(1):019802. https://doi.org/10.1117/1.NPh.8.1.019802.

Zaucke F, Boehnlein JM, Steffens S, Polishchuk RS, Rampoldi L, Fischer A, et al. Uromodulin is expressed in renal primary cilia and UMOD mutations result in decreased ciliary uromodulin expression. Human molecular genetics. 2010;19(10):1985-97. https://doi.org/10.1093/hmg/ddq077.

Zdarska DJ, Kvapil M, Rusavy Z, Krcma M, Broz J, Krivska B, et al. Comparison of glucose variability assessed by a continuous glucose-monitoring system in patients with type 2 diabetes mellitus switched from NPH insulin to insulin glargine: the COBIN2 study. Wiener klinische Wochenschrift. 2014;126(7-8):228-37. https://doi.org/10.1007/s00508-014-0508-6.

Zemack G, Romner B. Adjustable valves in normal-pressure hydrocephalus: a retrospective study of 218 patients. Neurosurgery. 2002;51(6):1392-400; discussion 400-2. https://doi.org/10.1227/01.neu.0000316272.28209.af.

Zhang H, Barner JC, Moczygemba LR, Rascati KL. Assessment of basal insulin adherence using 2 methodologies among Texas Medicaid enrollees with type 2 diabetes. Journal of managed care & specialty pharmacy. 2020;26(11):1434-44. https://doi.org/10.18553/jmcp.2020.26.11.1434.

Zhang L, Yang W, Ying D, Cherny SS, Hildebrandt F, Sham PC, et al. Homozygosity mapping on a single patient: identification of homozygous regions of recent common ancestry by using population data. Human mutation. 2011;32(3):345-53. https://doi.org/10.1002/humu.21432.

Zhou W, Otto EA, Cluckey A, Airik R, Hurd TW, Chaki M, et al. FAN1 mutations cause karyomegalic interstitial nephritis, linking chronic kidney failure to defective DNA damage repair. Nature genetics. 2012;44(8):910-5. https://doi.org/10.1038/ng.2347.

Zhu H, Zhao ZH, Zhu SY, Xiong F, He LH, Zhang Y, et al. Renal-hepatic-pancreatic dysplasia-1 with a novel NPHP3 genotype: a case report and review of the literature. BMC pediatrics. 2022;22(1):603. https://doi.org/10.1186/s12887-022-03659-7.

Zinman B. Basal insulin replacement and use of rapid-acting insulin analogues in patients with type 1 diabetes. Endocrine practice : official journal of the American College of Endocrinology and the American Association of Clinical Endocrinologists. 2000;6(1):88-92. https://doi.org/10.4158/ep.6.1.88.

Online Resource 4: List of included studies

Ala-Mello S, Jääskeläinen J, Koskimies O. Familial juvenile nephronophthisis:An ultrasonographic follow-up of seven patients. Acta Radiologica. 1998;39(1):84-9. https://doi.org/10.1080/02841859809172156.

Ala-Mello S, Kivivuori SM, Rönnholm KAR, Koskimies O, Siimes MA. Mechanism underlying early anaemia in children with familial juvenile nephronophthisis. Pediatric Nephrology. 1996;10(5):578-81. https://doi.org/10.1007/s004670050164.

Ala-Mello S, Koskimies O, Rapola J, Kääriäinen H. Nephronophthisis in Finland: epidemiology and comparison of genetically classified subgroups. European Journal of Human Genetics. 1999;7(2):205-11. https://doi.org/10.1038/sj.ejhg.5200268.

Antignac C, Arduy CH, Beckmann JS, Benessy F, Gros F, Medhioub M, et al. A gene for familial juvenile nephronophthisis (recessive medullary cystic kidney disease) maps to chromosome 2p. Nature genetics. 1993;3(4):342-5. https://doi.org/10.1038/ng0493-342.

Attanasio M, Uhlenhaut NH, Sousa VH, O'Toole JF, Otto E, Anlag K, et al. Loss of GLIS2 causes nephronophthisis in humans and mice by increased apoptosis and fibrosis. Nature genetics. 2007;39(8):1018-24. https://doi.org/10.1038/ng2072.

Avcı B, Baskın E, Gülleroğlu K, Yılmaz AC, Kantar A, Akdur A, et al. Long-term outcomes of kidney transplant recipients with juvenile nephronophthisi. Experimental and Clinical Transplantation. 2022;20(5):122-5. https://doi.org/10.6002/ect.PediatricSymp2022.O39.

Bakkaloğlu SA, Kandur Y, Bedir-Demirdağ T, Işık-Gönül İ, Hildebrandt F. Diverse phenotypic expression of NPHP4 mutations in four siblings. The Turkish journal of pediatrics. 2014;56(4):423-6. https://turkjpediatr.org/article/view/1393/1386.

Baris H, Bejjani BA, Tan W-H, Coulter DL, Martin JA, Storm AL, et al. Identification of a novel polymorphism—the duplication of the NPHP1 (nephronophthisis 1) gene. American Journal of Medical Genetics Part A. 2006;140A(17):1876-9. https://doi.org/10.1002/ajmg.a.31390.

Beal F, Forrester N, Watson E, Williams M, Buckton A, Marlais M, et al. A targeted gene panel illuminates pathogenesis in young people with unexplained kidney failure. Journal of nephrology. 2024;37(5):1273-84. https://doi.org/10.1007/s40620-024-01964-1.

Birtel J, Spital G, Book M, Habbig S, Bäumner S, Riehmer V, et al. NPHP1 gene-associated nephronophthisis is associated with an occult retinopathy. Kidney International. 2021;100(5):1092-100. https://doi.org/10.1016/j.kint.2021.06.012.

Bizet AA, Becker-Heck A, Ryan R, Weber K, Filhol E, Krug P, et al. Mutations in TRAF3IP1/IFT54 reveal a new role for IFT proteins in microtubule stabilization. Nature communications. 2015;6(1):8666. https://doi.org/10.1038/ncomms9666.

Blowey DL, Querfeld U, Geary D, Warady BA, Alon U. Ultrasound findings in juvenile nephronophthisis. Pediatric Nephrology. 1996;10(1):22-4. https://doi.org/10.1007/BF00863431.

Braun DA, Schueler M, Halbritter J, Gee HY, Porath JD, Lawson JA, et al. Whole exome sequencing identifies causative mutations in the majority of consanguineous or familial cases with childhood-onset increased renal echogenicity. Kidney International. 2016;89(2):468-75. https://doi.org/10.1038/ki.2015.317.

Caridi G, Dagnino M, Gusmano R, Ginevri F, Murer L, Ghio L, et al. Clinical and molecular heterogeneity of juvenile nephronophthisis in Italy: Insights from molecular screening. American Journal of Kidney Diseases. 2000;35(1):44-51. https://doi.org/10.1016/S0272-6386(00)70300-3.

Caridi G, Dagnino M, Rossi A, Valente EM, Bertini E, Fazzi E, et al. Nephronophthisis type 1 deletion syndrome with neurological symptoms: Prevalence and significance of the association. Kidney International. 2006;70(7):1342-7. https://doi.org/10.1038/sj.ki.5001768.

Caridi G, Murer L, Bellantuono R, Sorino P, Caringella DA, Gusmano R, et al. Renal-retinal syndromes: Association of retinal anomalies and recessive nephronophthisis in patients with homozygous deletion of the NPH1 locus. American Journal of Kidney Diseases. 1998;32(6):1059-62. https://doi.org/10.1016/S0272-6386(98)70083-6.

Chaari I, Trabelsi M, Goucha R, Elaribi Y, Kharrat M, Guarguah T, et al. Prevalence and incidence estimation of large NPHP1 homozygous deletion in Tunisian population. Pathologie Biologie. 2012;60(6):e84-e6. https://doi.org/10.1016/j.patbio.2012.05.003.

Chaki M, Airik R, Ghosh Amiya K, Giles Rachel H, Chen R, Slaats Gisela G, et al. Exome Capture Reveals ZNF423 and CEP164 Mutations, Linking Renal Ciliopathies to DNA Damage Response Signaling. Cell. 2012;150(3):533-48. https://doi.org/10.1016/j.cell.2012.06.028.

Chaki M, Hoefele J, Allen SJ, Ramaswami G, Janssen S, Bergmann C, et al. Genotype–phenotype correlation in 440 patients with NPHP-related ciliopathies. Kidney International. 2011;80(11):1239-45. https://doi.org/10.1038/ki.2011.284.

Chen W, Wang F, Zeng W, Zhang X, Shen L, Zhang Y, et al. Biallelic mutations of TTC12 and TTC21B were identified in Chinese patients with multisystem ciliopathy syndromes. Human Genomics. 2022;16(1):48. https://doi.org/10.1186/s40246-022-00421-z.

Dahmer-Heath M, Schriever V, Kollmann S, Schleithoff C, Titieni A, Cetiner M, et al. Systematic evaluation of olfaction in patients with hereditary cystic kidney diseases/renal ciliopathies. Journal of Medical Genetics. 2021;58(9):629-36. https://doi.org/10.1136/jmedgenet-2020-107192.

Doreille A, Raymond L, Lebre A-S, Linster C, Saraeva Lamri R, Karras A, et al. Nephronophthisis in Young Adults Phenocopying Thrombotic Microangiopathy and Severe Nephrosclerosis. Clinical Journal of the American Society of Nephrology. 2021;16(4):615-7. https://doi.org/10.2215/cjn.11890720.

Failler M, Gee HY, Krug P, Joo K, Halbritter J, Belkacem L, et al. Mutations of CEP83 cause infantile nephronophthisis and intellectual disability. Am J Hum Genet. 2014;94(6):905-14. https://doi.org/10.1016/j.ajhg.2014.05.002.

Gheissari A, Harandavar M, Hildebrandt F, Braun DA, Sedghi M, Parsi N, et al. Gene mutation analysis in Iranian children with nephronophthisis: a two-center study. Iran J Kidney Dis. 2015;9(2):119-25. https://www.ijkd.org/index.php/ijkd/article/view/1713/756.

Gjerstad AC, Skrunes R, Tøndel C, Åsberg A, Leh S, Klingenberg C, et al. Kidney biopsy diagnosis in childhood in the Norwegian Kidney Biopsy Registry and the long-term risk of kidney replacement therapy: a 25-year follow-up. Pediatric Nephrology. 2023;38(4):1249-56. https://doi.org/10.1007/s00467-022-05706-y.

Green A, Allos M, Donohoe J, Carmody M, Walshe J. Prevalence of Hereditary Renal-Disease. Irish Med J. 1990;83(1):11-3. <Go to ISI>://WOS:A1990DB11600008.

Grenda R, Wühl E, Litwin M, Janas R, Śladowska J, Arbeiter K, et al. Urinary excretion of endothelin-1 (ET-1), transforming growth factor-β1 (TGF-β1) and vascular endothelial growth factor (VEGF 165) in paediatric chronic kidney diseases: results of the ESCAPE trial. Nephrology Dialysis Transplantation. 2007;22(12):3487-94. https://doi.org/10.1093/ndt/gfm300.

Gretz N, Schärer K, Waldherr R, Strauch M. Rate of deterioration of renal function in juvenile nephronophthisis. Pediatric Nephrology. 1989;3(1):56-60. https://doi.org/10.1007/BF00859627.

Haider NB, Carmi R, Shalev H, Sheffield VC, Landau D. A Bedouin Kindred with Infantile Nephronophthisis Demonstrates Linkage to Chromosome 9 by Homozygosity Mapping. The American Journal of Human Genetics. 1998;63(5):1404-10. https://doi.org/10.1086/302108.

Halbritter J, Diaz K, Chaki M, Porath JD, Tarrier B, Fu C, et al. High-throughput mutation analysis in patients with a nephronophthisis-associated ciliopathy applying multiplexed barcoded array-based PCR amplification and next-generation sequencing. Journal of Medical Genetics. 2012;49(12):756-67. https://doi.org/10.1136/jmedgenet-2012-100973.

Halbritter J, Porath JD, Diaz KA, Braun DA, Kohl S, Chaki M, et al. Identification of 99 novel mutations in a worldwide cohort of 1,056 patients with a nephronophthisis-related ciliopathy. Human genetics. 2013;132(8):865-84. https://doi.org/10.1007/s00439-013-1297-0.

Hamiwka LA, Midgley JP, Wade AW, Martz KL, Grisaru S. Outcomes of kidney transplantation in children with nephronophthisis: An analysis of the North American Pediatric Renal Trials and Collaborative Studies (NAPRTCS) Registry. Pediatric Transplantation. 2008;12(8):878-82. https://doi.org/10.1111/j.1399-3046.2008.00942.x.

Hildebrandt F, Strahm B, Nothwang H-G, Gretz N, Schnieders B, Singh-Sawhney I, et al. Molecular genetic identification of families with juvenile nephronophthisis type 1: Rate of progression to renal failure. Kidney International. 1997;51(1):261-9. https://doi.org/10.1038/ki.1997.31.

Hoefele J, Nayir A, Chaki M, Imm A, Allen SJ, Otto EA, et al. Pseudodominant inheritance of nephronophthisis caused by a homozygous NPHP1 deletion. Pediatr Nephrol. 2011;26(6):967-71. https://doi.org/10.1007/s00467-011-1761-9.

Hoefele J, Sudbrak R, Reinhardt R, Lehrack S, Hennig S, Imm A, et al. Mutational analysis of the NPHP4 gene in 250 patients with nephronophthisis. Human mutation. 2005;25(4):411-. https://doi.org/10.1002/humu.9326.

Hoefele J, Wolf MTF, O'Toole JF, Otto EA, Schultheiss U, Deschenes G, et al. Evidence of Oligogenic Inheritance in Nephronophthisis. Journal of the American Society of Nephrology. 2007;18(10). https://journals.lww.com/jasn/fulltext/2007/10000/evidence_of_oligogenic_inheritance_in.23.aspx.

Hoff S, Halbritter J, Epting D, Frank V, Nguyen TM, van Reeuwijk J, et al. ANKS6 is a central component of a nephronophthisis module linking NEK8 to INVS and NPHP3. Nature genetics. 2013;45(8):951-6. https://doi.org/10.1038/ng.2681.

Hudson R, Patel C, Hawley CM, O'Shea S, Snelling P, Ho G, et al. Adult-Diagnosed Nonsyndromic Nephronophthisis in Australian Families Caused by Biallelic NPHP4 Variants. American Journal of Kidney Diseases. 2020;76(2):282-7. https://doi.org/10.1053/j.ajkd.2019.08.031.

Hussain S, Akhtar N, Qamar R, Khan N, Naeem M. Molecular Study of Nephronophthisis in 7 Unrelated Pakistani Families. Iran J Kidney Dis. 2018;12(4):240-2. http://www.ijkd.org/index.php/ijkd/article/view/3804/1017.

Kang HG, Ahn YH, Kim JH, Ha I-S, Yu YS, Park Y-H, et al. Atypical retinopathy in patients with nephronophthisis type 1: an uncommon ophthalmological finding. Clinical & Experimental Ophthalmology. 2015;43(5):437-42. https://doi.org/10.1111/ceo.12469.

König JC, Karsay R, Gerß J, Schlingmann KP, Dahmer-Heath M, Telgmann AK, et al. Refining Kidney Survival in 383 Genetically Characterized Patients With Nephronophthisis. Kidney international reports. 2022;7(9):2016–28. https://doi.org/10.1016/j.ekir.2022.05.035.

König JC, Kranz B, König S, Schlingmann KP, Titieni A, Tönshoff B, et al. Phenotypic Spectrum of Children with Nephronophthisis and Related Ciliopathies. Clinical journal of the American Society of Nephrology : CJASN. 2017;12(12):1974–83. https://doi.org/10.2215/cjn.01280217.

Lee JM, Ahn YH, Kang HG, Ha IIS, Lee K, Moon KC, et al. Nephronophthisis 13: implications of its association with Caroli disease and altered intracellular localization of WDR19 in the kidney. Pediatric Nephrology. 2015;30(9):1451-8. https://doi.org/10.1007/s00467-015-3068-8.

Li J, Su X, Zhang H, Wu W, Li J, Chen Y, et al. Genotype and phenotype analysis and transplantation strategy in children with kidney failure caused by NPHP. Pediatric Nephrology. 2023;38(5):1609-20. https://doi.org/10.1007/s00467-022-05763-3.

Macia MS, Halbritter J, Delous M, Bredrup C, Gutter A, Filhol E, et al. Mutations in MAPKBP1 Cause Juvenile or Late-Onset Cilia-Independent Nephronophthisis. The American Journal of Human Genetics. 2017;100(2):323-33. https://doi.org/10.1016/j.ajhg.2016.12.011.

Mashat SD, El-Desoky SM, Abdulaziz Kari J. Outcome of Multi-Cystic Dysplastic Kidneys in Children. Iranian journal of pediatrics. 2015;25(5):e2991. https://doi.org/10.5812/ijp.2991.

Medhioub M, Cherif D, Benessy F, Silbermann F, Gubler MC, Le Paslier D, et al. Refined Mapping of a Gene (NPH1) Causing Familial Juvenile Nephronophthisis and Evidence for Genetic Heterogeneity. Genomics. 1994;22(2):296-301. https://doi.org/10.1006/geno.1994.1387.

Mehrkash M, Golestaneh SJ, Madihi Y, Paknazar F, Hadian M, Akbari M, et al. Pruritus Features in Children with End-Stage Renal Disease Underwent Dialysis: A Cross-Sectional Study. Int J Pediatr. 2021;2021:9970321. https://doi.org/10.1155/2021/9970321.

Mistry K, Ireland JHE, Ng RCK, Henderson JM, Pollak MR. Novel Mutations in NPHP4 in a Consanguineous Family With Histological Findings of Focal Segmental Glomerulosclerosis. American Journal of Kidney Diseases. 2007;50(5):855-64. https://doi.org/10.1053/j.ajkd.2007.08.009.

Murer L, Caridi G, Della Vella M, Montini G, Carasi C, Ghiggeri G, et al. Expression of Nuclear Transcription Factor PAX2 in Renal Biopsies of Juvenile Nephronophthisis. Nephron. 2002;91(4):588-93. https://doi.org/10.1159/000065017.

Nyberg G, Friman S, Svalander C, Nordén G. Spectrum of hereditary renal disease in a kidney transplant population. Nephrol Dial Transplant. 1995;10(6):859-65. https://doi.org/10.1093/ndt/10.6.859.

O'Toole JF, Liu Y, Davis EE, Westlake CJ, Attanasio M, Otto EA, et al. Individuals with mutations in XPNPEP3, which encodes a mitochondrial protein, develop a nephronophthisis-like nephropathy. The Journal of Clinical Investigation. 2010;120(3):791-802. https://doi.org/10.1172/JCI40076.

Obeidova L, Seeman T, Fencl F, Blahova K, Hojny J, Elisakova V, et al. Results of targeted next-generation sequencing in children with cystic kidney diseases often change the clinical diagnosis. PLoS One. 2020;15(6):e0235071. https://doi.org/10.1371/journal.pone.0235071.

Olbrich H, Fliegauf M, Hoefele J, Kispert A, Otto E, Volz A, et al. Mutations in a novel gene, NPHP3, cause adolescent nephronophthisis, tapeto-retinal degeneration and hepatic fibrosis. Nature genetics. 2003;34(4):455-9. https://doi.org/10.1038/ng1216.

Olinger E, Alawi IA, Al Riyami MS, Salmi IA, Molinari E, Faqeih EA, et al. A discarded synonymous variant in NPHP3 explains nephronophthisis and congenital hepatic fibrosis in several families. Human mutation. 2021;42(10):1221-8. https://doi.org/10.1002/humu.24251.

Omran H, Fernandez C, Jung M, Häffner K, Fargier B, Villaquiran A, et al. Identification of a New Gene Locus for Adolescent Nephronophthisis, on Chromosome 3q22 in a Large Venezuelan Pedigree. The American Journal of Human Genetics. 2000;66(1):118-27. https://doi.org/10.1086/302705.

Omran H, Häffner K, Vollmer M, Pigulla J, Wagner G, Caridi G, et al. Exclusion of the candidate genes ACE and Bcl-2 for six families with nephronophthisis not linked to the NPH1 locus. Nephrology Dialysis Transplantation. 1999;14(10):2328-31. https://doi.org/10.1093/ndt/14.10.2328.

Otto EA, Helou J, Allen SJ, O'Toole JF, Wise EL, Ashraf S, et al. Mutation analysis in nephronophthisis using a combined approach of homozygosity mapping, CEL I endonuclease cleavage, and direct sequencing. Human mutation. 2008;29(3):418-26. https://doi.org/10.1002/humu.20669.

Otto EA, Hurd TW, Airik R, Chaki M, Zhou W, Stoetzel C, et al. Candidate exome capture identifies mutation of SDCCAG8 as the cause of a retinal-renal ciliopathy. Nature genetics. 2010;42(10):840-50. https://doi.org/10.1038/ng.662.

Otto EA, Loeys B, Khanna H, Hellemans J, Sudbrak R, Fan S, et al. Nephrocystin-5, a ciliary IQ domain protein, is mutated in Senior-Loken syndrome and interacts with RPGR and calmodulin. Nature genetics. 2005;37(3):282-8. https://doi.org/10.1038/ng1520.

Otto EA, Ramaswami G, Janssen S, Chaki M, Allen SJ, Zhou W, et al. Mutation analysis of 18 nephronophthisis associated ciliopathy disease genes using a DNA pooling and next generation sequencing strategy. Journal of Medical Genetics. 2011;48(2):105. https://doi.org/10.1136/jmg.2010.082552.

Otto EA, Schermer B, Obara T, O'Toole JF, Hiller KS, Mueller AM, et al. Mutations in INVS encoding inversin cause nephronophthisis type 2, linking renal cystic disease to the function of primary cilia and left-right axis determination. Nature genetics. 2003;34(4):413-20. https://doi.org/10.1038/ng1217.

Otto EA, Tory K, Attanasio M, Zhou W, Chaki M, Paruchuri Y, et al. Hypomorphic mutations in meckelin (MKS3/TMEM67) cause nephronophthisis with liver fibrosis (NPHP11). Journal of Medical Genetics. 2009;46(10):663. https://doi.org/10.1136/jmg.2009.066613.

Parisi MA, Bennett CL, Eckert ML, Dobyns WB, Gleeson JG, Shaw DWW, et al. The NPHP1 Gene Deletion Associated with Juvenile Nephronophthisis Is Present in a Subset of Individuals with Joubert Syndrome. The American Journal of Human Genetics. 2004;75(1):82-91. https://doi.org/10.1086/421846.

Petzold F, Billot K, Chen X, Henry C, Filhol E, Martin Y, et al. The genetic landscape and clinical spectrum of nephronophthisis and related ciliopathies. Kidney Int. 2023;104(2):378-87. https://doi.org/10.1016/j.kint.2023.05.007.

Qiu Y-L, Wang L, Huang M, Lian M, Wang F, Gong Y, et al. Association of novel TMEM67 variants with mild phenotypes of high gamma-glutamyl transpeptidase cholestasis and congenital hepatic fibrosis. Journal of Cellular Physiology. 2022;237(6):2713-23. https://doi.org/10.1002/jcp.30788.

Sakakibara N, Nozu K, Yamamura T, Horinouchi T, Nagano C, Ye MJ, et al. Comprehensive genetic analysis using next-generation sequencing for the diagnosis of nephronophthisis-related ciliopathies in the Japanese population. Journal of Human Genetics. 2022;67(7):427-40. https://doi.org/10.1038/s10038-022-01020-5.

Sayer JA, Otto EA, O'Toole JF, Nurnberg G, Kennedy MA, Becker C, et al. The centrosomal protein nephrocystin-6 is mutated in Joubert syndrome and activates transcription factor ATF4. Nature genetics. 2006;38(6):674-81. https://doi.org/10.1038/ng1786.

Schuermann MJ, Otto E, Becker A, Saar K, Rüschendorf F, Polak BC, et al. Mapping of Gene Loci for Nephronophthisis Type 4 and Senior-Løken Syndrome, to Chromosome 1p36. The American Journal of Human Genetics. 2002;70(5):1240-6. https://doi.org/10.1086/340317.

Shaheen R, Szymanska K, Basu B, Patel N, Ewida N, Faqeih E, et al. Characterizing the morbid genome of ciliopathies. Genome Biology. 2016;17(1):242. https://doi.org/10.1186/s13059-016-1099-5.

Snoek R, van Setten J, Keating BJ, Israni AK, Jacobson PA, Oetting WS, et al. NPHP1 (Nephrocystin-1) Gene Deletions Cause Adult-Onset ESRD. Journal of the American Society of Nephrology. 2018;29(6). https://journals.lww.com/jasn/fulltext/2018/06000/nphp1__nephrocystin_1__gene_deletions_cause.24.aspx.

Soliman NA, Hildebrandt F, Allen SJ, Otto EA, Nabhan MM, Badr AM. Homozygous NPHP1 deletions in Egyptian children with nephronophthisis including an infantile onset patient. Pediatric Nephrology. 2010;25(10):2193-4. https://doi.org/10.1007/s00467-010-1539-5.

Soliman NA, Hildebrandt F, Otto EA, Nabhan MM, Allen SJ, Badr AM, et al. Clinical characterization and NPHP1 mutations in nephronophthisis and associated ciliopathies: a single center experience. Saudi J Kidney Dis Transpl. 2012;23(5):1090-8. https://doi.org/10.4103/1319-2442.100968.

Soliman NA, Nabhan MM, Bazaraa HM, Badr AM, Shaheen M. Clinical and ultrasonographical characterization of childhood cystic kidney diseases in Egypt. Renal Failure. 2014;36(5):694-700. https://doi.org/10.3109/0886022X.2014.883996.

Stokman MF, Bijnsdorp IV, Schelfhorst T, Pham TV, Piersma SR, Knol JC, et al. Changes in the urinary extracellular vesicle proteome are associated with nephronophthisis-related ciliopathies. Journal of Proteomics. 2019;192:27-36. https://doi.org/10.1016/j.jprot.2018.07.008.

Stokman MF, van der Zwaag B, van de Kar NCAJ, van Haelst MM, van Eerde AM, van der Heijden JW, et al. Clinical and genetic analyses of a Dutch cohort of 40 patients with a nephronophthisis-related ciliopathy. Pediatric Nephrology. 2018;33(10):1701-12. https://doi.org/10.1007/s00467-018-3958-7.

Sugimoto K, Miyazawa T, Enya T, Nishi H, Miyazaki K, Okada M, et al. Clinical and genetic characteristics of Japanese nephronophthisis patients. Clinical and experimental nephrology. 2016;20(4):637-49. https://doi.org/10.1007/s10157-015-1180-5.

Sun L, Tong H, Wang H, Yue Z, Liu T, Lin H, et al. High mutation rate of in 18 Chinese infantile nephronophthisis patients. Nephrology. 2016;21(3):209-16. https://doi.org/10.1111/nep.12563.

Tang C, Zhou D, Tan R, Zhong X, Xiao X, Qin D, et al. Auxiliary genetic analysis in a Chinese adolescent NPH family by single nucleotide polymorphism screening. Mol Med Rep. 2020;21(3):1115-24. https://doi.org/10.3892/mmr.2020.10917.

Tang X, Liu C, Liu X, Chen J, Fan X, Liu J, et al. Phenotype and genotype spectra of a Chinese cohort with nephronophthisis-related ciliopathy. Journal of Medical Genetics. 2022;59(2):147. https://doi.org/10.1136/jmedgenet-2020-107184.

Tang X, Xu H, Shen Q, Li G, Rao J, Chen J, et al. Gene mutation and clinical analysis of nephronophthisis diagnosed using whole exome sequencing: Experience from China Clinical nephrology. 2019;92(2):89-94. https://doi.org/10.5414/cn109571.

Taskiran EZ, Korkmaz E, Gucer S, Kosukcu C, Kaymaz F, Koyunlar C, et al. Mutations in ANKS6 Cause a Nephronophthisis-Like Phenotype with ESRD. Journal of the American Society of Nephrology. 2014;25(8). https://journals.lww.com/jasn/fulltext/2014/08000/mutations_in_anks6_cause_a_nephronophthisis_like.10.aspx.

Tayfur AC, Besbas N, Bilginer Y, Ozaltin F, Duzova A, Bakkaloglu M, et al. Follow-Up of Patients With Juvenile Nephronophthisis After Renal Transplantation: A Single Center Experience. Transplantation Proceedings. 2011;43(3):847-9. https://doi.org/10.1016/j.transproceed.2011.01.107.

Tong H, Zhao F, Yang Y, Qiu X, Zhu L, Yu Z. Scalp Tumor and Hydroureteronephrosis in Patients with Nephronophthisis and Homozygous NPHP1 Deletion. Clinical pediatrics. 2023;62(12):1508-12. https://doi.org/10.1177/00099228231162416.

Tory K, Rousset-Rouvière C, Gubler M-C, Morinière V, Pawtowski A, Becker C, et al. Mutations of NPHP2 and NPHP3 in infantile nephronophthisis. Kidney International. 2009;75(8):839-47. https://doi.org/10.1038/ki.2008.662.

Valente EM, Marsh SE, Castori M, Dixon-Salazar T, Bertini E, Al-Gazali L, et al. Distinguishing the four genetic causes of jouberts syndrome–related disorders. Annals of Neurology. 2005;57(4):513-9. https://doi.org/10.1002/ana.20422.

Wang X, Xiao H, Yao Y, Xu K, Liu X, Su B, et al. Spectrum of Mutations in Pediatric Non-glomerular Chronic Kidney Disease Stages 2-5. Frontiers in genetics. 2021;12:697085. https://doi.org/10.3389/fgene.2021.697085.

Wolf MTF, Saunier S, O'Toole JF, Wanner N, Groshong T, Attanasio M, et al. Mutational analysis of the RPGRIP1L gene in patients with Joubert syndrome and nephronophthisis. Kidney International. 2007;72(12):1520-6. https://doi.org/10.1038/sj.ki.5002630.

Yue Z, Lin H, Li M, Wang H, Liu T, Hu M, et al. Clinical and pathological features and varied mutational spectra of pathogenic genes in 55 Chinese patients with nephronophthisis. Clinica Chimica Acta. 2020;506:136-44. https://doi.org/10.1016/j.cca.2020.03.015.

Zaki MS, Sattar S, Massoudi RA, Gleeson JG. Co-occurrence of distinct ciliopathy diseases in single families suggests genetic modifiers. American Journal of Medical Genetics Part A. 2011;155(12):3042-9. https://doi.org/10.1002/ajmg.a.34173.

Online Resource 5: Study characteristics

Author (year)	Country	Study type	N	N with NPH	Age	Gender (F:M)
Ala-Mello (1996)	Finland	Retrospective	18	6	Mean: 10.4(6.8-13.8)	2:4
Ala-Mello (1998)	Finland	Retrospective	7	7	m	2:5
Ala-Mello (1999)	Finland	Retrospective	59	59	m	m
Antignac (1993)	France	Genetic/linkage analysis	22 families	18 families	m	m
Attanasio (2007)	USA	Genetic/linkage analysis	25 kindreds	m	m	m
Avci (2022)	Turkey	Retrospective	17	17	Mean: 12.6	M
Bakkaloglu (2014)	Turkey	Retrospective	4	4	10-24	2:2
Baris (2006)	USA	Genetic/linkage analysis	7	7	18 m – 12y	3:4
Beal (2024)	England	Retrospective	71	4	m	m
Birtel (2021)	m	Retrospective	16	16	Median: 17(6-53)	3:13
Bizet (2015)	m	Genetic/linkage analysis	8	7	m	m
Blowey (1996)	m	Retrospective	11	11	Mean: 9.5(6-33)	6:5
Braun (2016)	m	Genetic/linkage analysis	103	7	m	m
Caridi (1998)	Italy	Case report	8	8	Mean: 22.8(6-33)	5:3
Caridi (2000)	Italy	Genetic/linkage analysis	68	68	m	34:34
Caridi (2006)	Italy	Genetic/linkage analysis	56	56	m	m
Chaari (2012)	Tunesia	Genetic/linkage analysis	100	100	m	38:62
Chaki (2011)	m	Genetic/linkage analysis	440	m	m	m
Chaki (2012)	m	Genetic/linkage analysis	m	m	m	m
Chen (2022)	China	Genetic/linkage analysis	6	6	Mean: 4.8(1-10)	2:4
Dahmer-Heath (2021)	Germany		75	17	Median: 18(5-75)	4:13
Doreille (2021)	France	Letter to the editors	200	18	m	m
Failler (2014)	m	Genetic/linkage analysis	1255	m	m	m
Geissari (2015)	Iran	Genetic/linkage analysis	16	16	Median: 15(12-22)	10:6
Gjerstad (2023)	Norway	Retrospective	575	16	Median: 8.3(2.7-15.8)	8:8
Green (1990)	Ireland	Retrospective	1020	12	Mean: 31(9-65)	m
Grenda (2007)	m	Prospective	303	19	Mean: 11.9	m
Gretz (1998)	Germany	Retrospective	29	29	Median: 9.6(4-18.5)	13:16
Haider (1998)	Israel	Genetic/linkage analysis	1 family	10	m	3:7
Halbritter (2012)	m	Genetic/linkage analysis	192	91	m	m

Halbritter (2013)	m	Genetic/linkage analysis	1056	447	m	m
Hamiwka (2008)	Canada, USA	Retrospective	224	224	m	112:112
Hildebrandt (1997)	Germany	Retrospective	57	39	m	m
Hoefele (2005)	m	Genetic/linkage analysis	250	190	m	m
Hoefele (2007)	m	Genetic/linkage analysis	20	20	m	m
Hoefele (2011)	Turkey	Case report	4	4	Mean: 19.5(15.6-25.5)	3:1
Hoff (2013)	m	Genetic/linkage analysis	6 families	m	m	m
Hudson (2020)	Australia	Case report	4	4	m	1:3
Hussain (2018)	Pakistan	Genetic/linkage analysis	7	3	m	m
Kang (2015)	South Korea	Retrospective	5	5	Mean: 19.5(15.6-25.5)	1:4
König (2017)	Germany, Austria, the Netherlands	Retrospective	152	77	m	m
König (2022)	m	Genetic/linkage analysis	165	m	m	m
Lee (2015)	South Korea	Genetic/linkage analysis	48	6	Median: 14(1.5-21)	3:3
Li (2023)	China	Genetic/linkage analysis	29	15	m	m
Macia (2017)	France, USA, Norway	Genetic/linkage analysis	8	8	m	m
Mashat (2015)	Saudi-Arabia	Retrospective	55	3	Mean: 9.3	2:1
Medhioub (1994)	France	Genetic/linkage analysis	23 families	m	m	m
Mehrkash (2021)	Iran	Retrospective	30	7	m	m
Mistry (2007)	USA	Genetic/linkage analysis	6	6	Median: 42.5(31-55)	3:3
Murer (2002)	Italy	Immunohistochemistry analysis	17	17	Mean: 8.5	7:10
Nyberg (1995)	Sweden	Retrospective	60	10	Median: 15(10-45)	4:6
Obeidova (2020)	Czech Republic	Genetic/linkage analysis	31	1	m	m
Olbrich (2003)	m	Genetic/linkage analysis	m	38	m	m
Olinger (2003)	m	Genetic/linkage analysis	6	6	m	2:4
Omran (1999)	m	Genetic/linkage analysis	6 families	14	m	m
Omran (2000)	Venezuela	Genetic/linkage analysis	340	29	m	20:9
O´Toole (2010)	m	Genetic/linkage analysis	116 families	m	m	m
Otto (2003)	m	Genetic/linkage analysis	m	9	m	m
Otto (2005)	m	Genetic/linkage analysis	527	435	m	m

Otto (2008)	m	Genetic/linkage analysis	470	372	m	m
Otto (2009)	m	Genetic/linkage analysis	9 families	m	m	m
Otto (2010)	m	Genetic/linkage analysis	10 families	m	m	m
Otto (2011)	m	Genetic/linkage analysis	120	46	m	m
Parisi (2004)	USA	Genetic/linkage analysis	25	4	Median: 13(8-17)	2:2
Petzold (2023)	France	Genetic/linkage analysis	600	600	m	m
Qiu (2022)	China	Genetic/linkage analysis	5	m	m	m
Sakakibara (2022)	Japan	Genetic/linkage analysis	574	34	m	m
Sayer (2006)	m	Genetic/linkage analysis	25 kindreds	m	m	m
Schuermann (2002)	m	Genetic/linkage analysis	7 families	14	m	m
Shaheen (2016)	UK, Saudi Arabia	Genetic/linkage analysis	371	8	m	m
Snoek (2018)	m	Genetic/linkage analysis	5606	26	m	14:12
Soliman (2010)	Egypt	Letter to the editors	20	16	m	m
Soliman (2012)	Egypt	Retrospective	20	16	Median: 8.5(5m-12y)	10:6
Soliman (2014)	Egypt	Retrospective	105	30	m	m
Stokman (2018)	The Netherlands	Retrospective	40	18	m	m
Stokman (2019)	The Netherlands	Retrospective	12	6	Median: 13.5(6-21)	4:2
Sugimoto (2016)	Japan	Genetic/linkage analysis	35	35	Median: 14(6-46)	19:16
Sun (2016)	China	Genetic/linkage analysis	18	18	m	6:12
Tang (2019)	China	Genetic/linkage analysis	5	5	Median: 4.6(0.2-13.7)	2:3
Tang (2020)	China	Genetic/linkage analysis	10	10	Mean: 27.9(9-73)	3:7
Tang (2022)	China	Retrospective	60	27	m	m
Taskiran (2014)	Turkey	Genetic/linkage analysis	56	m	m	m
Tayfur (2011)	Turkey	Retrospective	9	9	m	4:5
Tong (2023)	China	Retrospective	4	4	Median: 12,7(12,2-13.8)	1:3
Tory (2009)	m	Genetic/linkage analysis	43 families	25	m	m
Valente (2005)	m	Genetic/linkage analysis	23 families	4	m	m
Wang (2021)	China	Genetic/linkage analysis	69	M	m	m
Wolf (2007)	m	Genetic/linkage analysis	56	0	m	m
Yue (2020)	China	Genetic/linkage analysis	55	55	Median: 7.65(1m-17y)	28:27
Zaki (2011)	Egypt	Retrospective	28	3	Median: 12(10-18)	0:3

Online Resource 6: Quality ratings grouped by quality rating tool and study type.

Study	Quality rating		
	**Good**	**Moderate**	**Poor**
**Retrospective studies evaluated with the CASP Checklist for cohort studies**			
Ala-Mello (1996)	x		
Ala-Mello (1998)		x	
Ala-Mello (1990)	x		
Avci (2022)		x	
Birtel (2021)	x		
Blowey (1996)			x
Gjerstad (2023)	x		
Green (1990)		x	
Gretz (1998)		x	
Hamiwka (2008)		x	
Soliman (2012)	x		
Soliman (2014)		x	
Stokman (2018)	x		
Stokman (2019)	x		
Tang (2022)	x		
Tayfur (2011)	x		
Zaki (2011)	x		
**Retrospective studies evaluated with the JBL Checklist for case studies**			
Bakkaloglu (2014)	x		
Tong (2023)		x	
**Prospective studies evaluated with the CASP Checklist for cohort studies**			
Dahmer-Heath (2021)	x		
Grenda (2007)	x		
**Genetic studies evaluated with the CASP Checklist for cohort studies**			
Antignac (1993)	x		
Attanasio (2007)			x
Bizet (2015)		x	
Braun (2016)	x		
Caridi (2000)		x	
Caridi (2006)	x		
Chaki (2011)		x	
Chaki (2012)		x	
Chaari (2012)	x		
Chen (2022)		x	
Geissari (2015)	x		
Haider (1998)	x		
Halbritter (2012)		x	
Halbritter (2013)		x	
Hoefele (2005)	x		
Hoefele (2007)		x	
Hoff (2013)		x	
König (2022)	x		
Lee (2015)		x	
Li (2023)	x		
Macia (2017)		x	
Medhioub (1994)		x	
Obeidova (2020)	x		
Olbrich (2003)		x	
Olinger (2003)			x
Omran (1999)		x	
Omran (2000)	x		
Otto (2003)		x	

Otto (2005)		x	
Otto (2008)	x		
Otto (2009)	x		
Otto (2010)		x	
Otto (2011)			x
Parisi (2004)	x		
Petzold (2023)	x		
Qiu (2022)	x		
Sakakibara (2022)	x		
Sayer (2006)		x	
Schuermann (2002)		x	
Shaheen (2016)	x		
Snoek (2018)	x		
Sugimoto (2016)		x	
Sun (2016)	x		
Tang (2019)		x	
Tang (2020)	x		
Taskiran (2014)			x
Tory (2009)	x		
Valente (2005)		x	
Wang (2021)	x		
Wolf (2007)	x		
Yue (2020)	x		
**Genetic studies evaluated with the JBL Checklist for case studies**			
Baris (2006)	x		
Failler (2014)	x		
Hussain (2018)	x		
Mistry (2007)	x		
O´Toole (2010)		x	
**Case reports evaluated with the JBL Checklist for case studies**			
Caridi (1998)		x	
Hoefele (2011)	x		
Hudson (2020)	x		
**Letters to the editor evaluated with the CASP Checklist for cohort studies**			
Doreille (2021)	x		
Soliman (2010)	x		
**Immunohistochemistry studies evaluated with the CASP Checklist for cohort studies**			
Murer (2002)		x	

Online Resource 7: Definition of clinical outcomes, surrogate parameter and patient-reported outcomes with reporting frequency

	Clinical outcomes			Surrogate parameter			Patient-reported outcomes		
**General**	Failure to thrive	13/90	(14%)				Fatigue	4/90	(5%)
	Laterality defects	12/90	(13%)				Pruritus	2/90	(2%)
**Cardiac**	Aortic aneurysm/ insufficiency	2/90	(2%)	Bradycardia	1/90	(1%)			
	Aortic coarctation	3/90	(3%)						
	Cardiomyopathy	11/90	(12%)						
	Congenital structural heart defect	21/90	(23%)						
	Dextocardia	1/90	(1%)						
**Endocrinology**	Delayed puberty	1/90	(1%)						
	Diabetes mellitus	3/90	(3%)						
	Growth hormon deficiency	1/90	(1%)						
	Hypogenitalism	5/90	(6%)						
	Hypogonadism	3/90	(3%)						
	Inverted nipples	1/90	(1%)						
	Polycystic ovary syndrome	2/90	(2%)						
**Gastrointestinal**	Gastrointestinal manifestation	2/90	(2%)				Nausea	1/90	(1%)
	Pancreatic cysts	4/90	(4%)						
**Kidney**	CAKUT disorders	12/90	(13%)	Delayed tooth eruption	2/90	(2%)	Enuresis	12/90	(13%)
	Edema	2/90	(2%)	GFR	64/90	(71%)	Nocturia	3/90	(3%)
	End stage kidney disease	75/90	(83%)				Polydipsia/polyuria	35/90	(39%)
	Hematuria	4/90	(4%)				Salt craving	1/90	(1%)
	Histology	49/90	(54%)						
	Isosthenuria	1/90	(1%)						
	Kidney cysts	47/90	(52%)						
	Kidney echogenicity	39/90	(43%)						
	Kidney size	30/90	(33%)						
	Kidney volume	1/90	(1%)						

	Nephrocalcinosis	2/90	(2%)						
	Nephrotic syndrome	1/90	(1%)						
	Proteinuria	22/90	(24%)						
	Renal tubular acidosis	2/90	(2%)						
	Renomegaly	1/90	(1%)						
	Urinary tract infection	3/90	(3%)						
	Urine concentration defect	15/90	(17%)						
**Lab/blood**	Anemia	23/90	(26%)	Acedosis	1/90	(1%)			
	Dyselectrolytemia	4/90	(4%)	Alkaline phosphatase	2/90	(2%)			
	Hyperlipidosis	1/90	(1%)	Blood urea nitrogen (BUN)	3/90	(3%)			
	Hypertension	27/90	(30%)	Infection	3/90	(3%)			
				Phosporus	3/90	(3%)			
				Serum creatinine	28/90	(31%)			
**Liver**	Cholestatic hepatopathy	23/90	(26%)	Esophageal varices	2/90	(2%)			
	Fatty liver	1/90	(1%)	Hepatic pruritus	2/90	(2%)			
	Hepatomegaly	13/90	(14%)	Liver disease	26/90	(29%)			
	Liver cysts	8/90	(9%)	Thrombopenia	1/90	(1%)			
	Liver fibrosis	35/90	(39%)						
	Splenomegaly	4/90	(4%)						
**Lungs**	Pulmonary hypoplasia	2/90	(2%)						
**mental development/ neurological disease**	Ataxia	5/90	(5%)	IQ	1/90	(1%)	Dizziness	2/90	(2%)
	Cerebrovascular disease	3/90	(3%)						
	Congenital contractures	1/90	(1%)						
	Epilepsy	12/90	(13%)						
	Hearing loss / impairment	13/90	(14%)						
	Hypotonia	13/90	(14%)						

	Infratentorial congenital brain malformation	27/90	(30%)
	MRI brain performed	11/90	(12%)
	Neural tube defects	6/90	(7%)
	Neurodevelopmental disorders	39/90	(43%)
	Peripheral neuropathy	3/90	(3%)
	Sense of smell (anosmia, hyposmia)	2/90	(2%)
	Spasticity/ophistotonus	2/90	(2%)
	Supratentorial congenital brain malformation	12/90	(13%)
	Tremor	2/90	(2%)
**Oncology**	Neoplasm	7/90	(8%)
**Opthalmological**	amblyopia	1/90	(1%)
	anisocoria	1/90	(1%)
	cataracts	4/90	(4%)
	color blindness	2/90	(2%)
	eye movement disorders	20/90	(22%)
	LCA leber congenital amaurosis	11/90	(12%)
	night blindness	5/90	(6%)
	nystagmus	19/90	(21%)
	occular involvement	5/90	(6%)
	oculocerebral disease	1/90	(1%)
	optic nerve disorders	5/90	(6%)
	outer eye abnormalities	5/90	(6%)
	reduced vision	35/90	(39%)
	refractory error	4/90	(4%)
	retinal abnormalities	53/90	(59%)
	strabismus	15/90	(17%)
	structural eye malformations	18/90	(20%)

**Pain**				Abdominal pain	1/90	(1%)
				Back pain	1/90	(1%)
**Prenatal**	Oligohydramnios	1/90	(1%)			
**Respiratory**	Apnea	2/90	(2%)			
	Asthma	2/90	(2%)			
	Bronchiectasis	1/90	(1%)			
	Respiratory distress	4/90	(4%)			
	Respiratory infections	11/90	(12%)			
	Sleep apnea	1/90	(1%)			
	Tachypnea	8/90	(9%)			
**Skeletal**	Chonodrodysplasia	1/90	(1%)			
	Craniofacial abnormalities	15/90	(17%)			
	Dental malformations	2/90	(2%)			
	Hyperextensibility elbows/knees	2/90	(2%)			
	Skeletal malformations	33/90	(37%)			
**Skin**	Albismus	1/90	(1%)			
	Atopy	1/90	(1%)			
	Cafe au lait macules	1/90	(1%)			
	Cholesteatoma	2/90	(2%)			
	Ichtyosis	1/90	(1%)			
**Social**				Impact daily life activities	1/90	(1%)
